# Supplementary material for: Dissecting the sequence determinants for dephosphorylation by the catalytic subunits of phosphatases PP1 and PP2A
Source: Nat Commun. 2020 Jul 17;11:3583. doi: 10.1038/s41467-020-17334-x (PMC7367873; doi:10.1038/s41467-020-17334-x)
Supplement: Supplementary file 1 — Supplementary Information [file 41467_2020_17334_MOESM1_ESM.pdf]

Supplementary Information

Dissecting the sequence determinants for dephosphorylation by the catalytic subunits of phosphatases PP1 and PP2A

Hoermann et al.

This PDF file includes:

Supplementary Figures 1 to 10
Supplementary Tables 1 to 3
Supplementary References

Other Supplementary Materials for this manuscript include the following:

Supplementary Data 1, 2 and 3
Supplementary Movies 1 to 9
Source Data

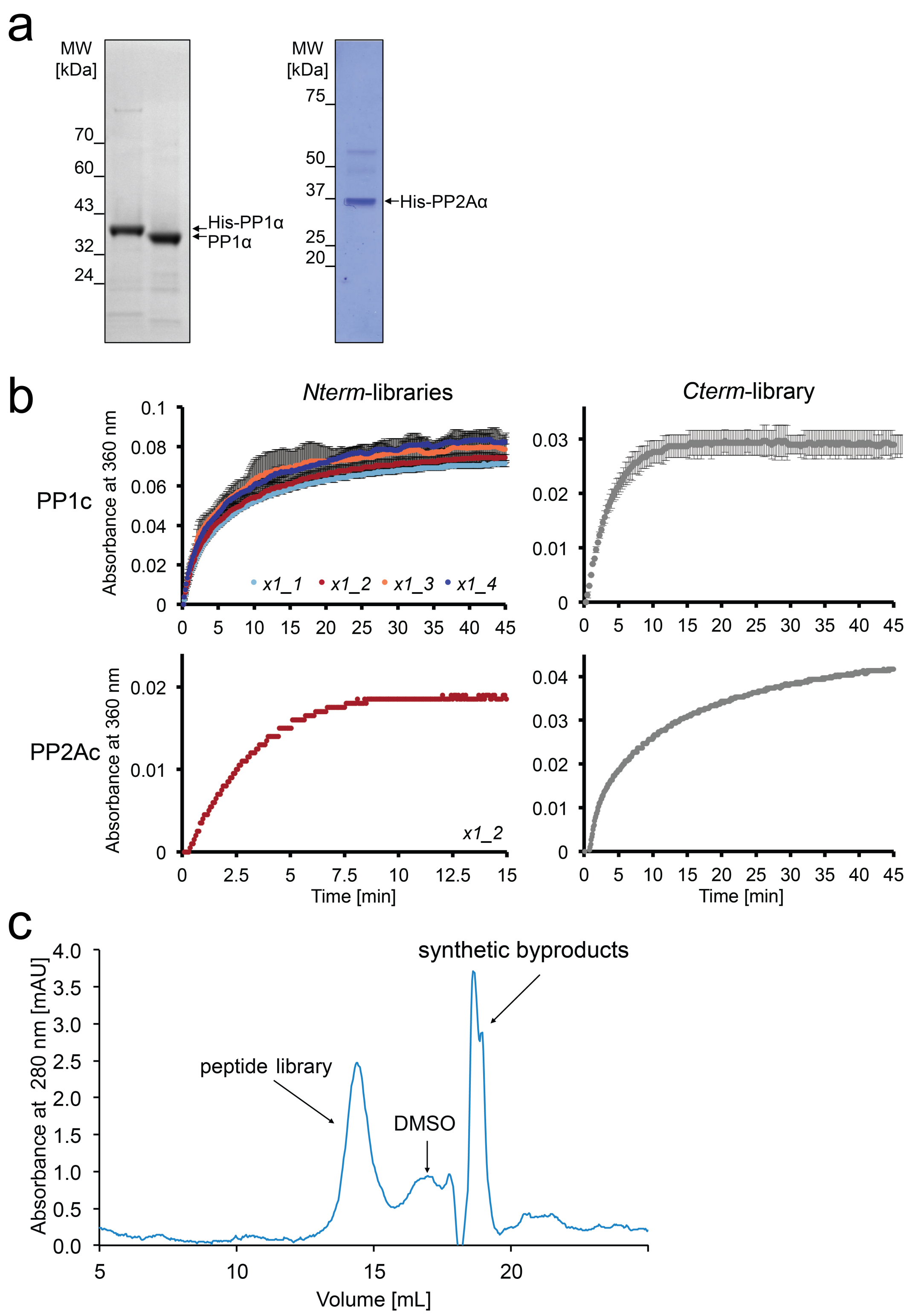

Supplementary Figure 1. Development of the PLDMS approach. a, Coomassie stained gels of the recombinant proteins used for this study. **b,** Phosphatase treatment of libraries leads to release of inorganic phosphate. This can be detected by a secondary enzyme reaction. The purine nucleoside phosphorylase (PNP) enzyme converts 2-amino-6-mercapto-7-methylpurine riboside

(MESG) substrate to the ribose 1-phosphate and 2-amino-6-mercapto-7-methylpurine products under the consumption of inorganic phosphate[1]. This reaction is accompanied by a spectrophotometric shift in absorbance from 330 nm (educt) to 360 nm (product). Based on these results, proceeding phosphatase reactions were stopped by a pH shift to 3 at 2 min and 4 min for PP1c and PP2Ac, respectively, to limit the dephosphorylation process to preferred substrate sequences as outlined in Figure 1a. For PP1c, the average of biological triplicates with three datapoints each is blotted. Error bars represent the s.d. of averages. Due to the limited availability of recombinant human PP2Ac, dephosphorylation assays were only carried out once, therefore, the average of technical triplicates is blotted. **c,** Samples were then loaded onto a size exclusion column to separate treated library samples or untreated controls from the recombinant phosphatase and eluted in 0.1 M ammonium acetate using a fraction collector. To identify peptides, absorption at 280 nm was monitored. The peptide-containing fractions were pooled and concentrated in a SpeedVac centrifuge prior to MS analysis. Other peaks represent assay buffer components or peptide synthesis byproducts (determined by HPLC-MS, data not shown). Source data are provided as a Source Data file.

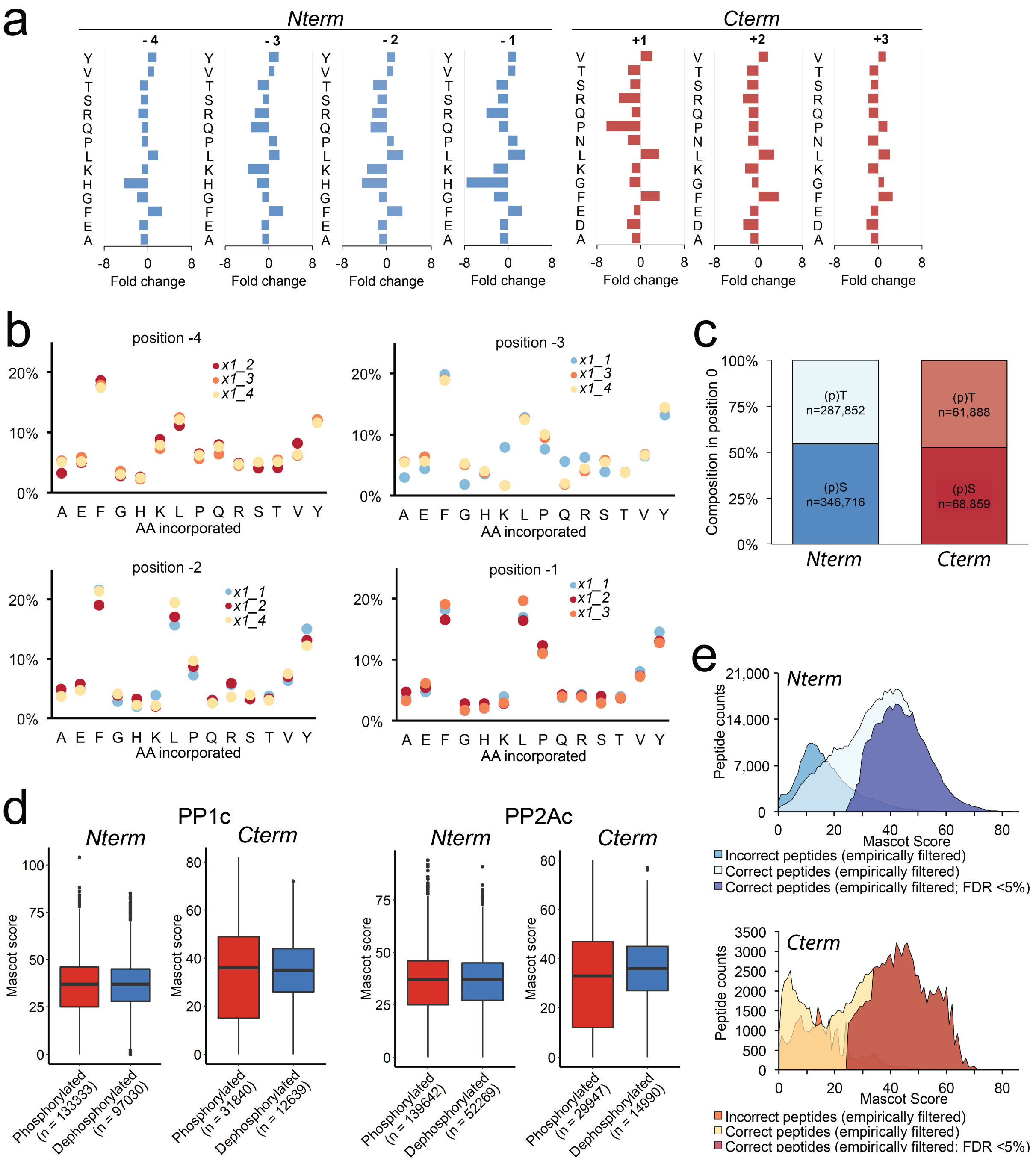

Supplementary Figure 2. Analysis of the PLDMS approach. As in the reference measurements (Fig. 1), raw data was filtered for empirically correct sequences (Supplementary Table 3, data of the *Nterm* libraries was combined). Comparison of values in Supplementary Table 3 with the reference measurement (Supplementary Table 2) showed that no peptides and no diversity were lost throughout the experimental procedures. **a,** Within the amino acid mixture *x1* for *Nterm* and *x2* for *Cterm*, a perfectly equal incorporation of all 14 amino acids would result in 7% for each amino acid. In order to test if the distribution of amino acids in the synthetic route

would also allow analysis of all amino acids in every mutated position, fold changes from 7% per amino acid for all empirically correct events were calculated. For the following heat-map analysis (Fig. 2b), an equal distribution was not required, only sufficient counts of each amino acid in every position. **b,** To assess the reproducibility of the synthetic route, the four independently synthesized *Nterm*-libraries were analyzed for their amino acid distribution in the three mutated residues and compared to each other. **c,** Average incorporation ratios of (p)S/(p)T in position 0 of *Nterm* and *Cterm* calculated from all empirically correct peptides. Data was obtained from the analysis of 634,568 peptides for *Nterm* and 130,747 peptides for *Cterm* samples. We did not observe any unspecific hydrolysis of peptides in our synthesis and assay setup (Supplementary Table 3). **d,** Phosphorylation state does not affect the Mascot score. Boxplots highlight the median and 1st/3rd quartile. Whiskers mark the lowest and highest data points that are within a 1.5-fold interquartile range. **e,** Mascot score distribution of detected peptides of *Nterm*-libraries (left) and *Cterm*-library (right). Filtering for expected peptides allowed to distinguishing between populations of correct and incorrect peptides. Importantly, we additionally manually excluded empirically incorrect peptides (i.e. peptides with sequences not matching expected sequences from the synthetic route) from all further analysis, thereby further decreasing the FDR. The results of this filtering are also shown in Supplementary Table 3 and Supplementary Data 1. Our analysis procedure resulted in library coverage, over the different treatments, of 46-48% (without FDR: 70-78%) for the averaged *Nterm*-libraries, and 38-43% (without FDR: 50-55%) for *Cterm*-libraries (see Supplementary Table 3). These peptides then entered further analysis. Source data are provided as a Source Data file.

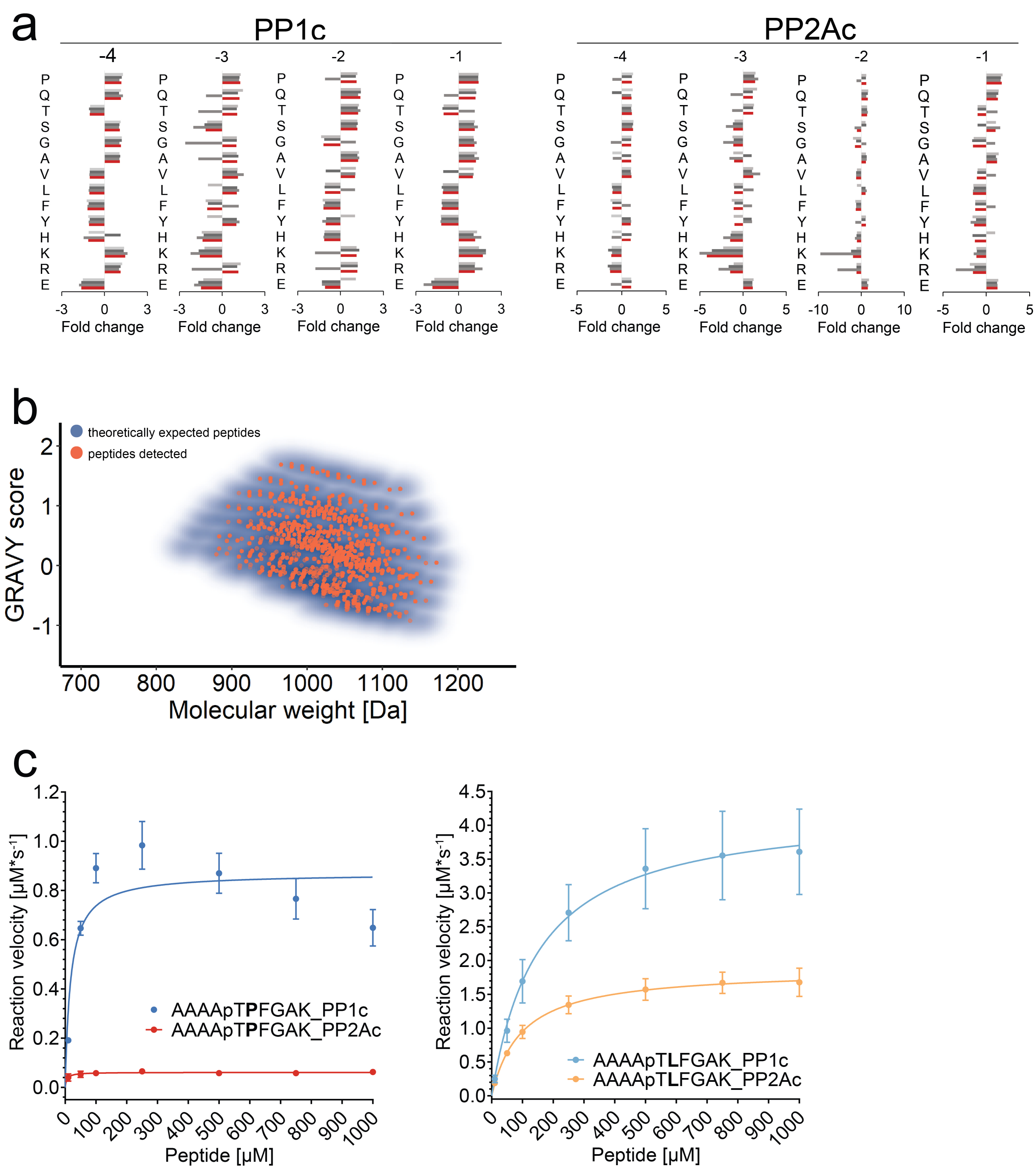

k_{cat}/K_m [$s^{-1}µM^{-1}$]	PP1c	PP2Ac	ratio PP1c:PP2Ac
P	0.45 ± 0.029	0.013 ± 0.019	34:1
L	0.64 ± 0.13	0.33 ± 0.04	2:1

Supplementary Figure 3. Quality assessment and verification of the PLDMS approach. a, *Nterm*-libraries *x1_1* to *x1_4* were also analyzed independently to assess the reproducibility of our findings. Each position is permutated in 3 out of 4 libraries and the amino acid preference for PP1c was analyzed in these 3 positions for each sub-library separately. Each grey bar represents

a single library. The red bar represents the values from the combined analysis of libraries *x1_1* to *x1_4* also depicted by the heat-map in Figure 2b. **b,** Analysis of theoretical versus measured peptides using the grand average of hydropathy value (GRAVY)[2] showed that our library synthesis covered the whole range of possible polarity properties. **c**, Kinetic analysis of the dephosphorylation rate upon PP2Ac or PP1c of two synthetic peptides, chosen to confirm the effect of Pro in +1 of the heat-maps presented in Figure 2b. Error bars represent s.e.m. of three independent repeats with technical duplicates. k_{cat}/K_m was calculated by comparison to a phosphate standard curve. Source data of Fig. 3b,d are provided as a Source Data file.

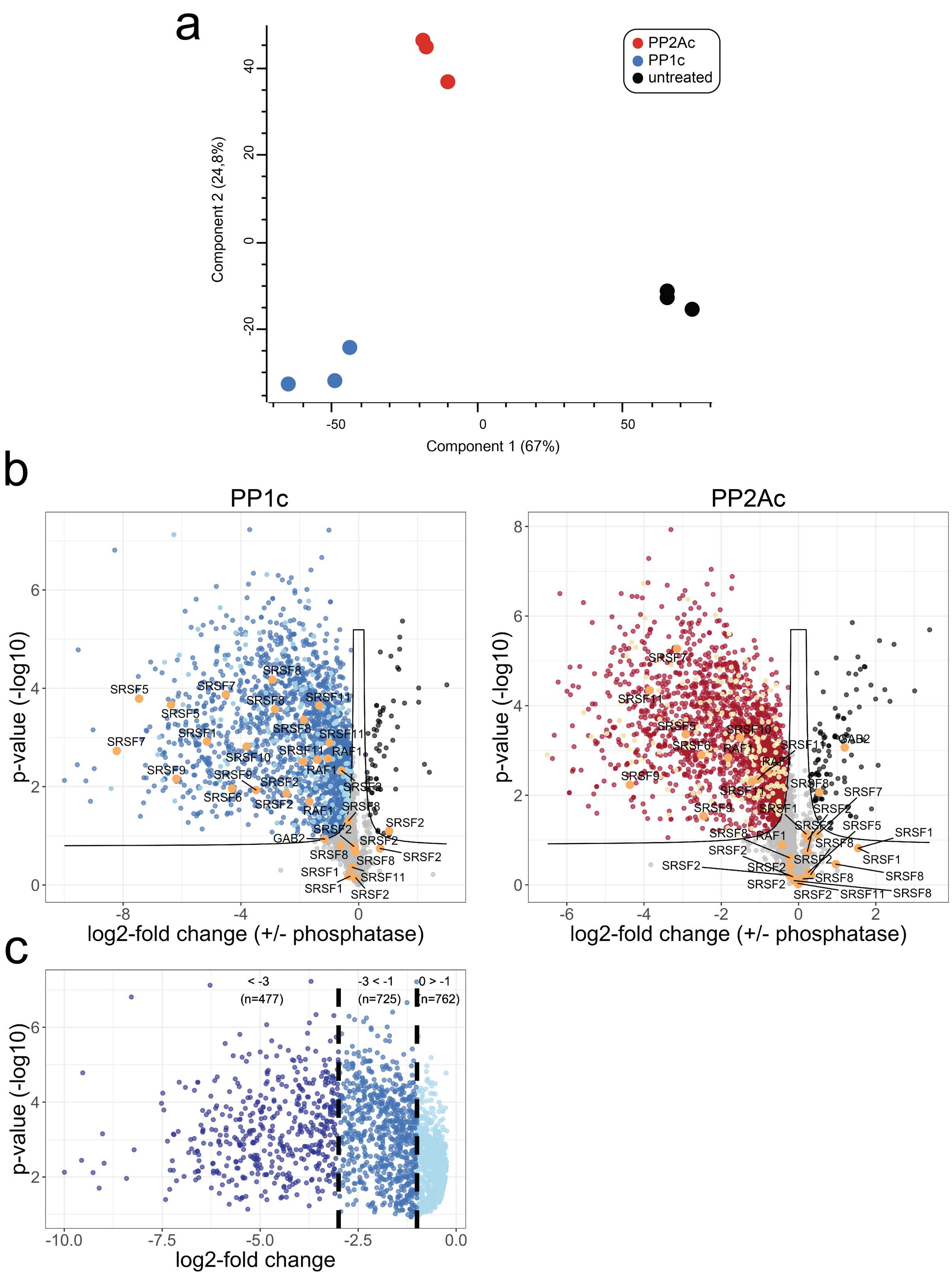

Supplementary Figure 4. Analysis of phosphoproteomic data. a, Principal component analysis (PCA) of samples in the software Perseus using standard settings. **b,** p-sites significantly

changing (two-sided Student's t-test, FDR 0.01) upon PP1c or PP2Ac treatment. P-sites highlighted in light-blue/yellow correspond to annotated regulatory p-sites from PhosphositePlus (v03/07/18). All p-sites on SRSFs and GAB2 (not only the selective ones shown in Table 1) are highlighted in orange. See also Supplementary Data 2 for details. **c,** PP1c-sensitive pS/pT-sites from Supplementary Figure 4b were classified in three groups dependent on the log2-fold decrease in phosphorylation as indicated for PP1c-sensitive sites in Supplementary Figure 4c. This classification was done in parallel for PP1c and PP2Ac and was then used for further analysis shown in Figure 5a.

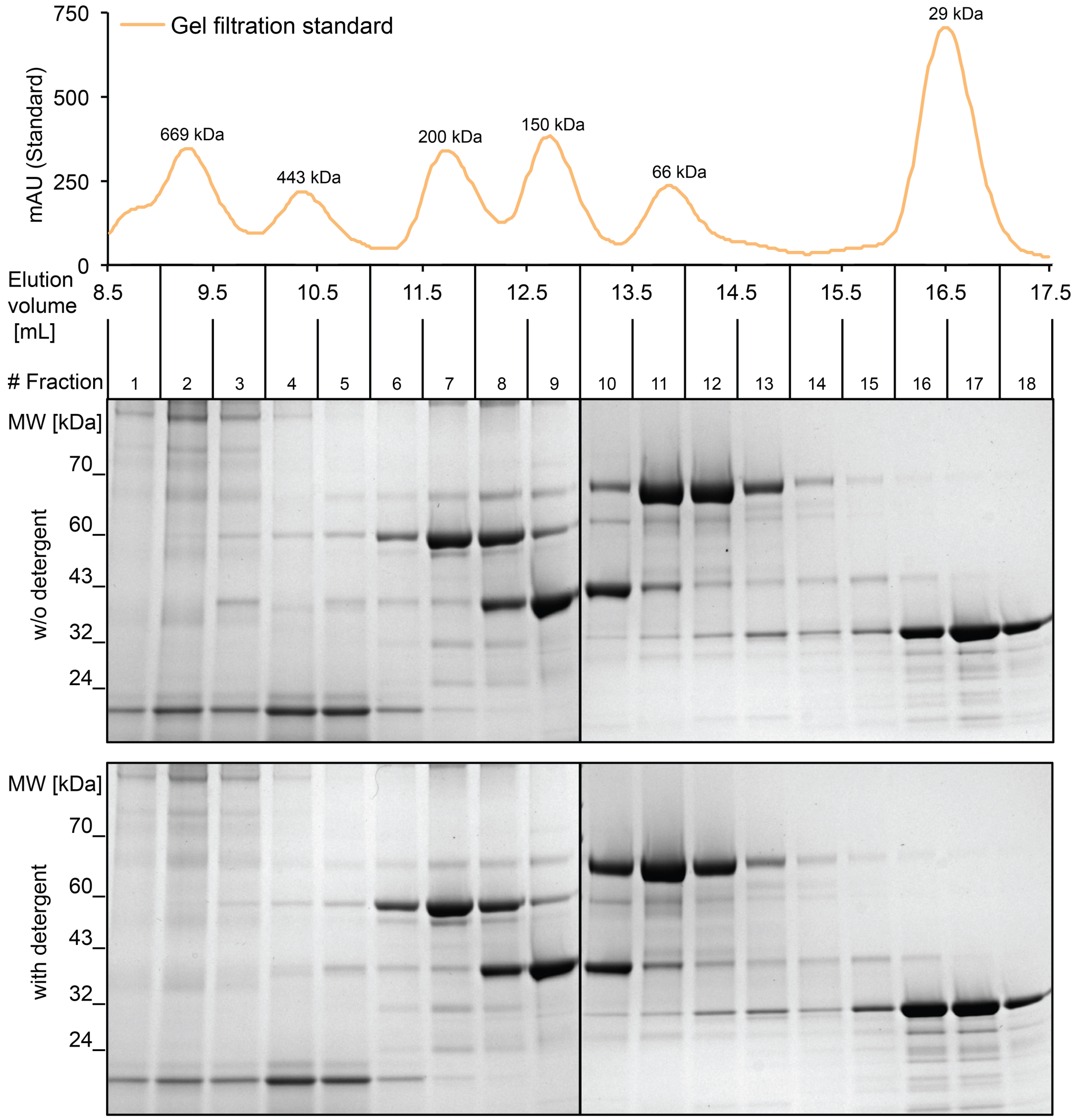

Supplementary Figure 5. Analysis of gel filtration standard mix by size exclusion chromatography. To control for holoenzyme formation of PP1c and PP2Ac in the course of the incubation with lysate from HeLa Kyoto cells (see Fig. 3a and Methods for assay details), a comparison to gel filtration standard proteins was carried out. Since the lysis buffer for the phosphoproteomic experiments contains 0.1% of the UV-absorbing detergent NP-40/IGEPAL (see Methods), analysis of elution profiles by detection of absorbance at 280nm was not possible (top). Therefore, the standard proteins were injected in assay buffer with or without detergent and analyzed by Coomassie staining to demonstrate that the use of detergent does not impact retention time. A mix of carbonic anhydrase (29 kDa), albumin (66 kDa), alcohol dehydrogenase (150 kDa), β-amylase (200 kDa), apoferritin (443 kDa), and thyroglobulin (669 kDa) was prepared in 100 mM NaCl, 10 mM Tris and analysed on a Superdex 200 Increase 10/300 column using an ÄKTA-Explorer system. Several of these proteins are complexes (e.g. the 150 kDa alcohol dehydrogenase is a tetramer of four equal subunits), which dissociate in denaturing

conditions during gel-electrophoresis during the analysis of the fractions. Different standard proteins were compared for test experiments and elution volumes were constant. Therefore, the final setup was carried out once. Source data are provided as a Source Data file.

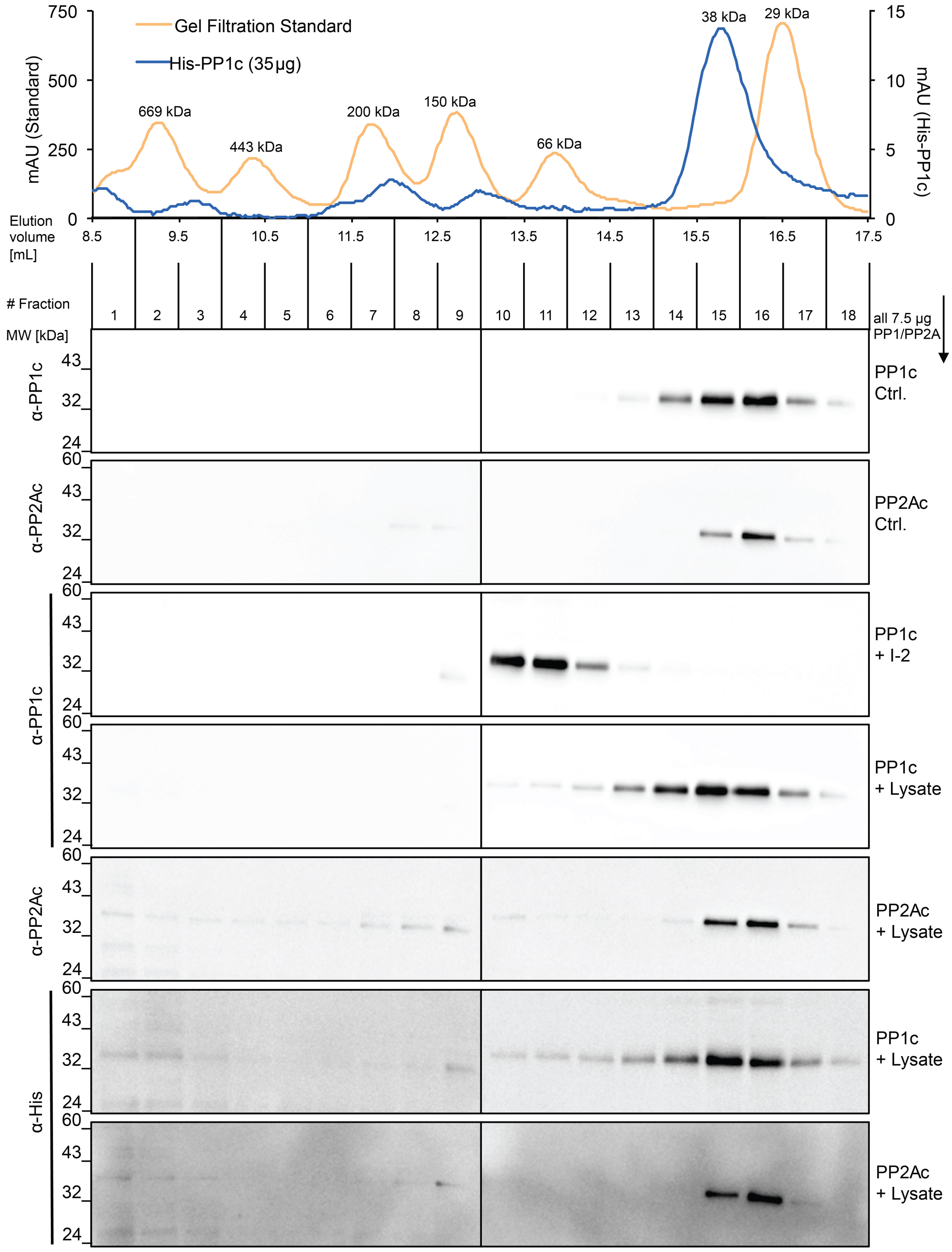

Supplementary Figure 6. Gel filtration analysis to control for PP1c and PP2Ac holoenzyme formation during incubation with HeLa Kyoto cell lysate. The dephosphorylation reaction was set up exactly as described for phosphoproteomics in Figure 3a. Instead of denaturing the

proteome in 8M urea and subsequent phosphoproteomic analysis, samples were analyzed by gel filtration using a Superdex 200 Increase 10/300 column on an ÄKTA-Explorer system. For generating the presented UV-traces, gel filtration standard mix or 35 μg of pure recombinant His-PP1c were injected and elution profiles were detected at 280 nm in assay buffer without detergent. As demonstrated by Coomassie staining of standard proteins in Supplementary Figure 5, including detergent does not change the elution profile. All runs analyzed by western blotting contained 7.5 μg PP1c/PP2Ac, corresponding to 1 μM used together with 1 mg total cell lysate in the dephosphorylation assay for phosphoproteomics. To demonstrate the ability of the experimental setup to preserve holoenzymes, PP1c was also incubated with an equimolar amount of the regulator Inhibitor 2 (I-2, IPP2, PPP1R2, see Supplementary Fig. 7) resulting in a clear shift towards higher molecular weight in the elution profile. For being able to distinguish between potential holoenzymes formed by endogenous PP1c and PP2Ac, the His-tag of PP1c was retained and α-His antibody was used to discriminate between these possibilities. Since more than 30 μg of phosphatase are required to obtain peaks at 280nm at the UV-detector and given the limited amount of available PP2Ac, no UV-trace was acquired for PP2Ac, but the elution profile can be compared to the runs analyzed by Western blotting. Since elution profiles for SEC were highly reproducible for standard proteins and PP1c in test experiments, the final experiment was carried out once. Source data are provided as a Source Data file.

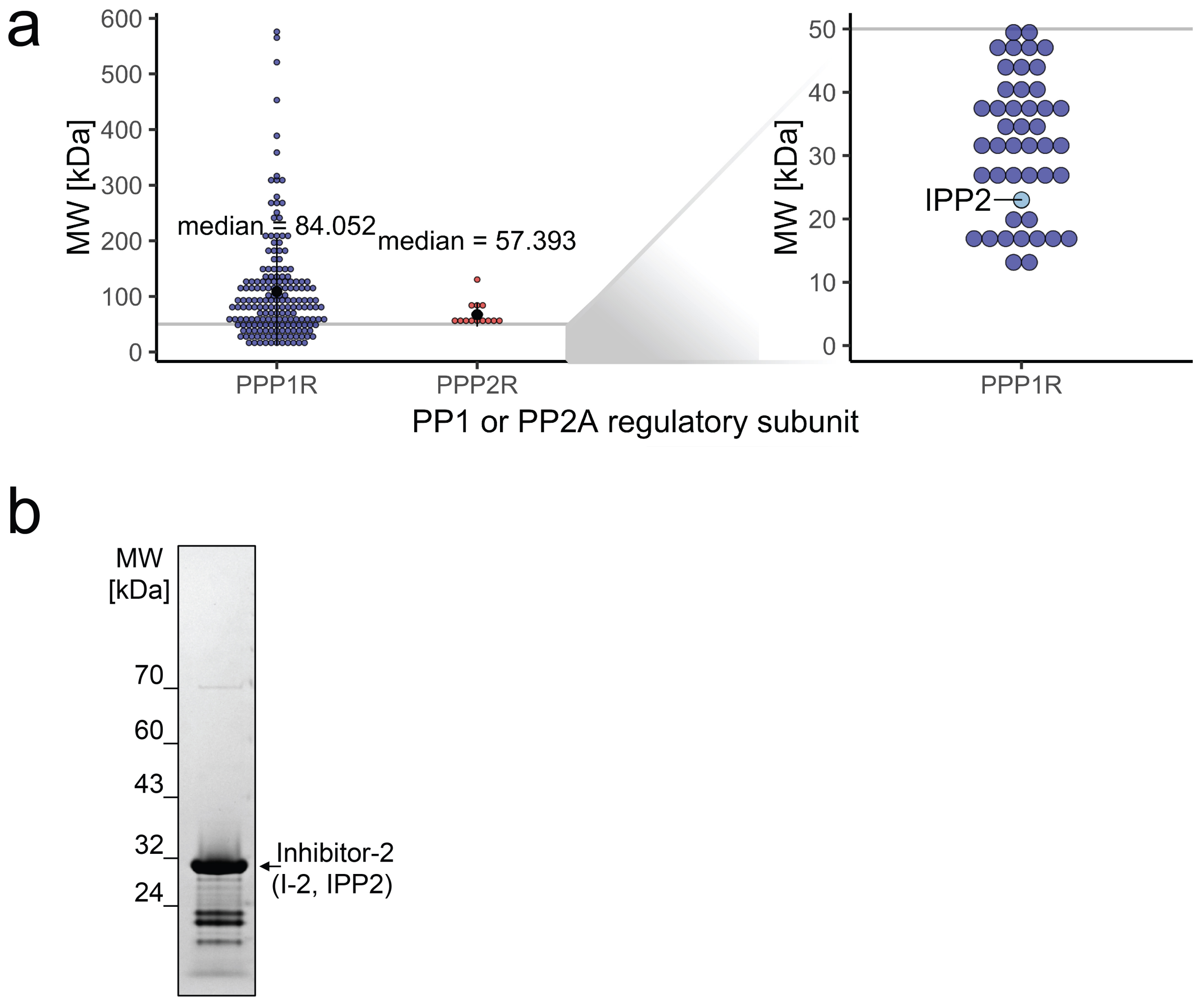

Supplementary Figure 7. Analysis of PP1/PP2A regulatory subunits. a, To demonstrate the size shift expected upon association of PP1c/PP2Ac with regulatory subunits in the dephosphorylation assay of HeLa Kyoto cell lysate, manually curated, well-annotated sets of PP1/PP2A regulatory subunits were downloaded from the website of the HUGO Gene Nomenclature Committee (HGNC, genenames.org, assessed Feb. 2020). Subsequently, HGNC IDs were correlated with Uniprot IDs using Uniprot's webtool and the molecular weight of the respective canonical isoform was extracted and blotted. This yielded a dataset of 181 PP1 regulatory subunits and 15 PP2A regulatory subunits entering final analysis. Inhibitor-2 (I-2, IPP2, PPP1R2) is highlighted in the right panel. Black dot marks the mean, error bars refer to s.d. **b,** Inhibitor-2 (I-2, IPP2, PPP1R2, sequence from rabbit) cloned into pETM20, overexpressed in *E. coli* and purified using the His-tag and Nickel affinity purification[3] was used together with PP1c as a positive control in Supplementary Figure 6. Source data are provided as a Source Data file.

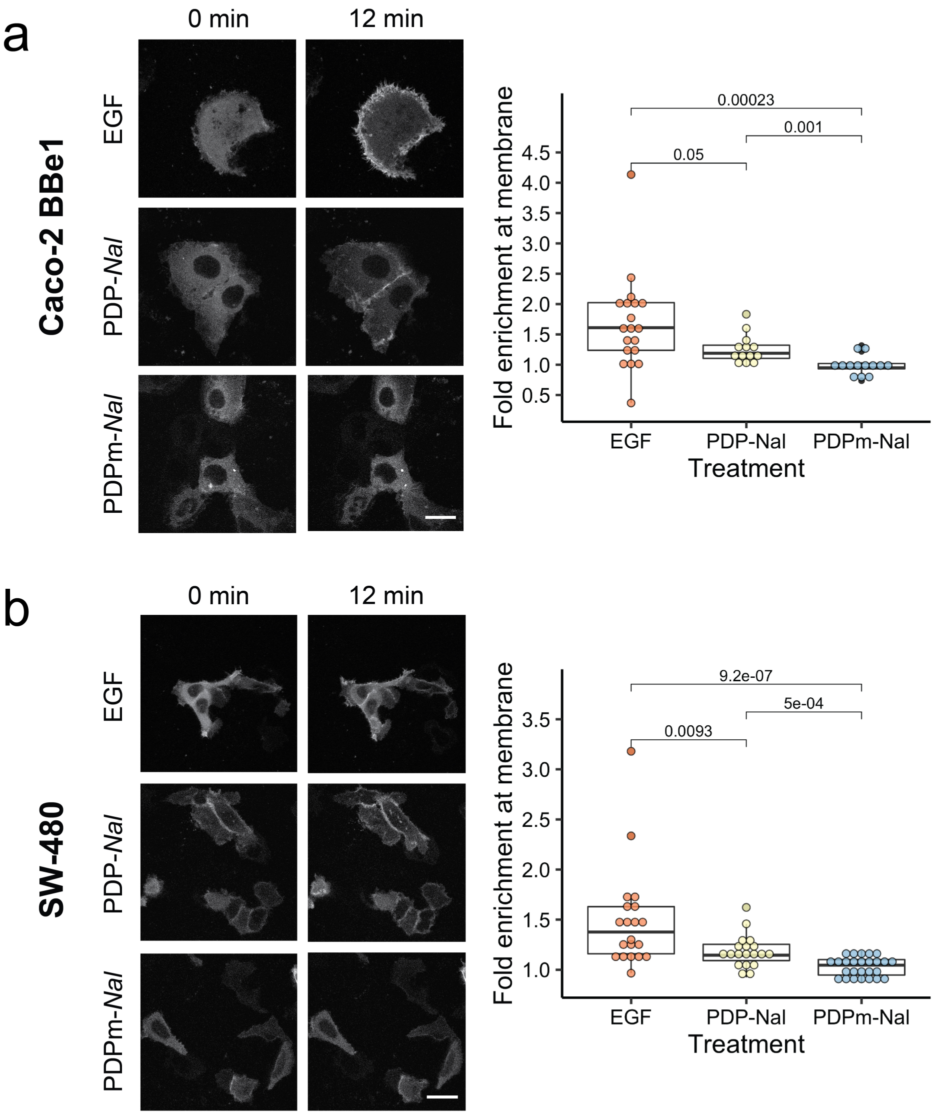

Supplementary Figure 8. The PP1-activating peptide PDP-*Nal* leads to recruitment of mKate2 –GAB2 to the cellular periphery. a,b, Caco-2 (BBe1) and SW-480 were seeded in chambers for live-cell microscopy at least 48 h before the microscope and were transfected with 100 ng pmKate2-GAB2 24 h before imaging using Fugene HD. Cells were serum-starved for 45 min-1h prior to imaging. After 45 s (frame 3), EGF or PDP-*Nal*/PDPm-*Nal* was added to a final concentration of 100 ng/mL (EGF) or 50 µM (PDP-*Nal,* PDPm-*Nal*) and GAB2 signal was observed for 12.5 min (see Supplementary Movies 4-9). Image analysis was carried out by extracting raw images at the indicated time points and was carried out in Fiji using the procedure described for HeLa Kyoto cells in Figure 6d. For Caco-2 BBe1 the following number (n) of cells were analyzed: 19 (EGF), 13 (PDP-*Nal*), 13 (PDPm-*Nal*); for SW-480: 20 (EGF), 19 (PDP-*Nal*), 26 (PDPm-*Nal*). Statistical analysis was carried in R and indicated values correspond to BH-adjusted p-values of a two-sided Wilcoxon test. Scale bars represent 25 µm. Boxplots highlight median and1st/3rd quartile. Whiskers mark the lowest and highest data points that are within a 1.5-fold interquartile range. Source data are provided as a Source Data file.

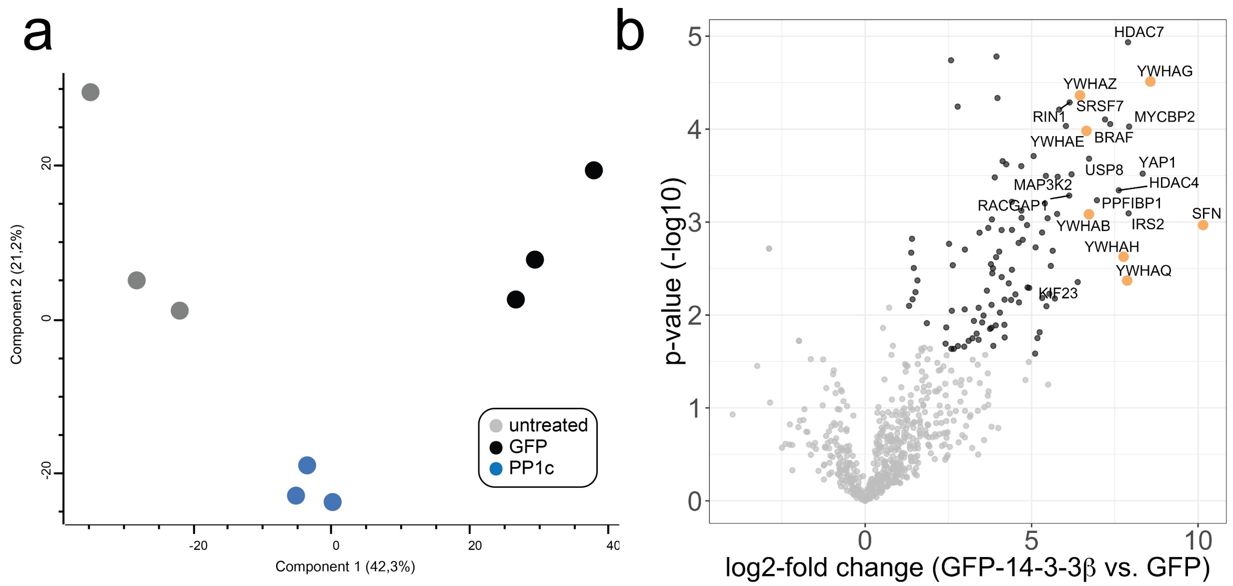

Supplementary Figure 9. Results of GFP–14-3-3-CoIP MS analysis. a, Principal component analysis (PCA) of samples using the Perseus software and standard settings. **b,** Comparison of proteins enriched in GFP-14-3-3β CoIP compared to GFP-samples. 14-3-3 isoforms are highlighted in orange and top hits are labeled. Proteins significantly enriched compared to GFP-only control samples are depicted in black (two-tailed Student's t-test, FDR 0.05). Quantitative analysis of MS data was carried out using the software Perseus v1.5.8.5 and graphs were produced in R/ggplot2.

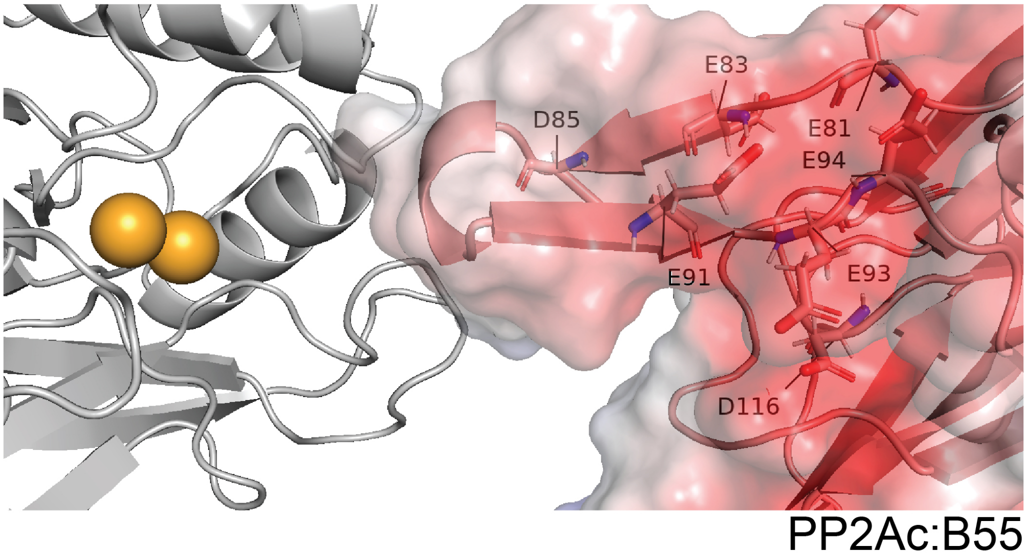

Supplementary Figure 10. The interaction of B55/PR55 with the catalytic subunit creates a basophilic environment close to the catalytic cleft. Interaction of PP2Ac (grey, spheres represent Mn^{2+}) with B55/PR55 based on the crystal structure of the trimeric holoenzyme with the scaffolding subunit A (PR65) derived PDB ID 3DW8[4]. Negatively charged amino acids within the binding region of B55 to PP2Ac are highlighted. The crystal structure of the trimeric PP2A/PR55/PR65 holoenzyme (PDB ID 3DW8) was inspected in PyMOL (v2.3.3) and the electrostatic surface of the B55 subunit was calculated using the APBS plugin with standard settings.

SUPPLEMENTARY TABLES

Supplementary Table 1. Overview of reference measurements for the PLDMS approach. Analysis of all measured events of two reference measurements after several rounds of library optimization illustrating the rational of empirical filtering: Events were analyzed for peptides with a length other than 10 amino acids, unexpected/false sequence, prediction of multiple phosphorylations or phosphorylations in positions other than 0. Since libraries were not subjected to phosphatase treatment, events with no phosphorylation were also considered false events. */** cross-reference Supplementary Tables 1 and 2 for better understanding.

	Nterm*-library *x1_1		***Cterm*-library**	
total measured events	28,949	100.0%	23,270	100.0%
false length	945	3.3%	1,719	7.4%
false sequence	0	0.0%	47	0.2%
no phosphorylation	95	0.3%	138	0.6%
two phosphorylations	360	1.2%	504	2.2%
false phosphorylation	5,530	19.1%	1,687	7.2%
total correct events	22,019*	76.1%	19,175**	82.4%
total false events	6,930	23.9%	4,095	17.6%

Supplementary Table 2. Analysis of Mascot score for an FDR of 5% and library coverage. Empirically correct/wrong sequences relate to categorization made in Supplementary Table 1 as indicated by */**. The results of this analysis are also depicted in Figure 1d.

**Library**	***Nterm-x1_1***	***Cterm***
detected peptides	28,949	23,270
average score	35	34
wrong peptides (empirically filtered)	6,930	4,095
Empirically correct peptides (EmpCorr)	22,019*	19,175**
EmpCorr phosphorylated	22,019	19,175
unique sequences in EmpCorr	3,091	1,917
library coverage	56.3%	34.9%
score at FDR <5%	39	32
EmpCorr with score at FDR <5%	12,759	12,420
of these phosphorylated	12,759	12,420
different sequences at FDR <5%	2,319	1,684
library coverage (FDR <5%)	42.3%	30.7%

Supplementary Table 3. MS analysis of dephosphorylation assays in the PLDMS approach. Analysis of all measurements in final assay experiments including phosphatase treatments following the same principle as depicted in Supplementary Table 2. Notably, different from reference measurements in the phosphatase-treated samples presented herein, unphosphorylated peptides are also considered a correct event. For a better overview, all four *Nterm* libraries were summarized in one column. The results of this analysis are also depicted in Supplementary Figure 2e. A library- and sample-specific analysis of all Mascot Score cut-offs is shown in Supplementary Data 1.

**Treatment**	**PP1_*Nterm***	**PP2A_*Nterm***	**Untreated_*Nterm***	**PP1_*Cterm***	**PP2A_*Cterm***	**Untreated_*Cterm***
detected peptides	284,994	245,996	303,770	53,262	54,368	52,505
average score	32	27	33	30	30	32
incorrect peptides (empirically filtered)	54,631	54,085	91,476	8,783	9,431	11,174
correct peptides (empirically filtered; EmpCorr)	230,363	191,911	212,294	44,479	44,937	41,331
EmpCorr dephosphorylated	97,030	52,269	NA	12,639	14,990	NA
EmpCorr phosphorylated	133,333	139,642	212,294	31,840	29,947	41,331
different sequences in EmpCorr	14,344	13,904	15,324	2,951	3,010	2,732
library coverage	72.8%	70.5%	77.7%	53.8%	54.8%	49.8%
score at FDR <5%	32	34	33	25	25	33
EmpCorr; FDR <5%	147,241	111,897	138,295	30,700	30,697	25,066
EmpCorr; FDR <5% - phosphorylated	85,118	79,206	138,295	20,969	18,660	25,066
EmpCorr; FDR <5% - dephosphorylated	62,123	32,691	NA	9,731	12,037	NA
different sequences at FDR <5%	9,485	9,072	9,513	2,337	2,362	2,065
library coverage; FDR <5%	48.1%	46.0%	48.3%	42.6%	43.0%	37.6%

SUPPLEMENTARY REFERENCES

1. Salvi, F. *et al*. Effects of stably incorporated iron on protein phosphatase-1 structure and activity. *FEBS Lett.* **592**, 4028–4038 (2018).
2. Kyte, J. & Doolittle, R. F. A simple method for displaying the hydropathic character of a protein. *J. Mol. Biol.* **157**, 105–32 (1982).
3. Wang, Y. *et al.* Interrogating PP1 activity in the MAPK pathway with optimized PP1-disrupting peptides. *ChemBioChem.* **20**, 66–71 (2019).
4. Xu, Y., Chen, Y., Zhang, P., Jeffrey, P. D. & Shi, Y. Structure of a Protein Phosphatase 2A holoenzyme: Insights into B55-mediated tau dephosphorylation. *Mol. Cell* **31**, 873–885 (2008).
