# Supplementary material for: Dissecting the sequence determinants for dephosphorylation by the catalytic subunits of phosphatases PP1 and PP2A
Source: Nat Commun. 2020 Jul 17;11:3583. doi: 10.1038/s41467-020-17334-x (PMC7367873; doi:10.1038/s41467-020-17334-x)
Supplement: Supplementary file 3 — Description of Additional Supplementary Files [file 41467_2020_17334_MOESM3_ESM.docx]

**Description of Additional Supplementary Files**

File name: Source Data

Description: **Source Data.** Contains data underlying Figures 1c,d; 2a-e, 3b,d; 4a,b; 5a; 6a,b,e and Supplementary Figures 1a-c; 2a-e; 3a-c; 5, 6, 7a,b and 8a,b, data for quality control of directed peptide synthesis, and certificates for cell line authentication.

File name: Supplementary Movie 1

Description: **Live-cell imaging of mKate2-GAB2 localization during EGF stimulation in HeLa Kyoto cells.** Images presented in Fig. 6c are based on this movie. As described in the corresponding figure legend, time-lapse-experiments consisted of an image acquisition interval of 15 s for at least 12 min for each sample condition separately using an exposure time of 0.5 frames per second. Cells were treated after frame 3 (45 s) by adding 100 µL of starvation media with 300 ng/mL EGF resulting in a final concentration of 100 ng/mL.

File name: Supplementary Movie 2

Description: **Live-cell imaging of mKate2-GAB2 localization during PDP-*Nal* stimulation in HeLa Kyoto cells.** Images presented in Fig. 6c are based on this movie. Cells were treated after frame 3 (45 s) by adding 100 µL of starvation media with 150 µM PDP-*Nal* resulting in a final concentration of 50 µM.

File name: Supplementary Movie 3

Description: **Live-cell imaging of mKate2-GAB2 localization during PDPm-*Nal* stimulation in HeLa Kyoto cells.** Images presented in Fig. 6c are based on this movie. Cells were treated after frame 3 (45 s) by adding 100 µL of starvation media with 150 µM PDPm-*Nal* resulting in a final concentration of 50 µM.

File name: Supplementary Movie 4

Description: **Live-cell imaging of mKate2-GAB2 localization during EGF stimulation in Caco-2 BBe1 cells.** Images presented in Supplementary Figure 8a are based on this movie. As described in the corresponding figure legend, time-lapse-experiments consisted of an image acquisition interval of 15 s for at least 12 min for each sample condition separately using an exposure time of 0.5 frames per second. Cells were treated after frame 3 (45 s) by adding 100 µL of starvation media with 300 ng/mL EGF resulting in a final concentration of 100 ng/mL.

File name: Supplementary Movie 5

Description: **Live-cell imaging of mKate2-GAB2 localization during PDP-*Nal* stimulation in Caco-2 BBe1 cells.** Images presented in Supplementary Figure 8a are based on this movie. Cells were treated after frame 3 (45 s) by adding 100 µL of starvation media with 150 µM PDP-*Nal* resulting in a final concentration of 50 µM.

File name: Supplementary Movie 6

Description: **Live-cell imaging of mKate2-GAB2 localization during PDPm-*Nal* stimulation in Caco-2 BBe1 cells.** Images presented in Supplementary Figure 8a are based on this movie. Cells were treated after frame 3 (45 s) by adding 100 µL of starvation media with 150 µM PDPm-*Nal* resulting in a final concentration of 50 µM.

File name: Supplementary Movie 7

Description: **Live-cell imaging of mKate2-GAB2 localization during EGF stimulation in SW-480 cells.** Images presented in Supplementary Figure 8b are based on this movie. As described in the corresponding figure legend, time-lapse-experiments consisted of an image acquisition interval of 15 s for at least 12 min for each sample condition separately using an exposure time of 0.5 frames per second. Cells were treated after frame 3 (45 s) by adding 100 µL of starvation media with 300 ng/mL EGF resulting in a final concentration of 100 ng/mL.

File name: Supplementary Movie 8

Description: **Live-cell imaging of mKate2-GAB2 localization during PDP-*Nal* stimulation in SW-480 cells.** Images presented in Supplementary Figure 8b are based on this movie. Cells were treated after frame 3 (45 s) by adding 100 µL of starvation media with 150 µM PDP-*Nal* resulting in a final concentration of 50 µM.

File name: Supplementary Movie 9

Description: **Live-cell imaging of mKate2-GAB2 localization during PDPm-*Nal* stimulation in SW-480 cells.** Images presented in Supplementary Figure 8b are based on this movie. Cells were treated after frame 3 (45 s) by adding 100 µL of starvation media with 150 µM PDPm-*Nal* resulting in a final concentration of 50 µM.

**SUPPLEMENTARY DATA LEGENDS**

File name: Supplementary Data 1

Description: **Supplementary Data 1. List of all measurements and their sample-specific correlation between Mascot Score and FDR in the PLDMS approach.** Cut-off at an FDR <0.05 (5%) is highlighted in bold letters and is underlined.

File name: Supplementary Data 2

Description: **Supplementary Data 2. Results of quantitative phosphoproteomics measurements.** This table includes all output data on replicates and phosphorylation site identification and was the sole basis for all follow-up analysis of the 3,342 identified phosphorylation sites. MaxQuant results were imported into the software suite Perseus (v.1.5.8.5). Phosphorylation sites were filtered for at least 3 valid values for at least one experimental group. TMT batch effects were reduced by row-wise normalization based on median intensities. One-way ANOVA multiple sample test was performed applying permutation-based FDR (cutoff: 1%, 250 randomizations). Missing values were imputed from the normal distribution (width 0.3, downshift 1.8). The table also includes information for annotation of phosphorylation sites in the PhosphositePlus database (v03.07.18).

For pair-wise comparisons between conditions a two-sided unpaired Student’s t-test was performed to assess the statistical significance of dephosphorylation events, as judged by changes in log2 TMT reporter intensities. Protein p-values were corrected for multiple testing using a permutation based 5 % FDR cut-off (250 randomizations).

Hierarchical clustering for grouping of phosphorylation sites with similar behavior upon PP1c/PP2Ac treatment was performed on z-scored median log2 transformed reporter intensities of significant proteins after one-way ANOVA as described above. Clusters 2910 and 2911 were identified to contain phosphorylation sites selectively dephosphorylated by PP1c (777 sites) and PP2Ac (663 sites), respectively. Cluster 2904 contained 1361 phosphorylation sites that were decreased upon treatment with both phosphatases (see also Fig. 5b). Other significantly smaller clusters obtained from this hierarchical clustering analysis included p-sites that showed unexpected behavior (e.g. increased phosphorylation upon PP1c/PP2Ac treatment, non-reproducible behavior). These clusters are still included in the table shown here and are also depicted in Figure 5b. Their behavior might be caused by indirect signaling events, such as kinase activation upon kinase dephosphorylation by PP1c/PP2Ac but were not investigated further.

File name: Supplementary Data 3

Description: **Supplementary Data 3. MS analysis of the 14-3-3 interactome upon PP1c treatment to identify PP1-sensitive 14-3-3 binding proteins.** MaxQuant results were imported into the associated software suite Perseus (v.1.5.8.5). Label-free quantification (LFQ) intensities were filtered for at least 3 valid values for at least one experimental group. Missing values were imputed from the normal distribution (width 0.3, downshift 1.8). For the pairwise comparisons GFP/untreated and untreated/PP1c-treated samples, a two-sided unpaired Student’s t-test based on LFQ intensities was performed to assess the statistical significance. Protein p-values were corrected for multiple testing using a permutation based 5 % FDR cut-off (250 randomizations). If a protein was significantly more associated to GFP-14-3-3 compared to GFP alone (comparing GFP vs. untreated) it was considered a 14-3-3 binding protein (see Supplementary Fig. 9b). Proteins significantly dissociating from 14-3-3 proteins upon PP1c treatment (comparison untreated/PP1c-treated) were considered to be PP1-sensitive 14-3-3 binding proteins. If a protein was significantly enriched in untreated GFP-14-3-3 samples compared to GFP control samples and at the same time significantly decreased upon PP1c treatment, it was considered a high-confidence PP1-sensitive 14-3-3 binding protein. In total, the Perseus analysis identified 708 proteins, of which 108 were significantly enriched in GFP-14-3-3 compared to GFP control samples, and within those 108 proteins were 56 proteins, which significantly dissociated from GFP-14-3-3 upon PP1 treatment.

Proteins significantly dissociating from 14-3-3 upon PP1 treatment were mapped back onto the phosphoproteomic dataset and linked to 1,967 class I phosphorylation sites dephosphorylated upon PP1c-treatment (two-sided t-test untreated vs. PP1-treated, see also Supplementary Figure 4b and Supplementary Data 2). This analysis resulted in an overlap of 87 phosphorylation sites on 35 out of 56 PP1-sensitive 14-3-3 binding proteins that do not bind GFP alone (see also Fig. 7c).
