# Supplementary material for: Dissecting the sequence determinants for dephosphorylation by the catalytic subunits of phosphatases PP1 and PP2A
Source: Nat Commun. 2020 Jul 17;11:3583. doi: 10.1038/s41467-020-17334-x (PMC7367873; doi:10.1038/s41467-020-17334-x)
Supplement: Supplementary file 16 — Reporting Summary [file 41467_2020_17334_MOESM16_ESM.pdf]

nature research

Corresponding author(s): Maja Köhn

Last updated by author(s): Jun 16, 2020

Reporting Summary

Nature Research wishes to improve the reproducibility of the work that we publish. This form provides structure for consistency and transparency in reporting. For further information on Nature Research policies, see Authors & Referees and the Editorial Policy Checklist .

Statistics

For all statistical analyses, confirm that the following items are present in the figure legend, table legend, main text, or Methods section.

n/a	Confirmed	
	x	The exact sample size (*n*) for each experimental group/condition, given as a discrete number and unit of measurement
	x	A statement on whether measurements were taken from distinct samples or whether the same sample was measured repeatedly
	x	The statistical test(s) used AND whether they are one- or two-sided<br>*Only common tests should be described solely by name; describe more complex techniques in the Methods section.*
x		A description of all covariates tested
	x	A description of any assumptions or corrections, such as tests of normality and adjustment for multiple comparisons
	x	A full description of the statistical parameters including central tendency (e.g. means) or other basic estimates (e.g. regression coefficient) AND variation (e.g. standard deviation) or associated estimates of uncertainty (e.g. confidence intervals)
	x	For null hypothesis testing, the test statistic (e.g. *F*, *t*, *r*) with confidence intervals, effect sizes, degrees of freedom and *P* value noted<br>*Give P values as exact values whenever suitable.*
x		For Bayesian analysis, information on the choice of priors and Markov chain Monte Carlo settings
x		For hierarchical and complex designs, identification of the appropriate level for tests and full reporting of outcomes
x		Estimates of effect sizes (e.g. Cohen's *d*, Pearson's *r*), indicating how they were calculated

Our web collection on statistics for biologists contains articles on many of the points above.

Software and code

Policy information about availability of computer code

Data collection	Mass spectrometry measurements have been performed on Q Exactive Plus Orbitrap or Orbitrap Fusion Lumos mass spectrometers (Thermo Fischer Scientific) and commercial operating software from Thermo Fischer Scientific was used. Gel-filtration was performed on an ÄKTA Explorer system and the commercial software Unicorn v5 was used. Live-cell imaging was performed on a A1R Confocal Scanning System equipped on a Nikon Ti-E inverted microscope using the commercial operating software NIS Elements 4.5. Western blots were imaged on a ChemiDoc Touch Imaging System (BioRad) or a Fusion FX Imaging System (Vilber). Biochemical assays were read on a Synergy H4 microplate reader (Biotek) using the commerical Gen5 software.
Data analysis	Mascot 2.4 (Matrix Science) was used for peptide searching, isobarQuant (https://github.com/protcode/isob/archive/1.1.0.zip) and MaxQuant (v1.6.0.16) were used to quantify peptide and protein abundances, R (v3.3.2, v3.5.1) and Perseus (v.1.5.8.5) were used for custom data analysis and statistical analysis. Image analysis was carried out in Fiji (Fiji is Just Image J) v2.0.0-rc-34/1.50a and Western blots were quantified in ImageLab (BioRad) v6.0.1. Protein structures were inspected in PyMOL 2.3.3. Biochemical experiments and kinetics were analyzed and visualized in GraphPad Prism v6.

For manuscripts utilizing custom algorithms or software that are central to the research but not yet described in published literature, software must be made available to editors/reviewers. We strongly encourage code deposition in a community repository (e.g. GitHub). See the Nature Research guidelines for submitting code & software for further information.

October 2018

Data

Policy information about availability of data

All manuscripts must include a data availability statement. This statement should provide the following information, where applicable:

- Accession codes, unique identifiers, or web links for publicly available datasets
- A list of figures that have associated raw data
- A description of any restrictions on data availability

Mass spectrometry data have been deposited at the ProteomeXchange Consortium (proteomecentral.proteomexchange.org) using the PRIDE partner repository with the identifiers PXD012026 and PXD013775. The output of all MS-based results is furthermore summarized in Supplementary Tables 1-3 and Supplementary Data 1-3. Supplementary Tables 1-3 as well as Supplementary Figures 1-10 are found in the Supplementary Information, Supplementary Data 1-3 are provided as separate excel files. The source data underlying Figures 1c,d; 2a-e, 3b,d; 4a,b; 5a; 6a,b,e and Supplementary Figures 1a-c; 2a-e; 3a-c; 5, 6, 7a,b and 8a,b, data for quality control of directed peptide synthesis and certificates for cell line authentication are provided as a Source Data. MS reference datasets were downloaded from Swiss-Prot/UniprotKB (www.uniprot.org). Protein structures were downloaded from www.rcsb.org/pdb.org using identifiers 2NPP, 3EGG, 4I5L, 3DW8. Phosphorylation site annotation was downloaded from www.phosphosite.org (accessed 3 July 2018) and datasets for phosphatase regulators were obtained from genenames.org and uniprot.org (accessed March/April 2020). All other data supporting the findings presented herein are available from the authors upon request.

Field-specific reporting

Please select the one below that is the best fit for your research. If you are not sure, read the appropriate sections before making your selection.

- [x] Life sciences
- [ ] Behavioural & social sciences
- [ ] Ecological, evolutionary & environmental sciences

For a reference copy of the document with all sections, see nature.com/documents/nr-reporting-summary-flat.pdf

Life sciences study design

All studies must disclose on these points even when the disclosure is negative.

Sample size	Biological triplicates were chosen for sample size. These are standard in basic life science research and have led to conclusive results with appropriate statistical power in published MS studies and molecular biology experiments using similar approaches in the past.
Data exclusions	Raw data of MS experiments deposited to the PRIDE repository contains additional channels not presented as a whole in the manuscript since they did not fulfill quality criteria . They were not used for findings presented in the manuscript and their exclusion was pre-established before proceeding with biological interpretations.
Replication	All findings presented in the manuscript are based at least on three successfully replicated attempts. All replicated attempts under the same conditions were successful. For Supplementary Figures 5 and 6, gel-filtration was only performed once per condition, since the elution volume of proteins/complexes in gel-filtration is highly reproducible and like HPLC analysis of chemicals a single injection is standard.
Randomization	Randomization does not apply to our manuscript, as the study is based on replications from a single cell line without additional covariants normally found in studies carried out in animals/humans.
Blinding	Since no covariants are present in our study, all data could be analyzed by unbiased statistics. Blinding therefore is not relevant to the setup presented in the manuscript.

Reporting for specific materials, systems and methods

We require information from authors about some types of materials, experimental systems and methods used in many studies. Here, indicate whether each material, system or method listed is relevant to your study. If you are not sure if a list item applies to your research, read the appropriate section before selecting a response.

Materials & experimental systems

n/a	Involved in the study	
☐	☒	Antibodies
☐	☒	Eukaryotic cell lines
☒	☐	Palaeontology
☒	☐	Animals and other organisms
☒	☐	Human research participants
☒	☐	Clinical data

Methods

n/a	Involved in the study	
☒	☐	ChIP-seq
☒	☐	Flow cytometry
☒	☐	MRI-based neuroimaging

Antibodies

Antibodies used	Primary antibodies: GFP (Abcam, ab6556, polyclonal, Lots GR3216572-1, GR292567-1), GAB2 (CST, #3239, clone 26B6, Lot 3), GAB2-pS210 (Symansis, customized purification, 3043-P1, Lot 1708.01), FLAG M2 (Sigma-Aldrich, F3165, Lots SLBT6752, SLBH1191V), pan14-3-3 (SCBT, sc-629, polyclonal, K-19, Lot C3115), PP1 (SCBT, sc-7482, E-9, Lots I0413, B2715), PP2A (SCBT, sc-130237, N-25, Lot I1109) or 6His (Abcam, ab9108, Lot GR3247563-6)<br>Secondary antibodies: goat anti-mouse IgG-peroxidase conjugate (Sigma, A0168, Lot 079M4881V), donkey anti-rabbit IgG-horseradish peroxidase (HRP) conjugate (GE Healthcare, GENA934, Lot 16964883), rabbit anti-sheep IgG (H+L) HRP conjugate (Thermo Fisher Scientific, 61-8620, Lot RI242148)
Validation	GFP (Abcam, ab6556): one of the best verified anti-GFP antibodies on the market. Verified for multiple applications by the vendor and in our laboratory by Western blotting. >700 references<br>GAB2 (CST, #3239): verified in our laboratory by overexpression of Flag- and mKate2-Gab2 and subsequent Western blotting. >10 citations on the vendors website and on the Validated Antibody Database (VAD, labome.org)<br>GAB2-pS210 (Symansis, customized purification): This antibody was purified by Symansis after recommendation by T. Brummer. Originally, this antibody was produced for T. Brummer in his study published in: Brummer et al., EMBO J., 2008 (PMID 19172738). Phosphospecific binding of the antibody is also demonstrated by the phosphatase inhibition presented in this manuscript by Western blotting.<br>FLAG M2 (Sigma-Aldrich, F3165): one of the best verified anti-FLAG antibodies with >3000 references on the vendors website and >160 references in the Validated Antibody Database (VAD, labome.org). Verified multiple times in our laboratory by the comparison of Flag-transfected cell lines to untransfected controls by Western blotting and IF.<br>pan14-3-3 (SCBT, sc-629): verified multiple times by transfection of eGFP-14-3-3beta and Flag-14-3-3beta. Compared to GFP, Flag signals and the signal of a monoclonal alternative (sc133233, clone B-8) from mouse by Western blotting. Used in >150 publications.<br>PP1 (SCBT, sc-7482): verified in our laboratory by multiple transfections of tagged PP1 protein as well as siRNA directed against isoforms alpha-gamma of PP1 in mammalian cells and subsequent Western blotting. Verified in the course of overexpression of His-tagged PP1 recombinantly expressed in bacteria and purification of recombinant protein and subsequent Western blotting. Used in >150 publications.<br>PP2A (SCBT, sc-130237): verified by usage in the same applications as described for PP1.<br>6His (Abcam, ab9108): >80 referencing publications, verified for cross-reactivity in mammalian cell lysate by immunoblotting of untransfected control samples.

Eukaryotic cell lines

Policy information about cell lines

Cell line source(s)	HeLa Kyoto cells were obtained from the BIOSS cell line repository (University of Freiburg), Caco-2 BBe1 cells from ATCC (ATCC CRL-2102), and SW-480 cells from the European Collection of Authenticated Cell Cultures (ECACC 87092801).
Authentication	Authenticated by PCR analysis of short tandem repeats (STRs) by an external, independent service provider (Labor f. DNA-Analytik, Freiburg, Germany). The obtained Pm-values (likelihood of random match) were 4.26e-15 (HeLa Kyoto), 5.86e-11 (Caco-2 Bbe1) and 3.84e-12 (SW480).
Mycoplasma contamination	All cell lines included in the study have been confirmed to be free of mycoplasma. Tests were carried out by an external, independent service provider (GATC/Eurofins, Ebersberg, Germany).
Commonly misidentified lines<br>(See ICLAC register)	No misidentified cell lines were used in this study.
