# Supplementary material for: Dissecting the sequence determinants for dephosphorylation by the catalytic subunits of phosphatases PP1 and PP2A
Source: Nat Commun. 2020 Jul 17;11:3583. doi: 10.1038/s41467-020-17334-x (PMC7367873; doi:10.1038/s41467-020-17334-x)

LABOR FÜR
DNA-ANALYTIK

PD Dr. Juliane Alt-Mörbe

Klarastraße 66 · 79106 Freiburg · Telefon + Fax 0761/40 79 56
e-mail: alt-moerbe@web.de · www.dna-analytik.de

DNA-Profil der Zell-Linie HeLa „Kyoto“ vom 12. 2. 20

Vorgehensweise:
Aus den Zellen wurde mit dem Nucleospin Tissue Kit von Machery-Nagel die Gesamt-DNA isoliert und diese dann in die PCR mit Primern für STR(“short tandem repeat“)-Loci eingesetzt. Als Referenz zur Bestimmung der Allele dienten die Allel-Leitern der Firma Promega.
Die Ergebnisse wurden mit den bei ATCC hinterlegten Daten für HeLa-Zellen verglichen Und in der Tabelle zusammengefasst.

DNA-Profil der Zell-Linie HeLa „Kyoto“ vom 12. 2. 20

		Zell-Linie HeLa „Kyoto“	Zell-Linie HeLa soll (http://www.atcc.org)	Pm (Probability of match)
D5S818		11, 12	11, 12	0.24
D13S317		12, 13.3	**12**, 13.3*	0.000056*
D7S820		8, 12	8, 12	0.05
D16S539		9, 10	9, 10	0.018
VWA		16, 18	16, 18	0.088
FGA**		18, 21	18, 21	0.0047
TPOX		8, 12	8, 12	0.004
D8S1179**		12, 13	12, 13	0.09
CSFPO		9, 10	9, 10	0.02
TH01		7	7	0.34
D18S51**		16	16	0.039
Amelogenin		X	X	0.5
Pm (probability of match)				4.26×10^{-15} (ohne D13S317) $2.39 \times ^{-19}$ (mit D13S317)

* Allel 13.3 im Locus D13S317 ist so extrem selten, dass ich in keiner Datenbank eine Angabe über die statistische Häufigkeit gefunden habe! Für die Berechnung wurde eine Häufigkeit von 0.00001 angenommen.
Um so sicherer ist es, dass die Zell-Linie o. k. ist.
** diese Loci erscheinen jetzt nicht mehr auf der Liste von ATCC, waren aber früher so hinterlegt.

Ergebnis: die Zellinie zeigt in allen 12 getesteten Loci das richtige Profil und ist in Ordnung, der Pm-Wert (die Wahrscheinlichkeit einer zufälligen Übereinstimmung) ist $2.39 \times ^{-19}$ (mit D13S317).

20.2.20

J. Alt Mörbe

(Dr. Juliane Alt-Mörbe)

D5S818 und D13S317

D:\FM\A180220.ALF

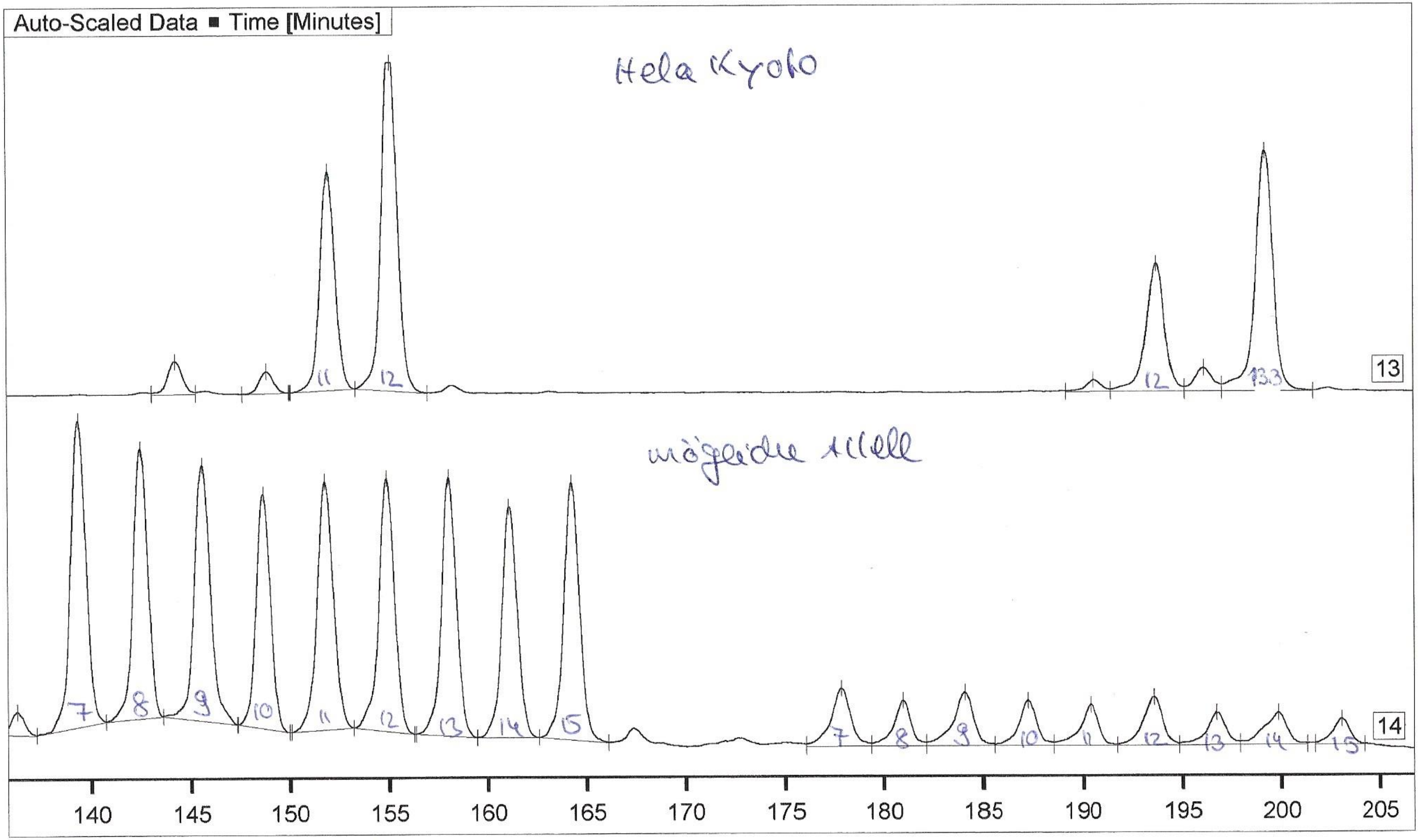

D5S818

D13S317

D 138317

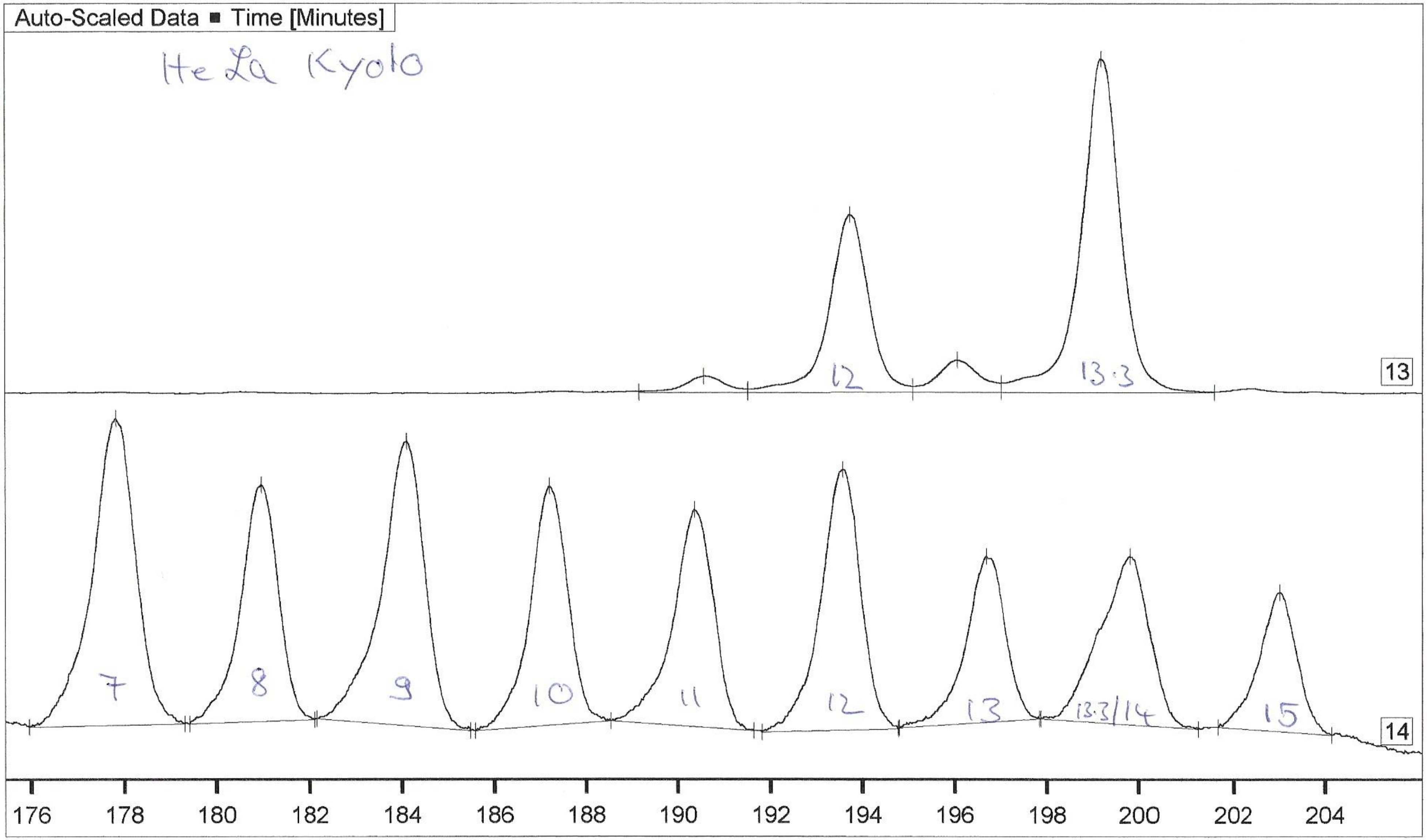
Auto-Scaled Data ■ Time [Minutes]
HeLa Kyoto
12
13.3
13
7
8
9
10
11
12
13
13.3/14
15
14
176
178
180
182
184
186
188
190
192
194
196
198
200
202
204

VWA und FGA

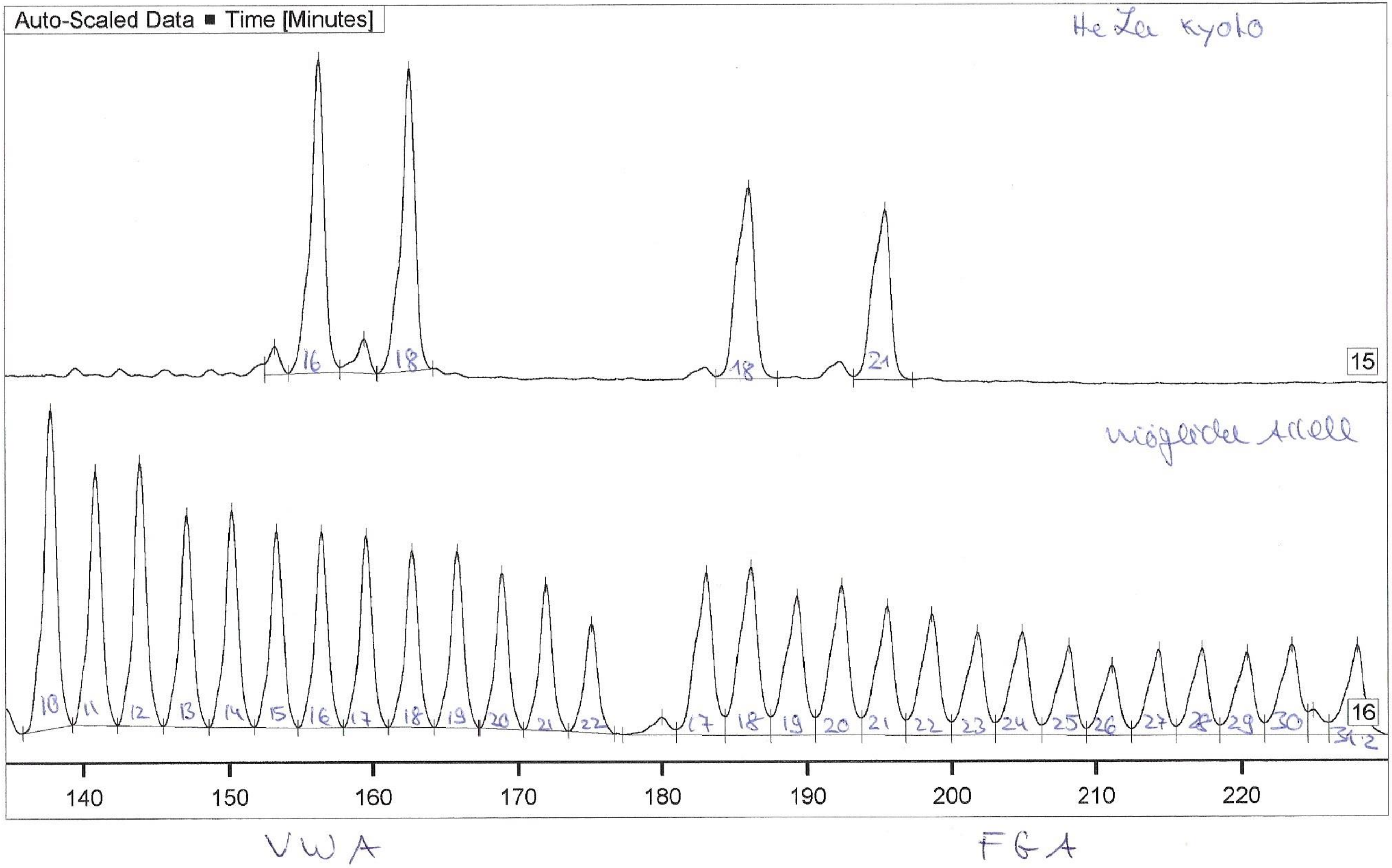
Auto-Scaled Data ■ Time [Minutes]
HeLa Kyoto
16
18
18
21
15
mögliche Allele
10
11
12
13
14
15
16
17
18
19
20
21
22
17
18
19
20
21
22
23
24
25
26
27
28
29
30
31.2
16
140
150
160
170
180
190
200
210
220
VWA
FGA

D7S820 und TPOX

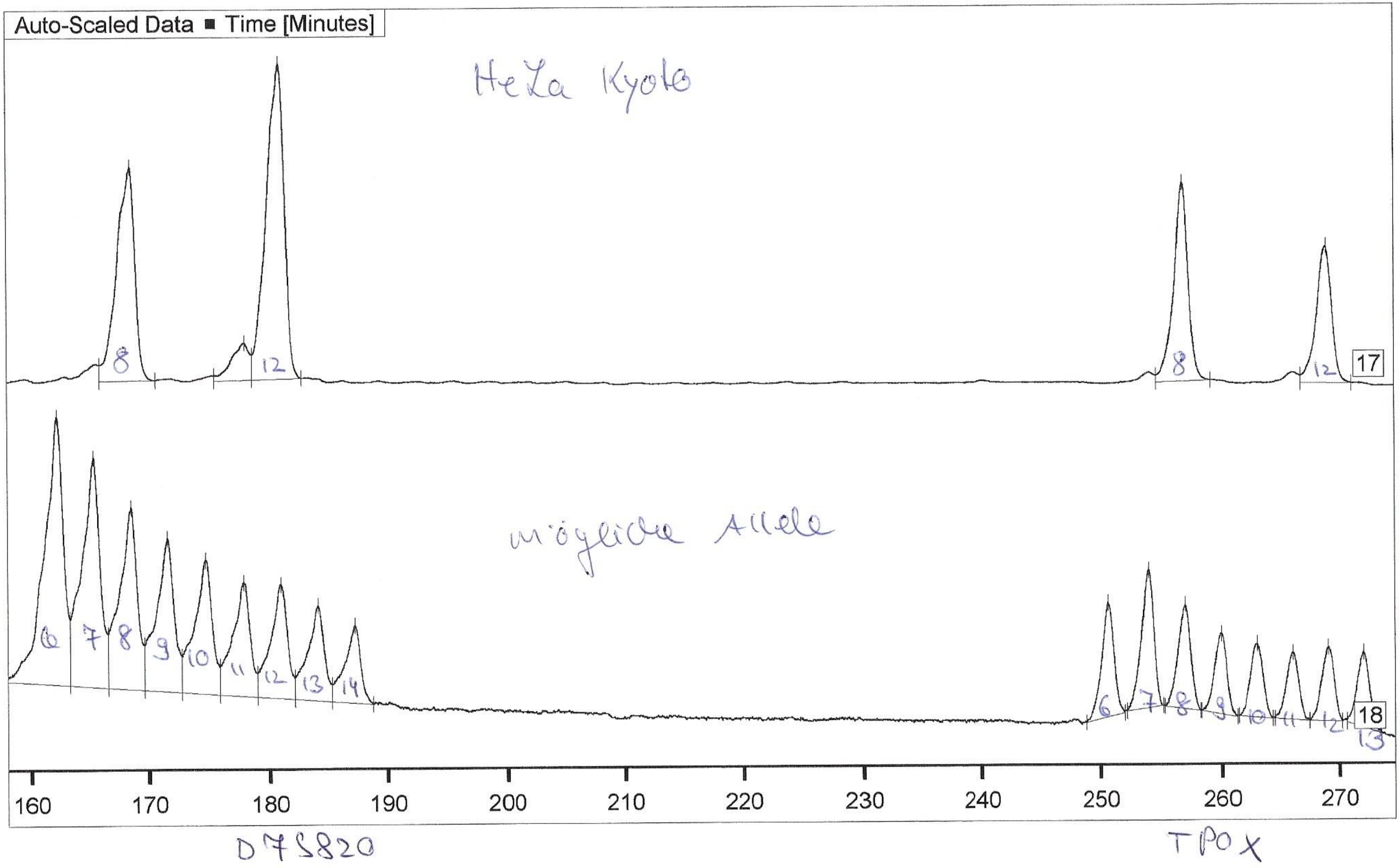

D16S539 und D8S1179

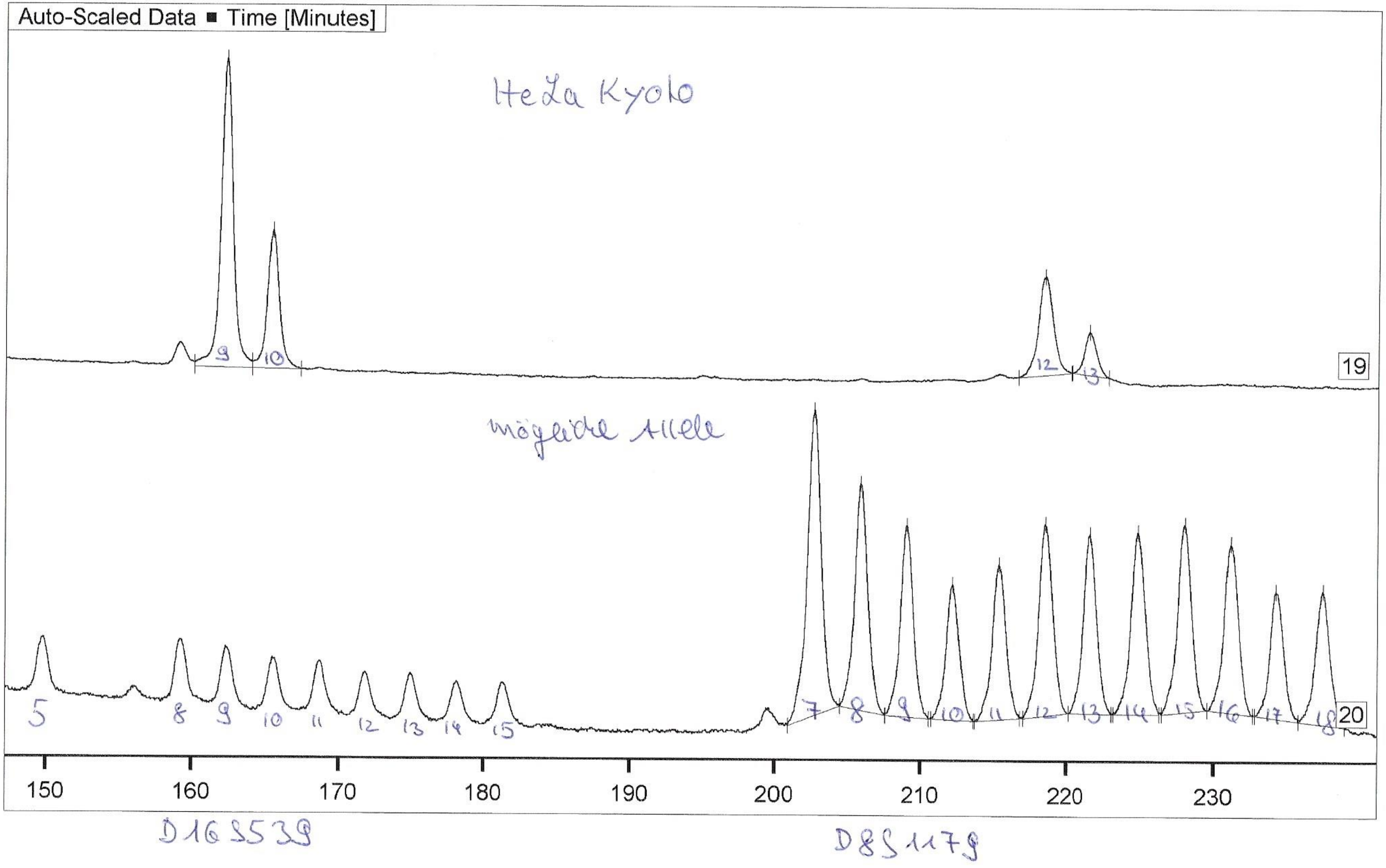

He La "Kyoto" CSFPO

18/Feb/120 23:05:20
D:\FM\A180220.ALF

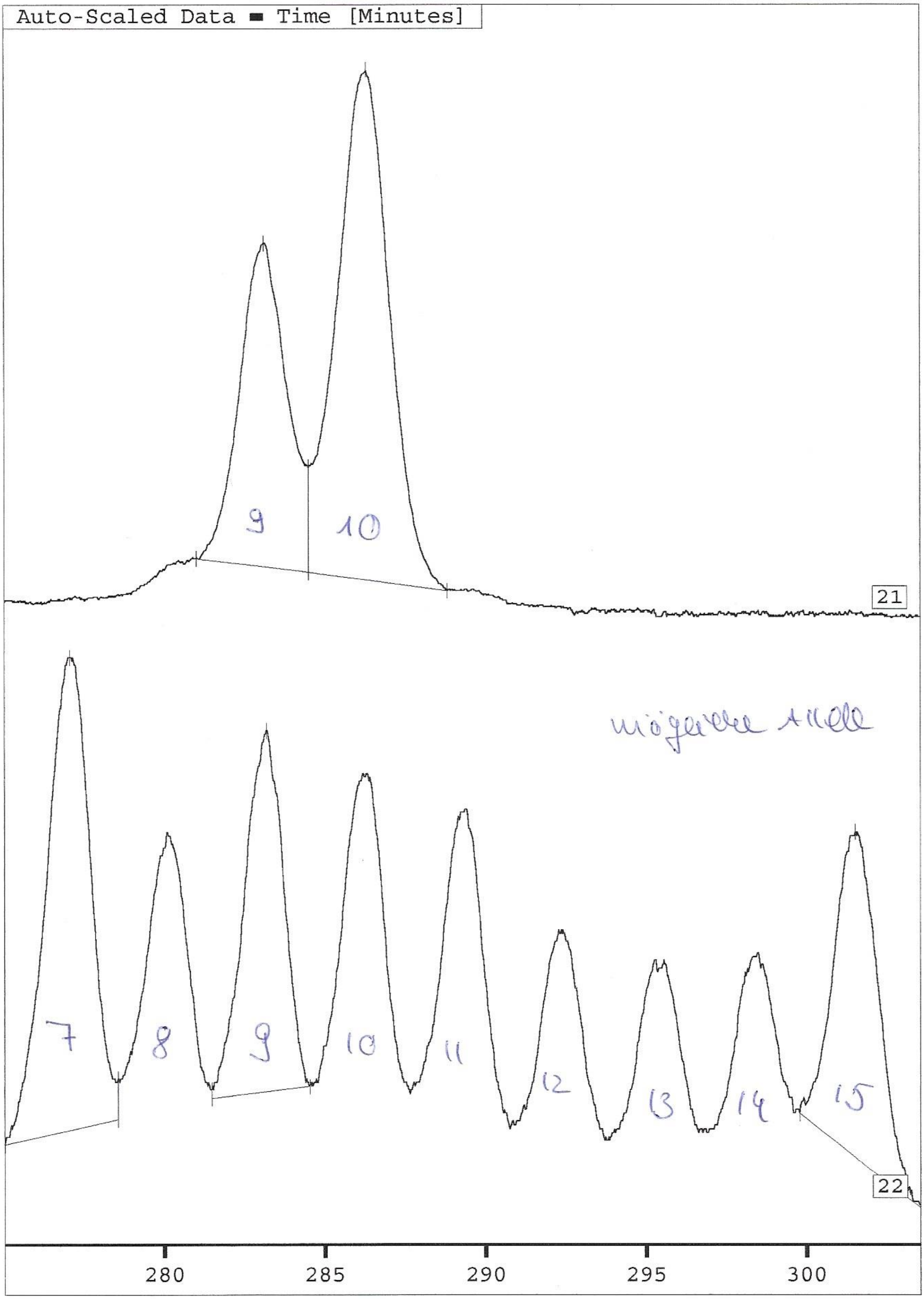

TH01

18/Feb/120 23:02:45
D:\FM\A180220.ALF

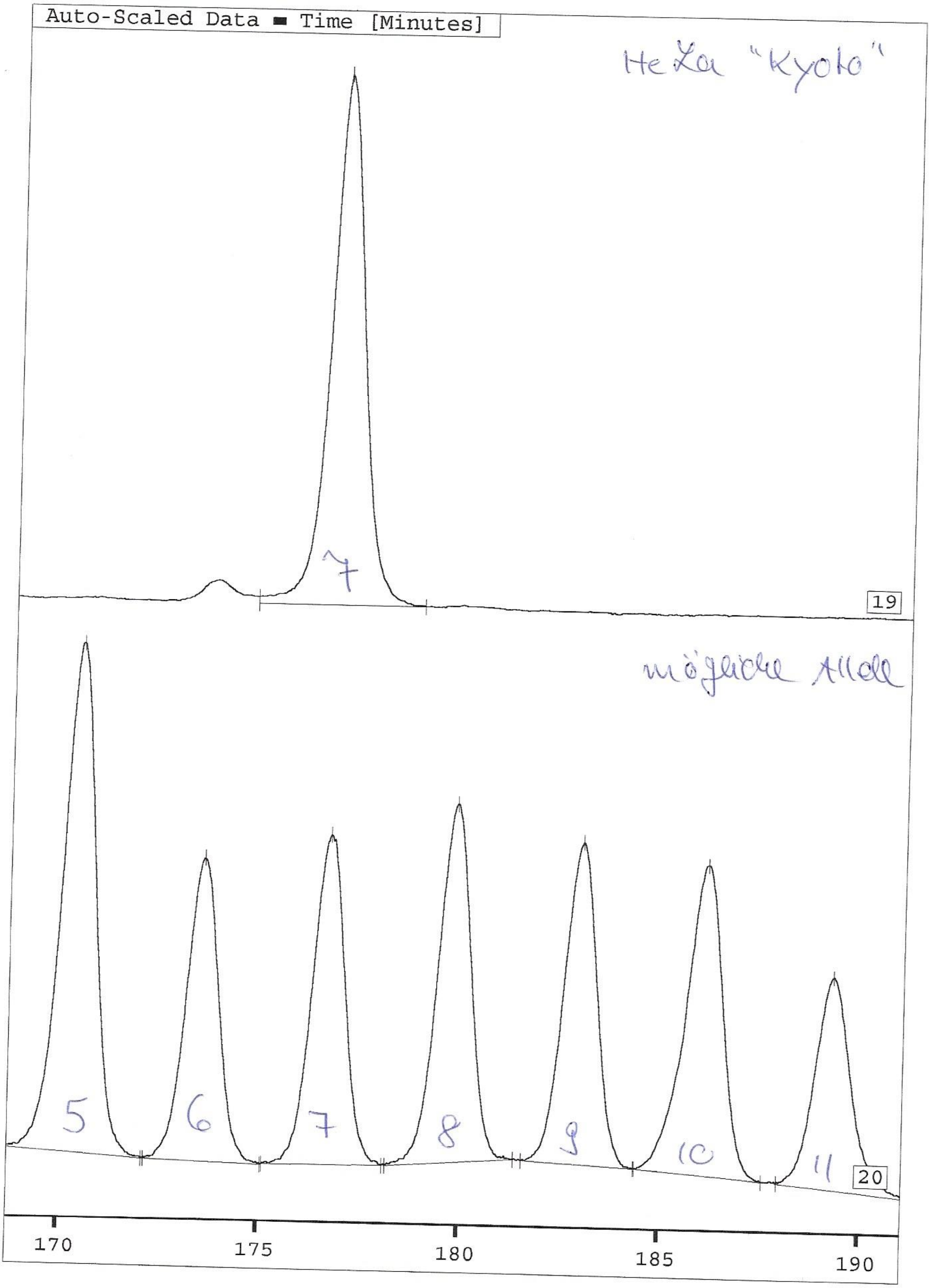

D18S51

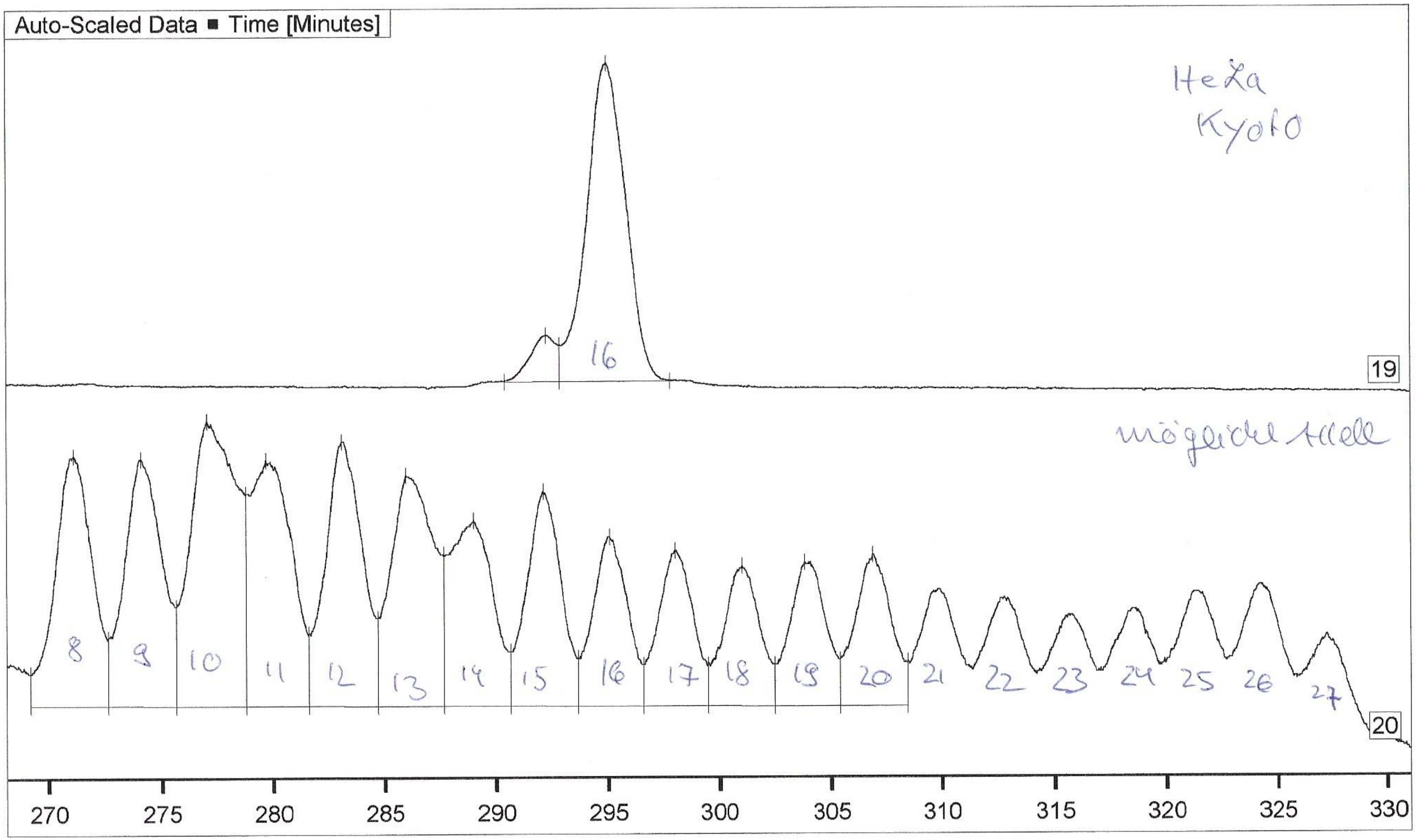
Auto-Scaled Data ■ Time [Minutes]
HeLa
Kyoto
16
19
mögliche Allele
8
9
10
11
12
13
14
15
16
17
18
19
20
21
22
23
24
25
26
27
20
270
275
280
285
290
295
300
305
310
315
320
325
330

Amelogenin

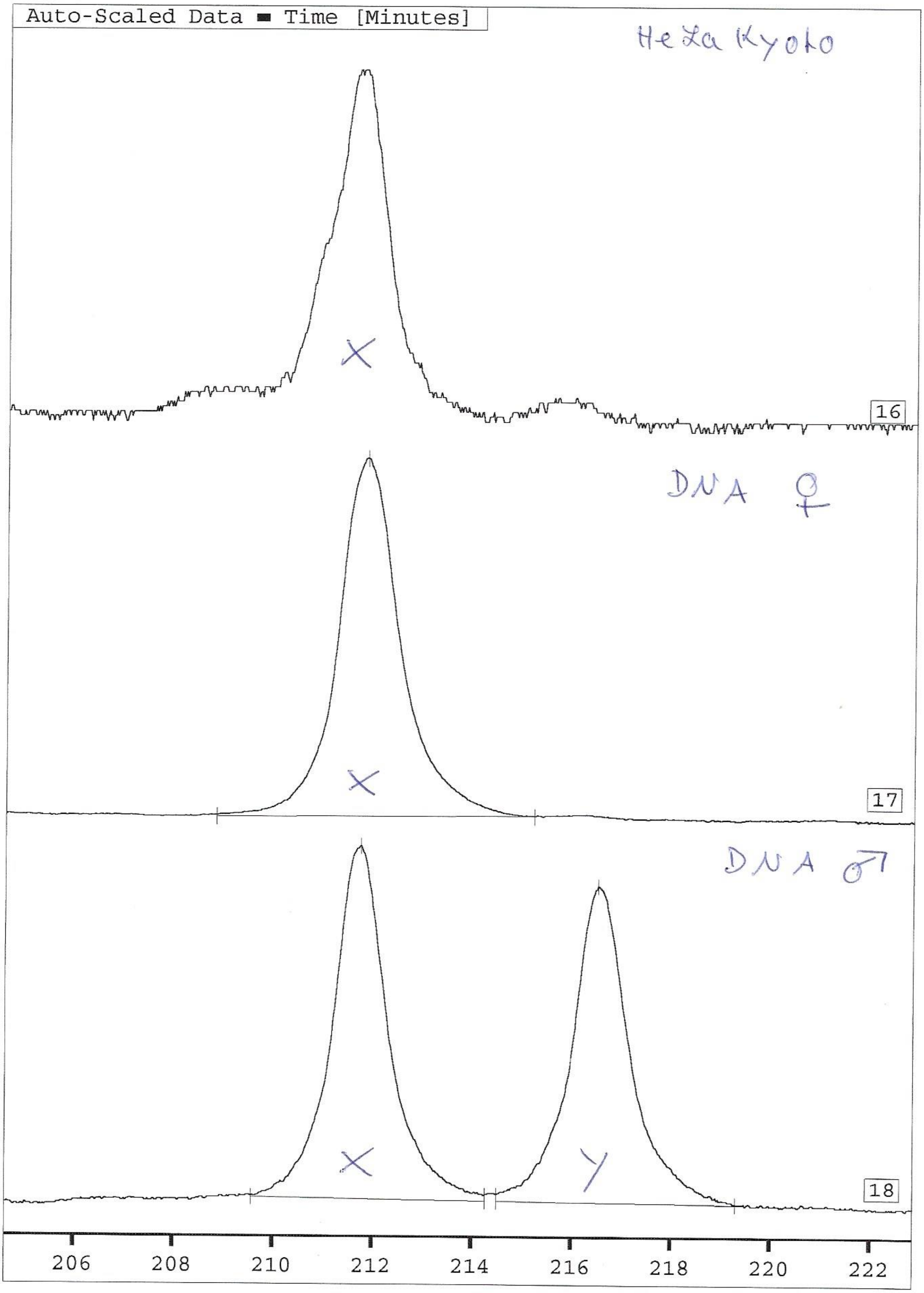
Auto-Scaled Data ■ Time [Minutes]
HeLa Kyoto
X
16
DNA ♀
X
17
DNA ♂
X
Y
18
206
208
210
212
214
216
218
220
222

LABOR FÜR
DNA-ANALYTIK

PD Dr. Juliane Alt-Mörbe

Klarastraße 66 · 79106 Freiburg · Telefon + Fax 0761/40 79 56
e-mail: alt-moerbe@web.de · www.dna-analytik.de

DNA-Profil der Zell-Linien Caco2 und SW480 vom 13. 03. 20

Vorgehensweise:
Aus den Zellen wurde mit dem Nucleospin Tissue Kit von Machery-Nagel die Gesamt-DNA isoliert und diese dann in die PCR mit Primern für STR("short tandem repeat")-Loci eingesetzt. Als Referenz zur Bestimmung der Allele dienten die Allel-Leitern der Firma Promega.
Die Ergebnisse wurden mit den bei ATCC hinterlegten Daten verglichen
Und in der Tabelle zusammengefasst.

DNA-Profil der Zell-Linien Caco2 und SW480 vom 13. 03. 20

	Zell-Linie Caco2	Zell-Linie Caco2 soll (http://www.atcc.org)	Pm (Probability of match)	Zell-Linie SW480	Zell-Linie SW480 soll (http://www.atcc.org)	
D5S818	12, 13	12, 13	0,124	13	13	0,057
D13S317	11, 13, 14	11, 13, 14	0,0026	12	12	0,153
D7S820	11, 12	11, 12	0,061	8	8	0,049
D16S539	12, 13	12, 13	0,088	13	13	0,047
VWA	16, 18	16, 18	0,088	16	16	0,084
TPOX	9, 11	9, 11	0,049	11	11	0,125
CSFPO	11	11	0,162	13, 14	13, 14	0,0014
TH01	6	6	0,097	8	8	0,026
Amelogenin	X	X	0,5	X	X	0,5
Pm (probability of match)			$5,86 \times 10^{-11}$			$3,84 \times 10^{-12}$

Ergebnis: beide Zellinien zeigen in allen neun getesteten Loci das richtige Profil und sind in Ordnung. Der Pm-Wert (die Wahrscheinlichkeit einer zufälligen Übereinstimmung) für die Zell-Linie Caco2 ist $5,86 \times 10^{-11}$, der Pm-Wert für die Zell-Linie SW480 $3,84 \times 10^{-12}$.

25. 03. 2020

J. Alt Mörbe

(Dr. Juliane Alt-Mörbe)

D5S818

A1

20/Mar/120 22:06:04
D:\FM\A170320.ALF

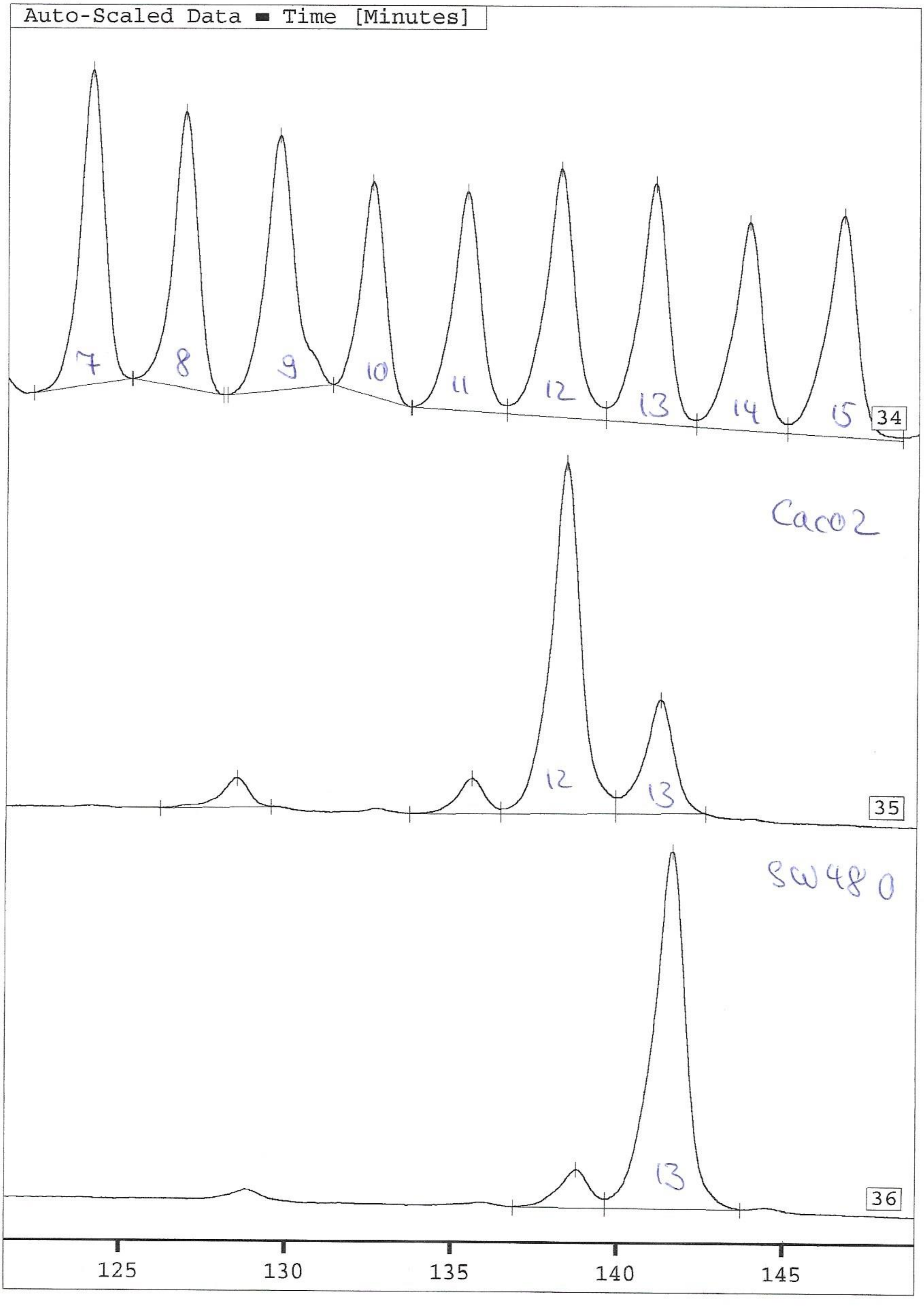

D13S317 A2

20/Mar/120 22:05:31
D:\FM\A170320.ALF

Auto-Scaled Data ■ Time [Minutes]

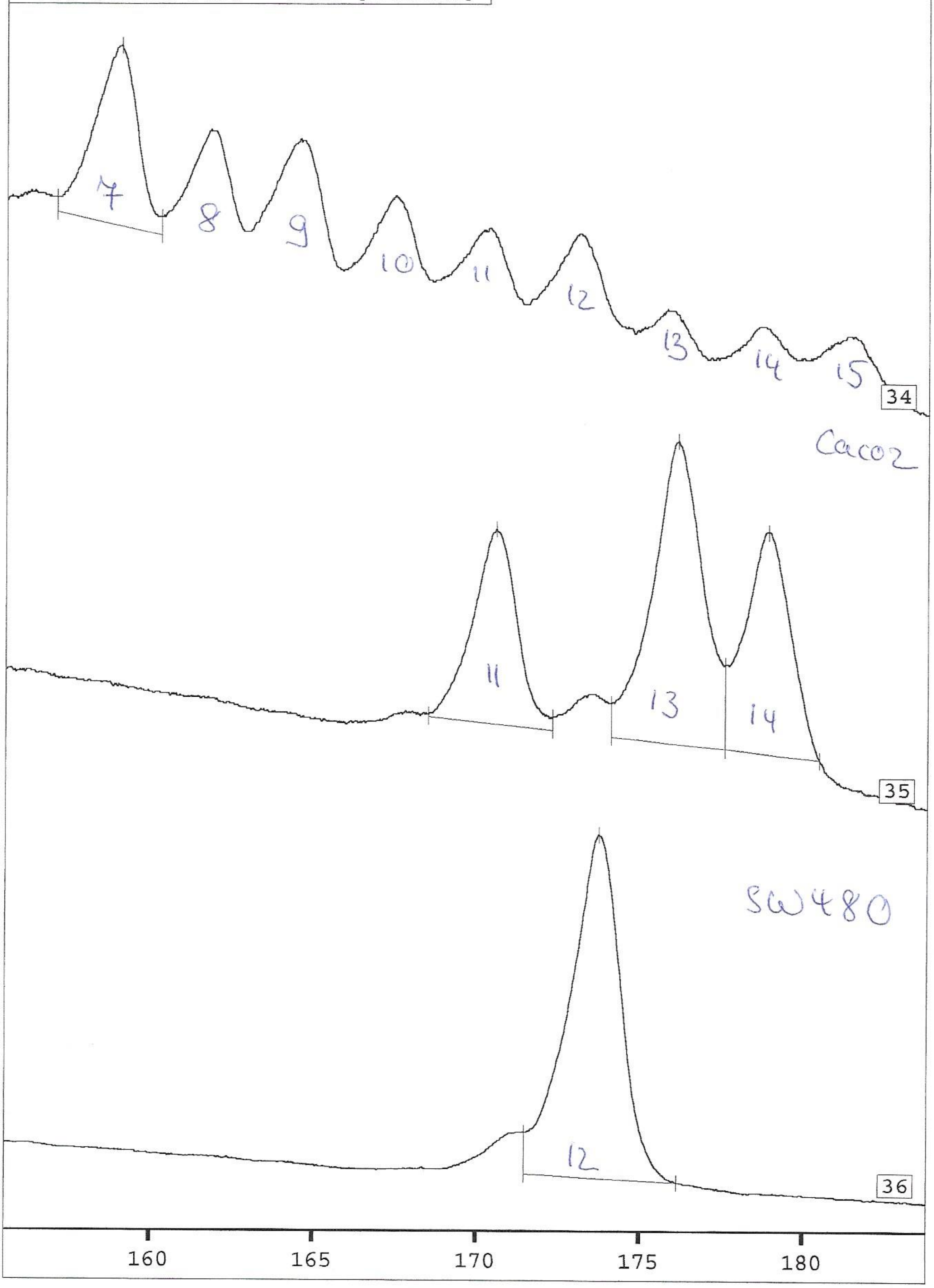

D7S820

A3

D:\FM\A190320.ALF

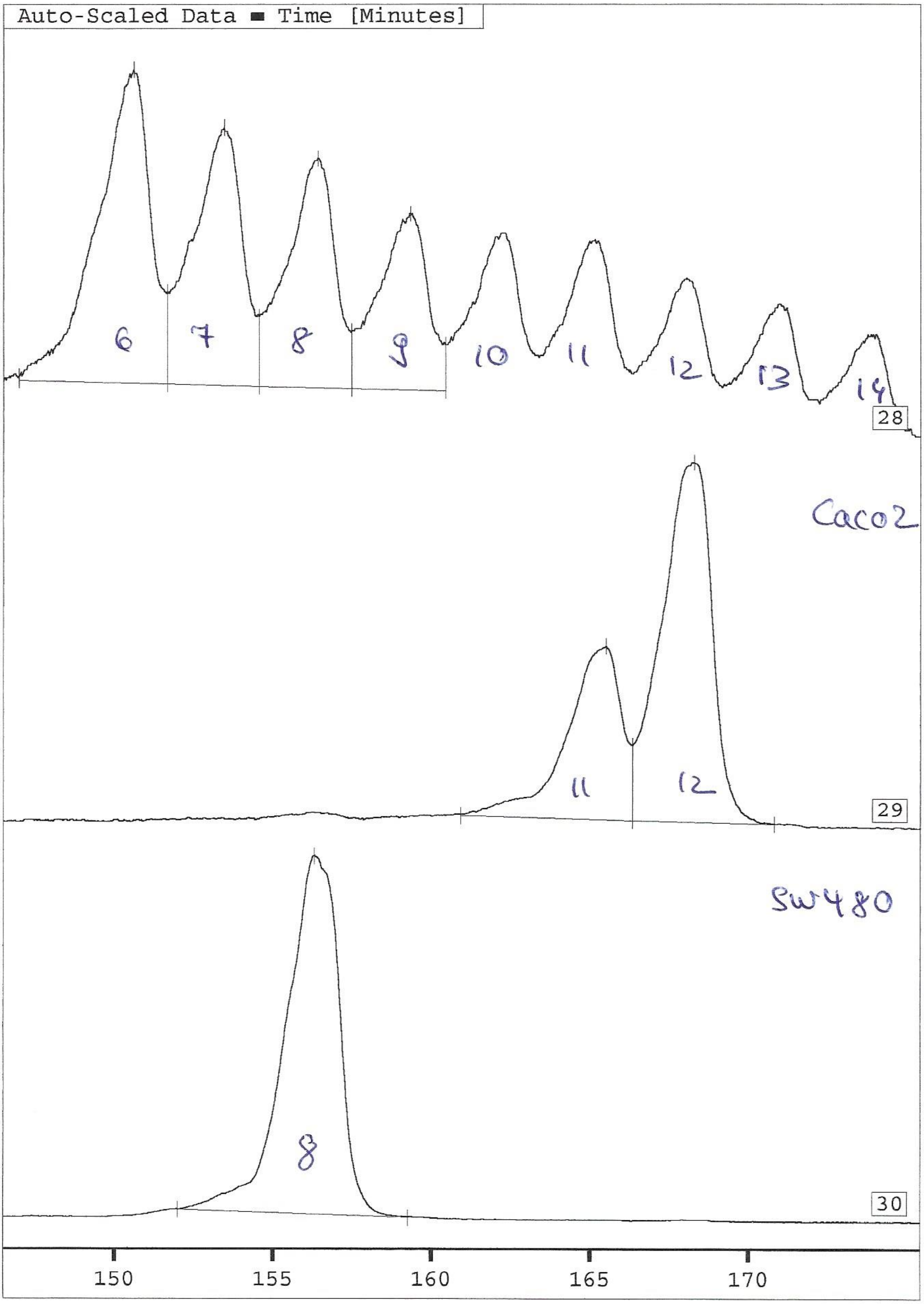

A4

D16S539 und D8S1179

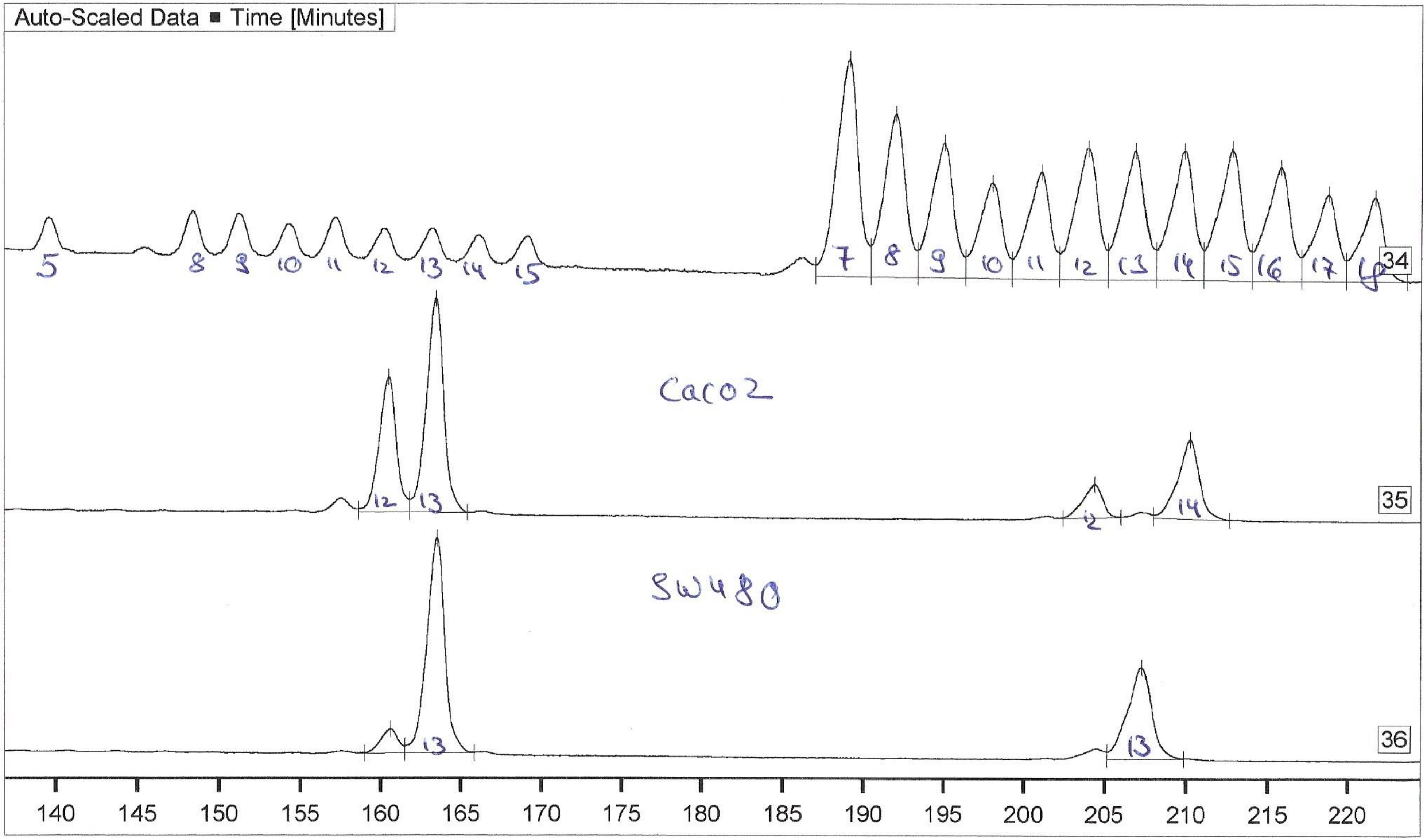

D16 S539

D8S1179

A5 VWA + (FGA)

19/Mar/120 21:50:47
D:\FM\A190320.ALF

Pharmacia DNA Fragment Manager V1.2

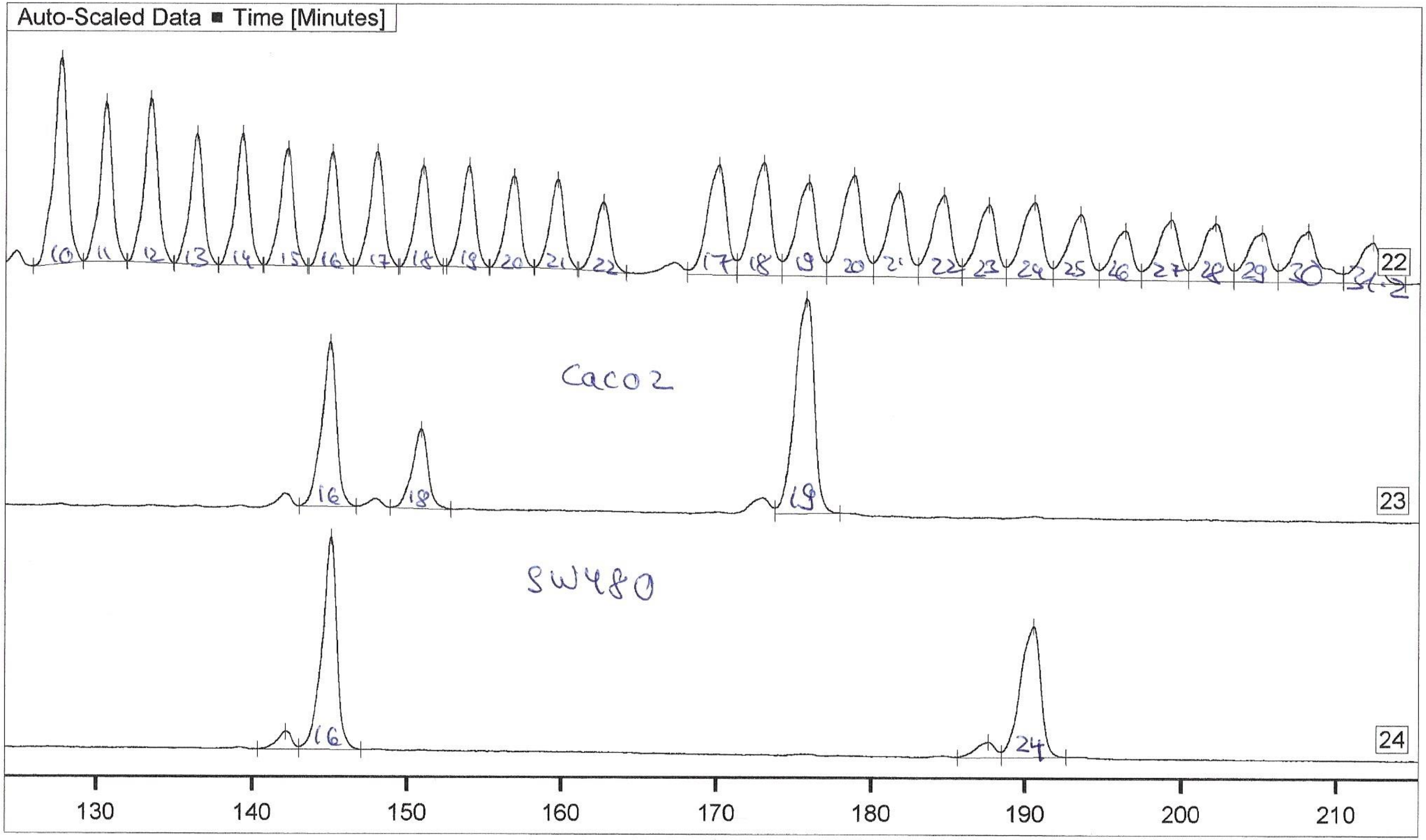

TPOX

A6

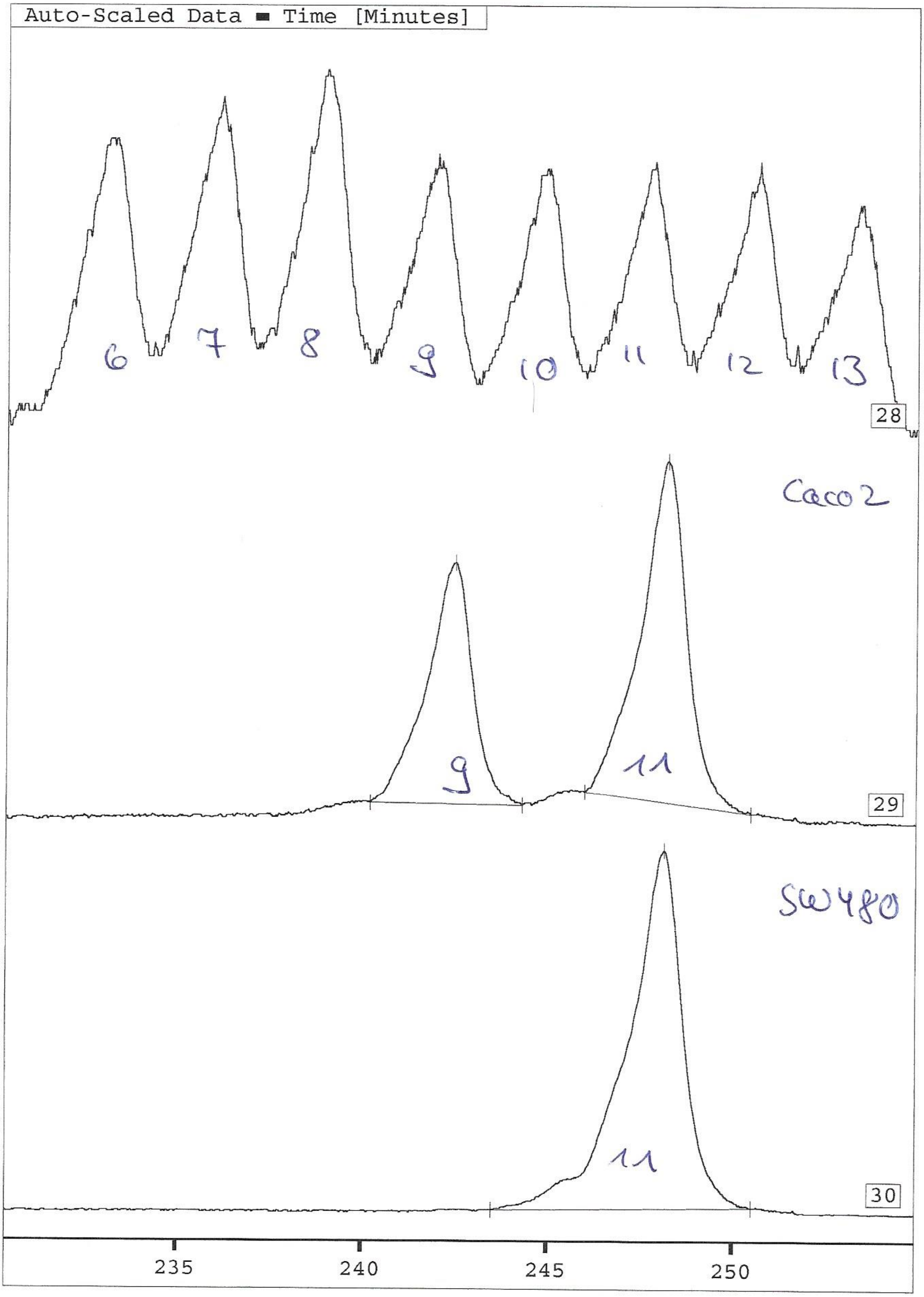
Auto-Scaled Data ■ Time [Minutes]
6
7
8
9
10
11
12
13
28
Caco2
9
11
29
SW480
11
30
235
240
245
250

CSFPO

A7

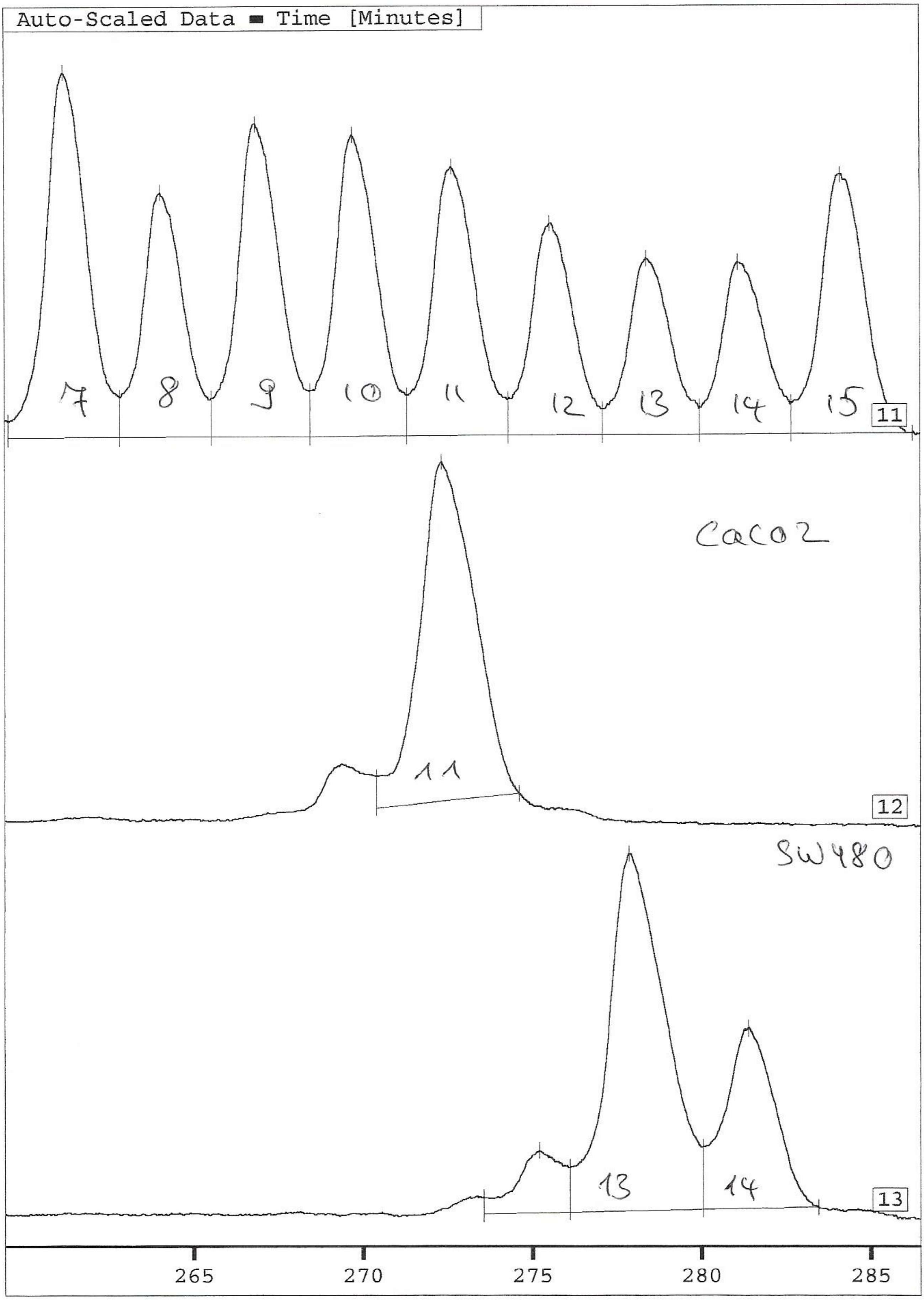
Auto-Scaled Data ■ Time [Minutes]
7
8
9
10
11
12
13
14
15
11
Caco2
11
12
SW480
13
14
13
265
270
275
280
285

THO1

A8

17/Mar/120 20:38:46
D:\FM\A170320.ALF

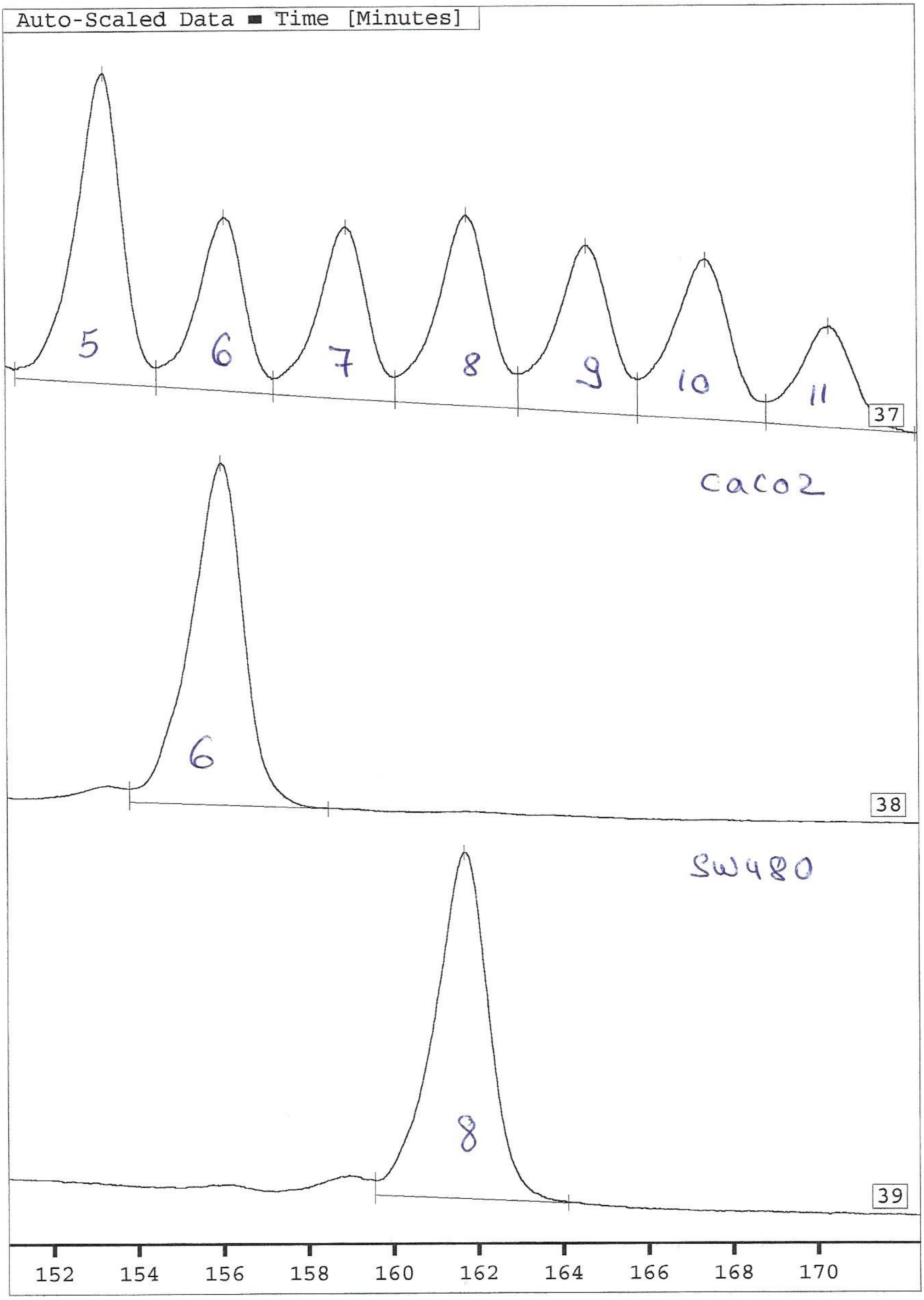

Amelogenin A9

24/Mar/120 16:37:10
D:\FM\A230320.ALF

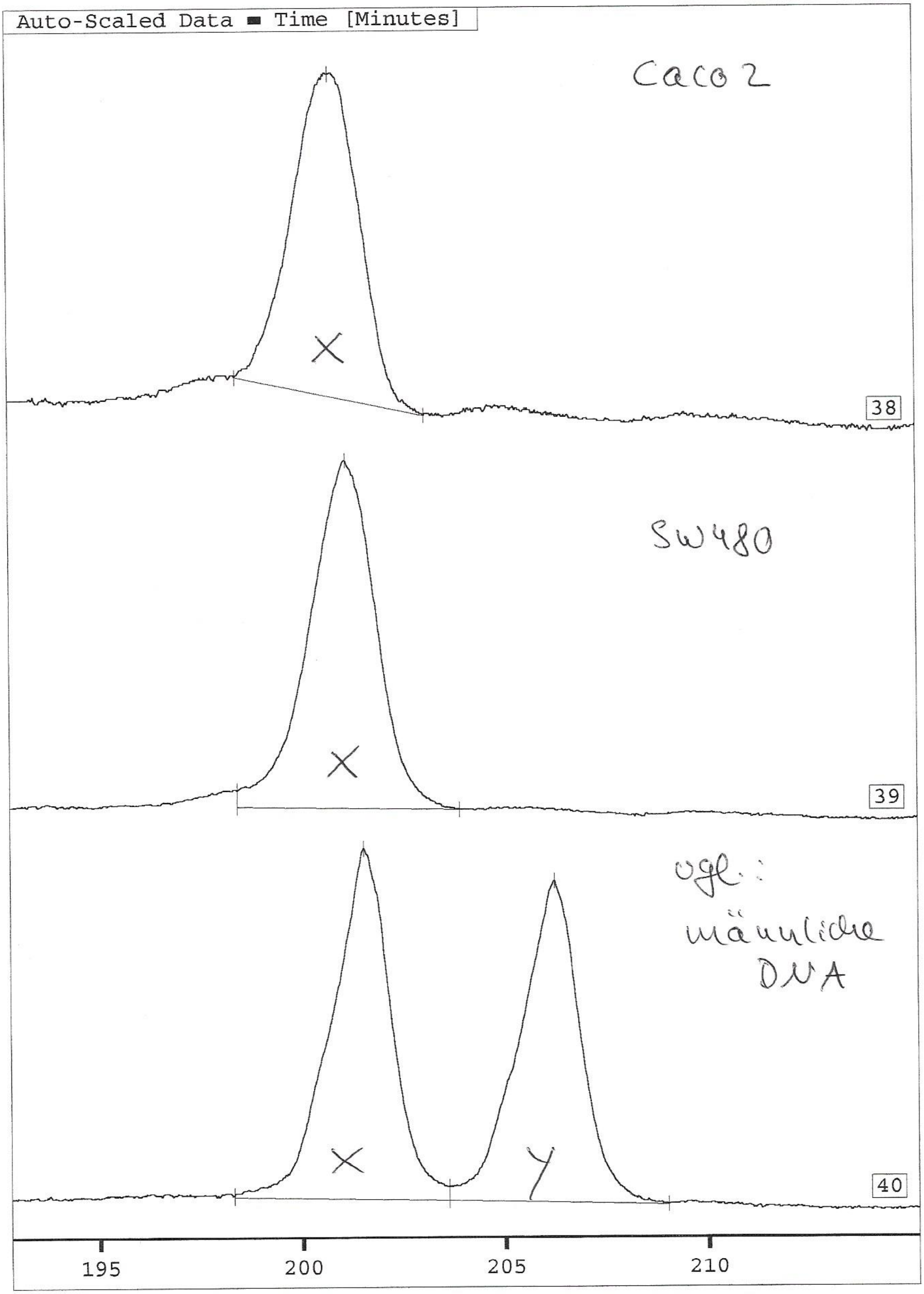

Supplement: Supplementary file 17 — Source Data [file 41467_2020_17334_MOESM17_ESM.zip › SourceData/CellLines/Hoermann_CellLineVerification.pdf]
