# Supplementary material for: Dissecting the sequence determinants for dephosphorylation by the catalytic subunits of phosphatases PP1 and PP2A
Source: Nat Commun. 2020 Jul 17;11:3583. doi: 10.1038/s41467-020-17334-x (PMC7367873; doi:10.1038/s41467-020-17334-x)
Supplement: Supplementary file 17 — Source Data [file 41467_2020_17334_MOESM17_ESM.zip › SourceData/CellLines/Hoermann_MycoplasmaTest.pdf]

MycoplasmaCheck Data Report

Report date: Apr 19, 2020 8:59 PM

Customer: Simone Fehrenbach

Dear FrauSimone Fehrenbach,

Many thanks for your order. The mycoplasma test was conducted for the following mycoplasma species: *M. arginini, M. fermentans, M. orale, M. hyorhinis, M. hominis, M. genitalium, M. salivarium, M. synoviae, M. pirum, M. gallisepticum, M. pneumoniae, M. yeatsii, Spiroplasma citri* and *Acholeplasma laidlawii*. Please note the test is not restricted to the mentioned species. In *silico* analysis has shown that more than 100 additional Mollicutes strains can be detected.

Possible inhibition of the PCR reaction was verified by an internal control. Additional mycoplasma positive and negative controls were included to monitor the results:

- Water controls indicated the absence of PCR contamintations.
- Using plasmid dilutions a detection limit of 10 mycoplasma copies per test was demonstrated.

The result files are available in your account at www.eurofinsgenomics.eu

Eurofins Genomics Europe Applied Genomics GmbH
Anzinger Strasse 7 a 85560 Ebersberg Germany

Tel.: +49 8092 8289-200
Fax: +49 8092 8289-201
Email: info-eu@eurofins.com
Web: eurofinsgenomics.com

Managing Directors: Dr. Michael Hadem,
Dr. Peter Persigehl
Register Court Munich HRB 207710
VAT ID: DE815473648

HypoVereinsbank
IBAN: DE23 2073 0017 7000 0006 50
SWIFT: HYVEDEMEE17

© Eurofins Genomics Europe Applied Genomics GmbH

eurofins Genomics

Table 1: Sample and production details

Identification			Results			
Job no.	Barcode	Cell line name	Testing date	PCR inhib. *	Mycoplasma	Summary
	61282445	HeLa Kyoto	21/02/2020	absent	absent	clean
	61282452	Caco2-Bbe	18/03/2020	absent	absent	clean
	61282469	SW480	18/03/2020	absent	absent	clean

* PCR inhibition: present, will result in an invalid mycoplasma test

Eurofins Genomics Europe Applied Genomics GmbH
Anzinger Strasse 7 a 85560 Ebersberg Germany

Tel.: +49 8092 8289-200
Fax: +49 8092 8289-201
Email: info-eu@eurofins.com
Web: eurofinsgenomics.com

Managing Directors: Dr. Michael Hadem,
Dr. Peter Persigehl
Register Court Munich HRB 207710
VAT ID: DE815473648

HypoVereinsbank
IBAN: DE23 2073 0017 7000 0006 50
SWIFT: HYVEDEMEE17

© Eurofins Genomics Europe Applied Genomics GmbH
