# Supplementary material for: Dissecting the sequence determinants for dephosphorylation by the catalytic subunits of phosphatases PP1 and PP2A
Source: Nat Commun. 2020 Jul 17;11:3583. doi: 10.1038/s41467-020-17334-x (PMC7367873; doi:10.1038/s41467-020-17334-x)

Single Injection Report

Agilent Technologies

Sample Name PDP Nal

Injection Acquired Date 2/11/2020 1:10:47 PM

Sample Description

Injection Acq Method Name 10 to 90 ACN over 15 min - 18 min Total.M

Injection Data File Directory D:\Data\Gosia\GosiaTemplate 2020-02-11 13-08-02

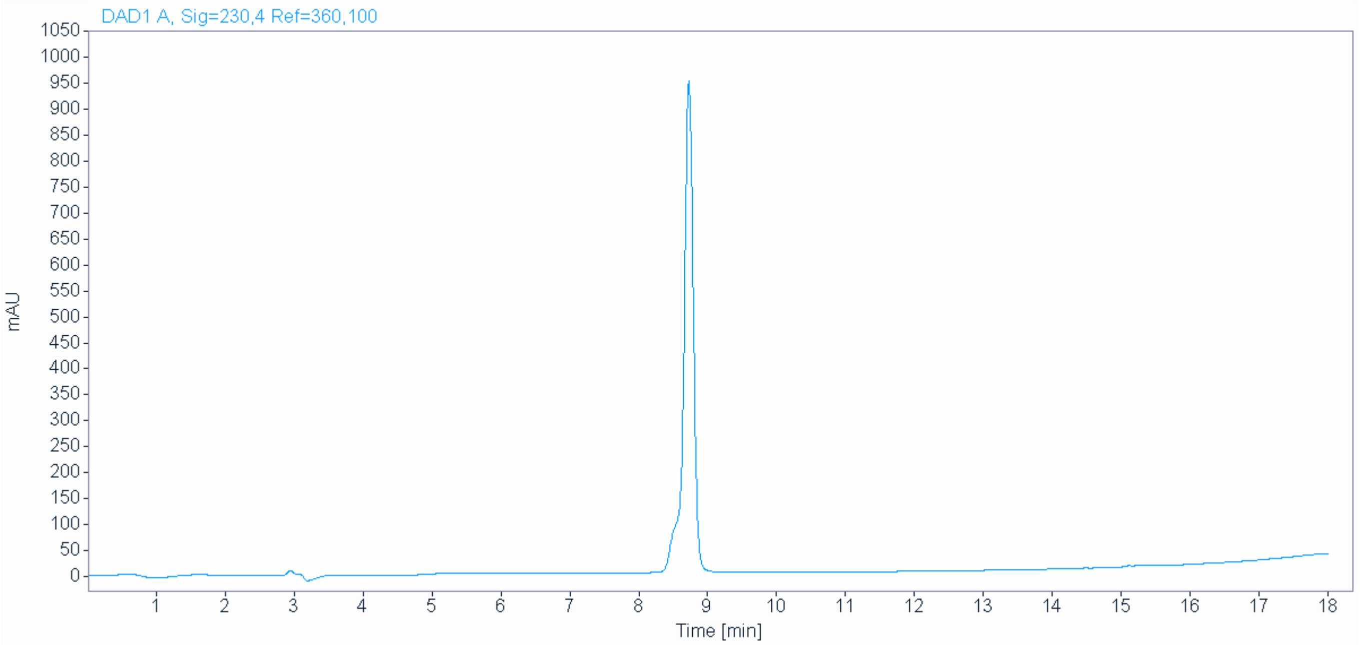

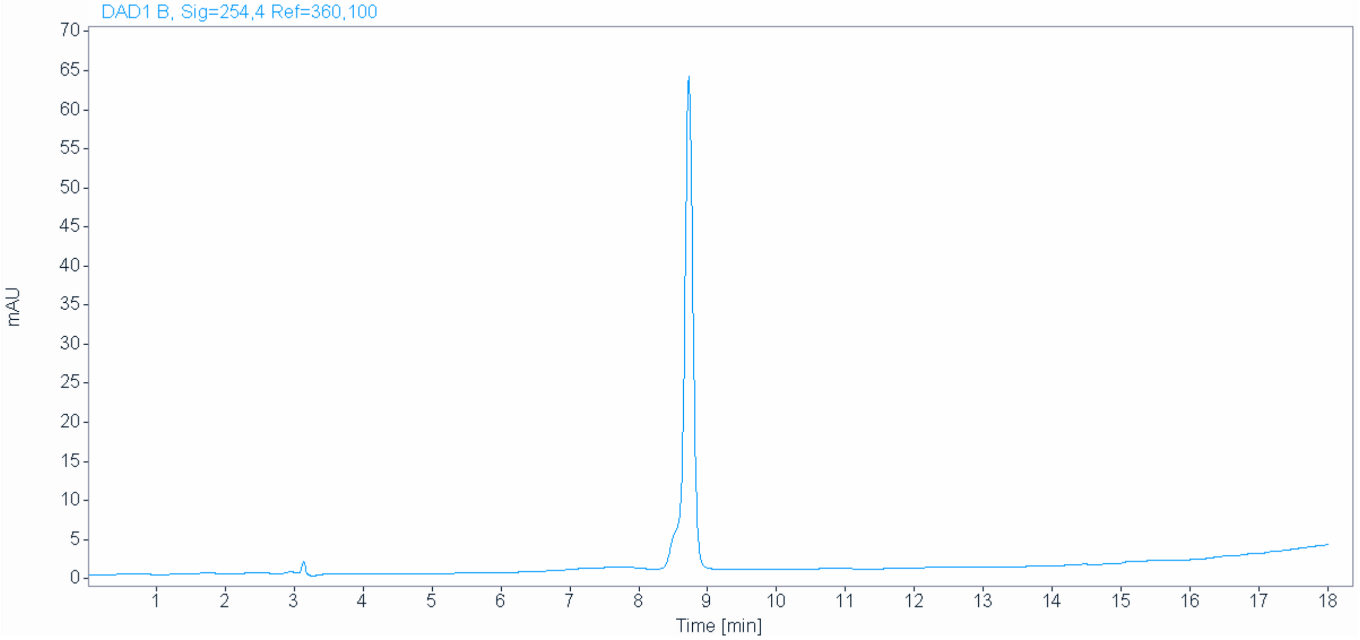

Agilent Technologies

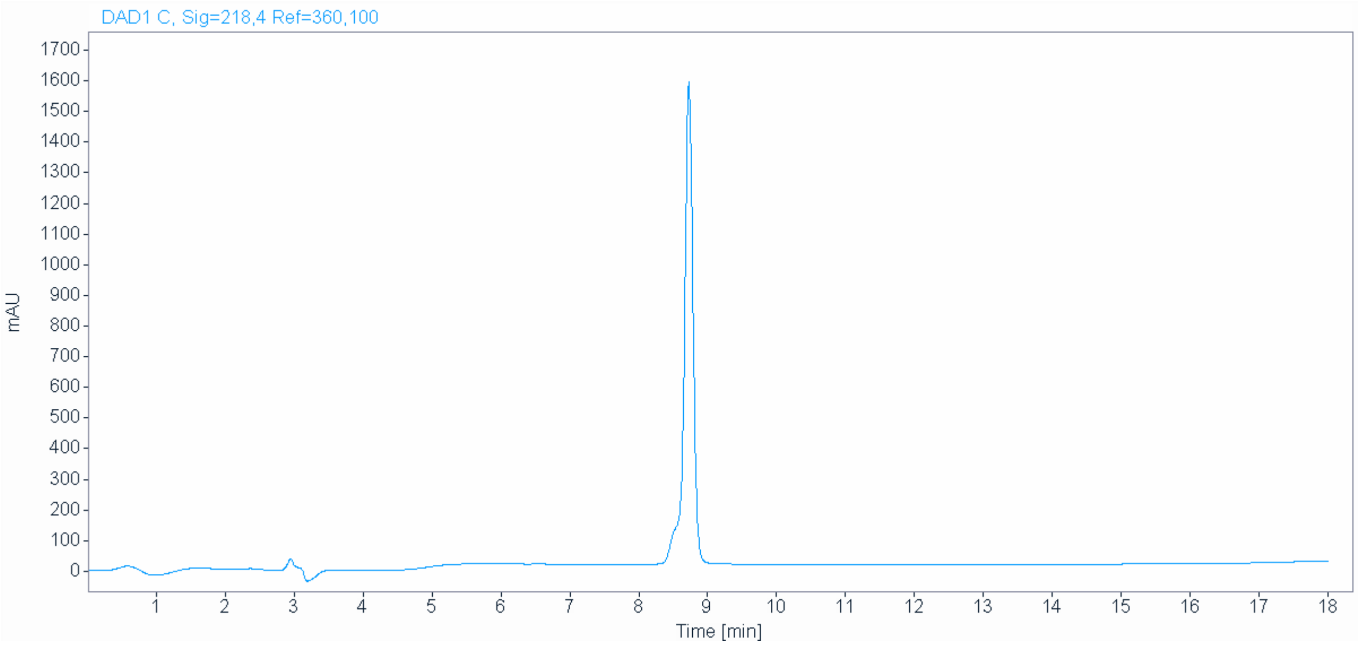

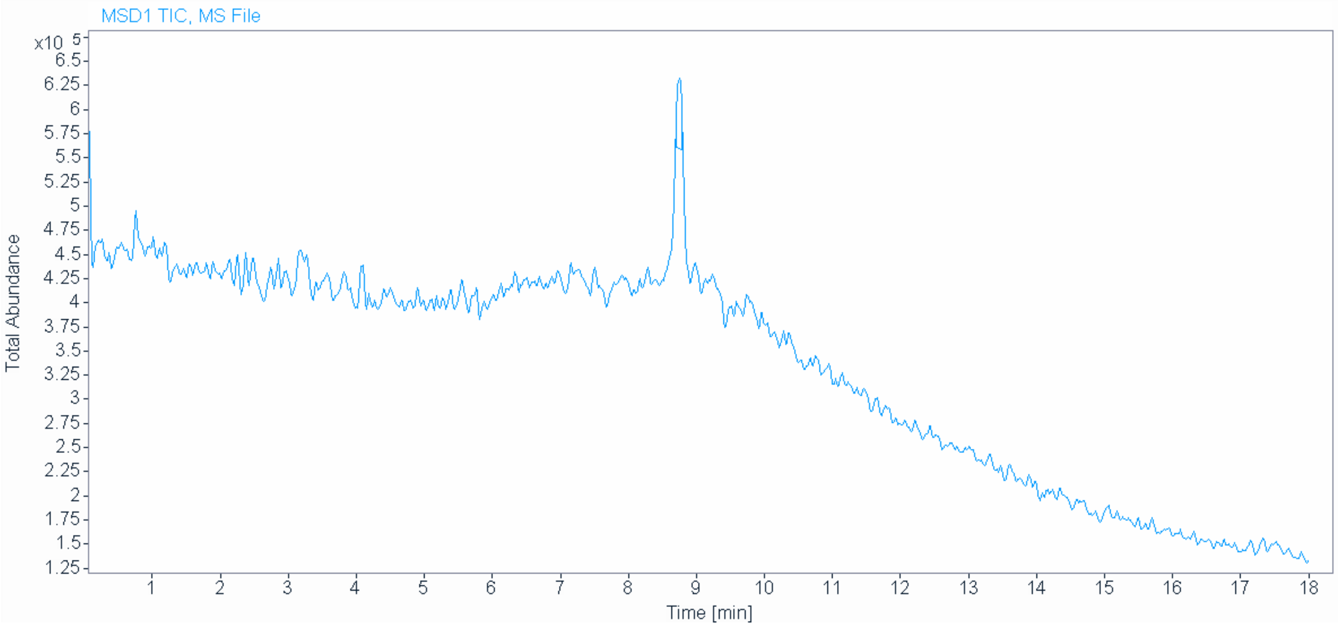

Agilent Technologies

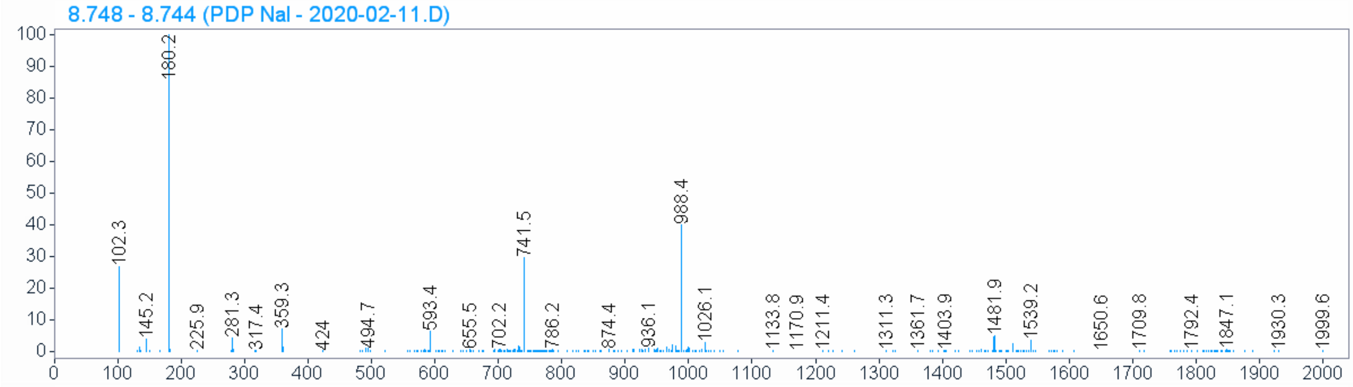

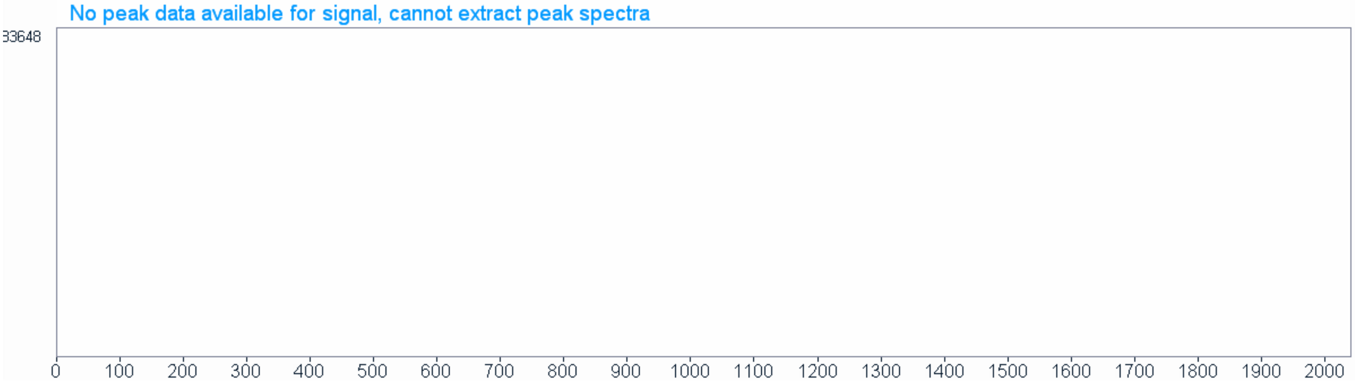

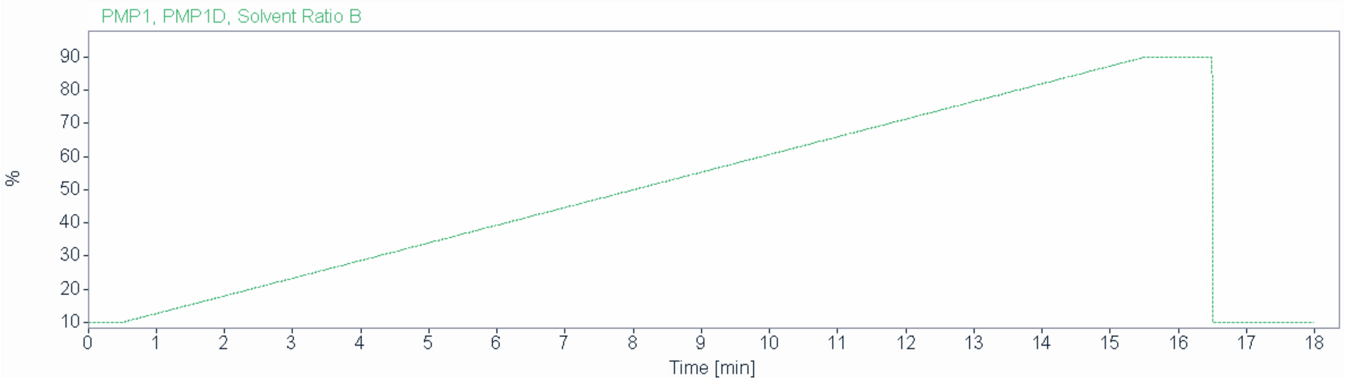

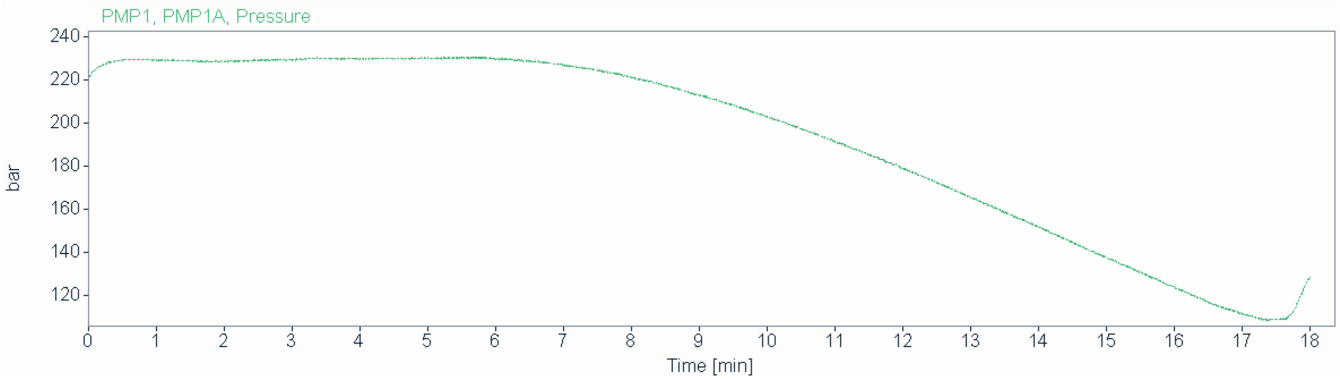

Supplement: Supplementary file 17 — Source Data [file 41467_2020_17334_MOESM17_ESM.zip › SourceData/PeptideSynthesis/PDPs/PDP-Nal.pdf]
