# Supplementary material for: Dissecting the sequence determinants for dephosphorylation by the catalytic subunits of phosphatases PP1 and PP2A
Source: Nat Commun. 2020 Jul 17;11:3583. doi: 10.1038/s41467-020-17334-x (PMC7367873; doi:10.1038/s41467-020-17334-x)
Supplement: Supplementary file 17 — Source Data [file 41467_2020_17334_MOESM17_ESM.zip › SourceData/PeptideSynthesis/PDPs/PDPm-Nal.pdf]

# Single Injection Report

Agilent Technologies

| | |
|---|---|
| Sample Name | PDPm Nal |
| Injection Acquired Date | 2/11/2020 1:30:01 PM |
| Sample Description | |
| Injection Acq Method Name | 10 to 90 ACN over 15 min - 18 min Total.M |
| Injection Data File Directory | D:\Data\Gosia\GosiaTemplate 2020-02-11 13-08-02 |

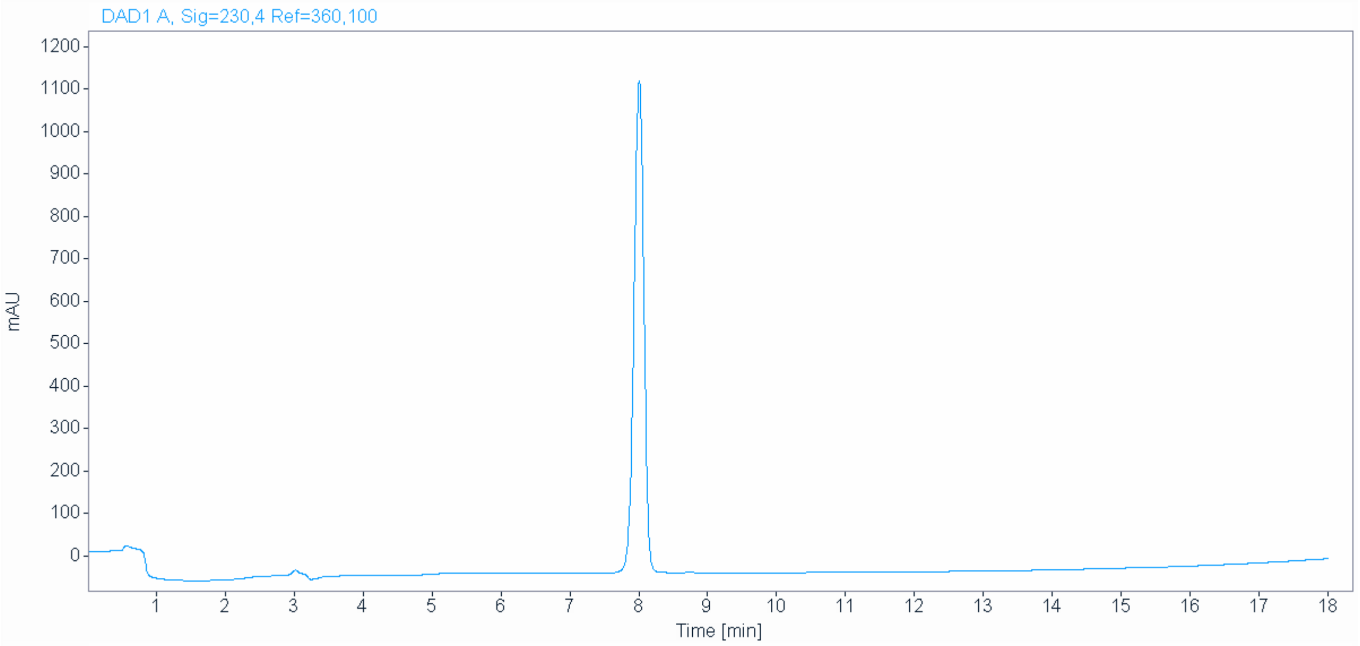


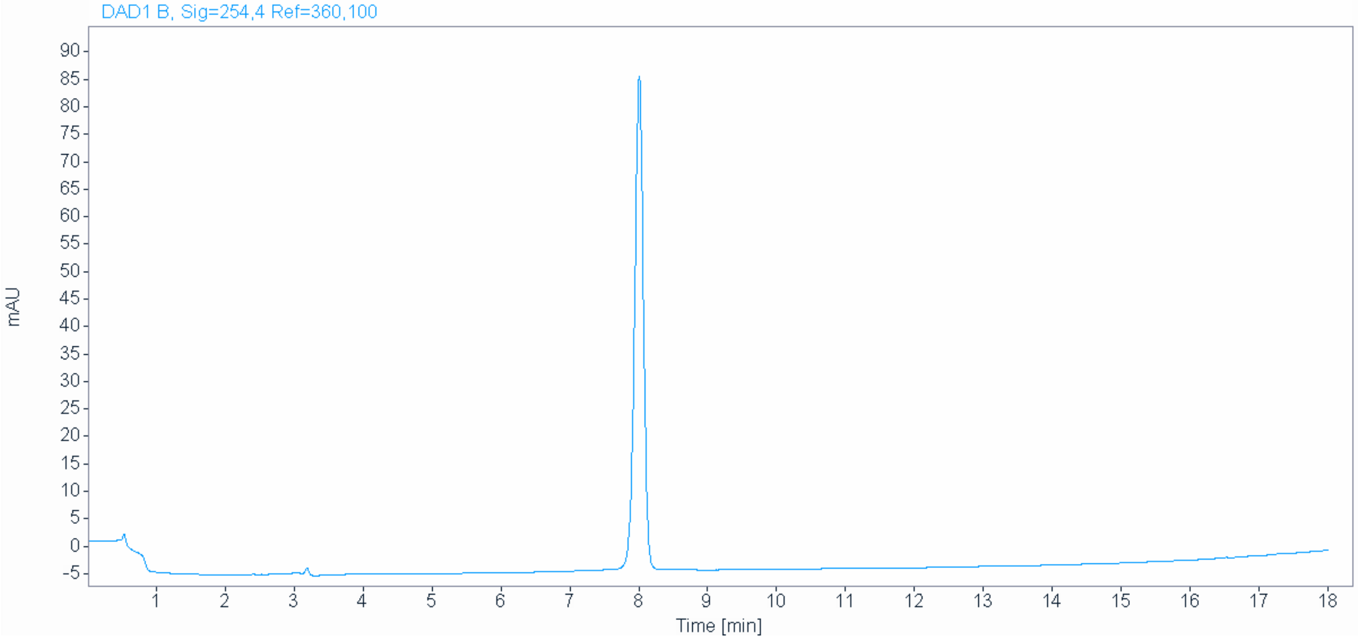

## Single Injection Report

Agilent Technologies

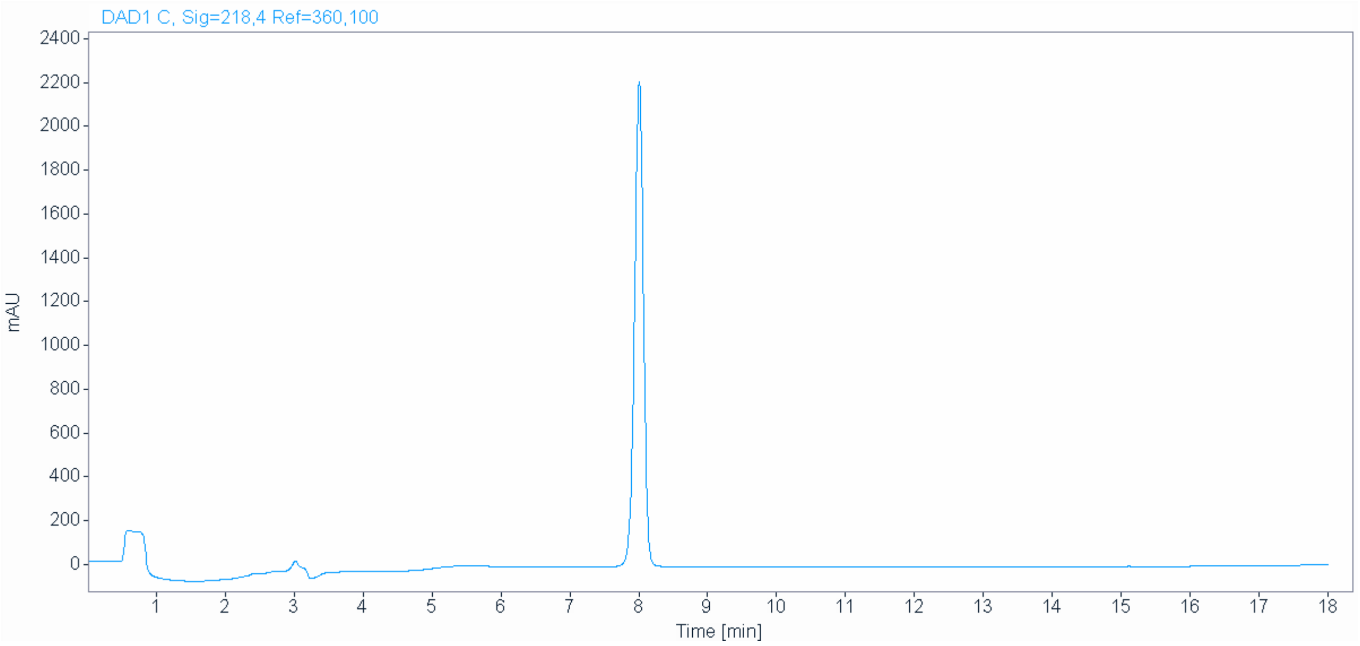


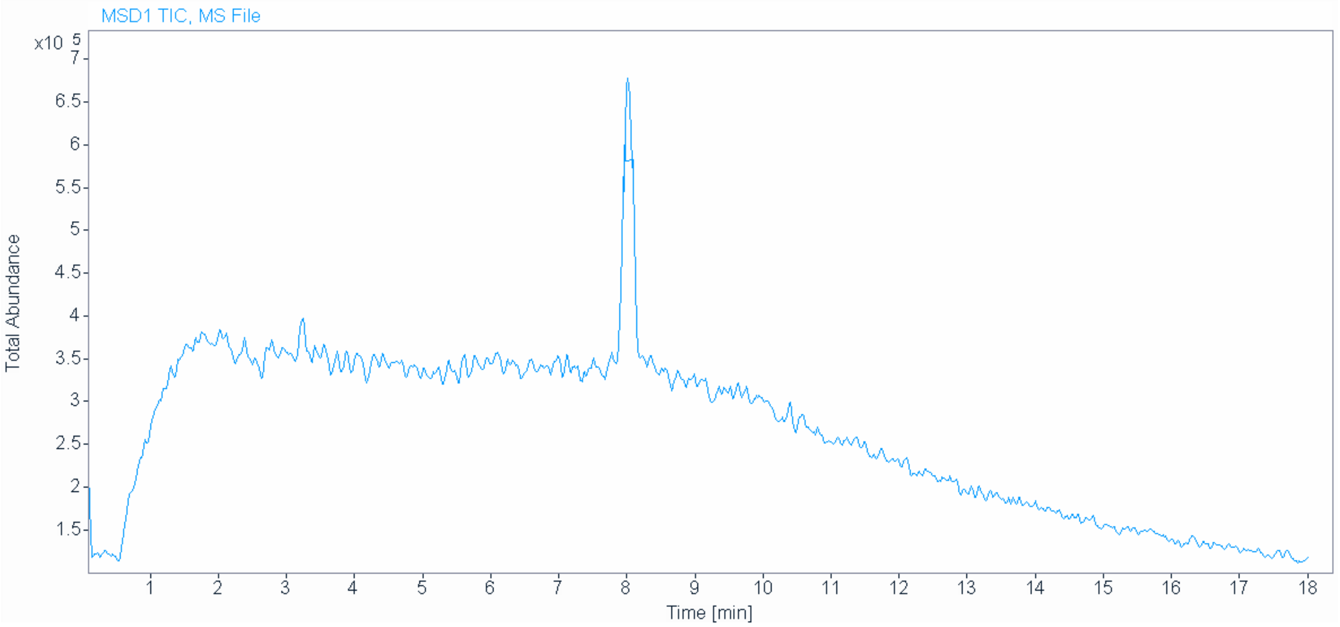

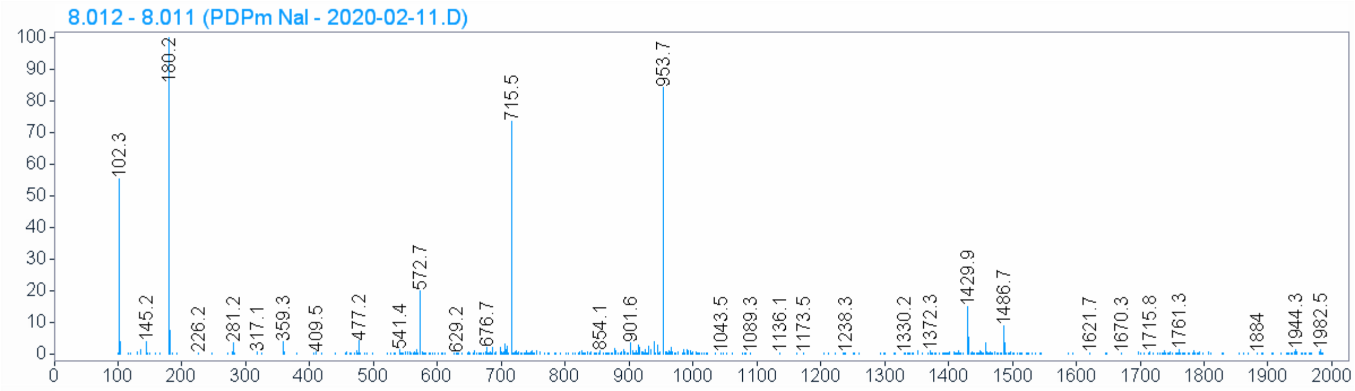

8.012 - 8.011 (PDPm NaI - 2020-02-11.D)
102.3
180.2
145.2
226.2
281.2
317.1
359.3
409.5
477.2
541.4
572.7
629.2
676.7
715.5
854.1
901.6
953.7
1043.5
1089.3
1136.1
1173.5
1238.3
1330.2
1372.3
1429.9
1486.7
1621.7
1670.3
1715.8
1761.3
1884
1944.3
1982.5


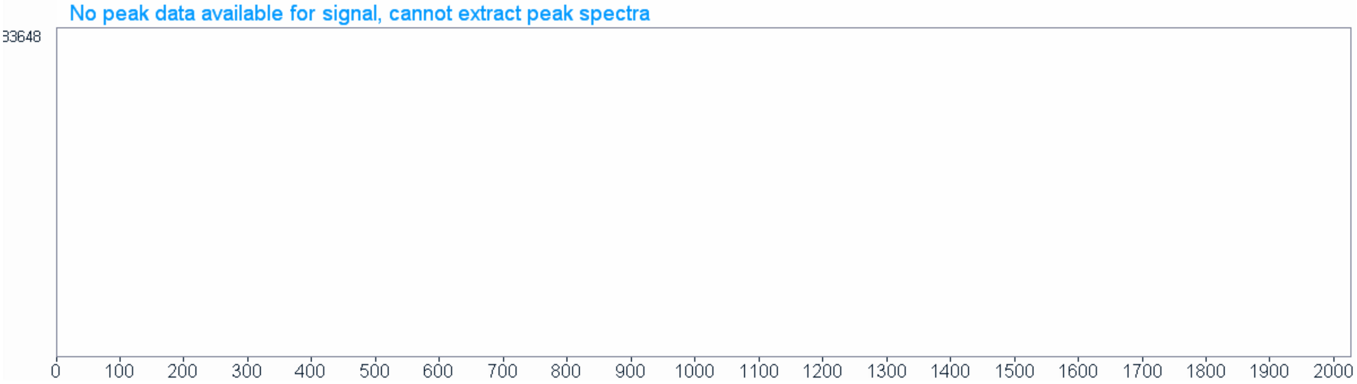

No peak data available for signal, cannot extract peak spectra
33648


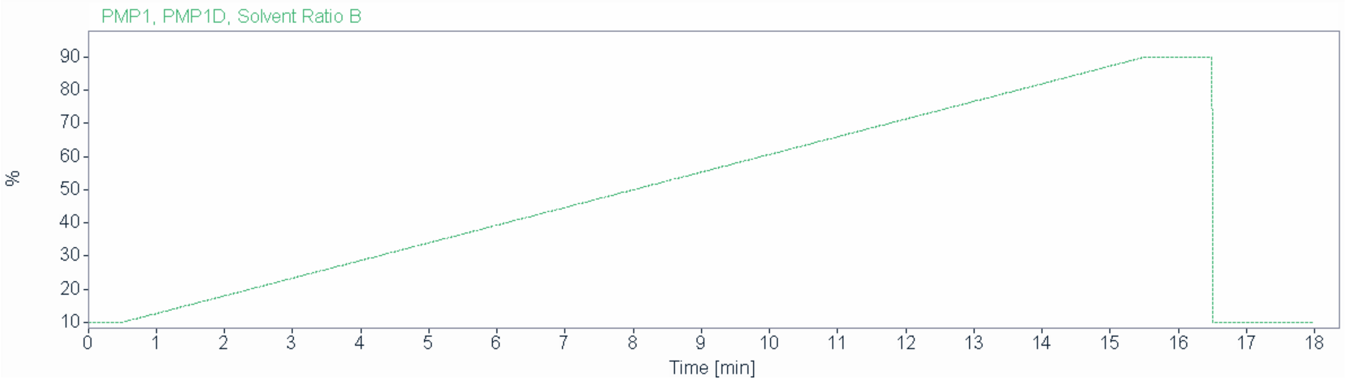

PMP1, PMP1D, Solvent Ratio B
%
Time [min]


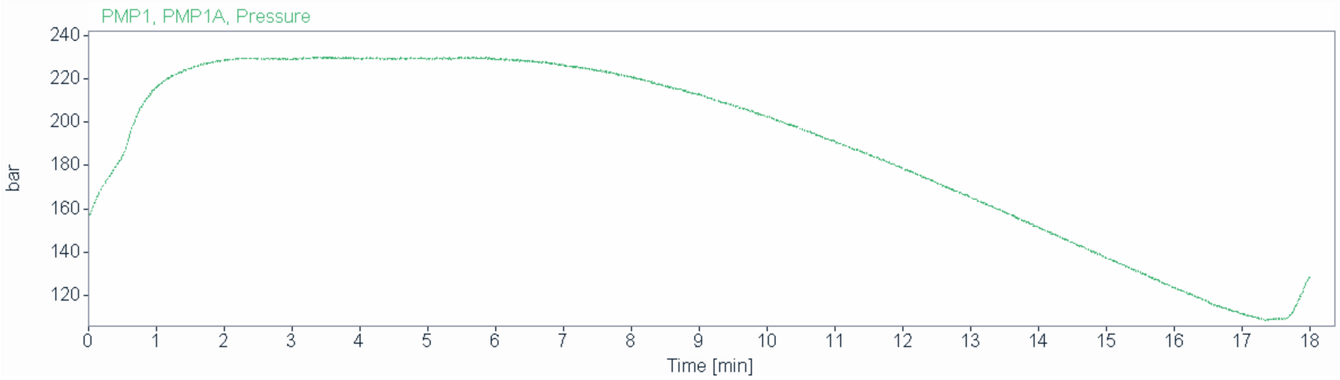

PMP1, PMP1A, Pressure
bar
Time [min]
