# Supplementary material for: Dissecting the sequence determinants for dephosphorylation by the catalytic subunits of phosphatases PP1 and PP2A
Source: Nat Commun. 2020 Jul 17;11:3583. doi: 10.1038/s41467-020-17334-x (PMC7367873; doi:10.1038/s41467-020-17334-x)
Supplement: Supplementary file 17 — Source Data [file 41467_2020_17334_MOESM17_ESM.zip › SourceData/PeptideSynthesis/PLDMS_verification/AAAApTLFGAK_report.pdf]

# Single Injection Report

Agilent Technologies

| | |
|---|---|
| Sample Name | PTK31_purified |
| Injection Acquired Date | 4/3/2020 10:59:45 AM |
| Sample Description | |
| Injection Acq Method Name | Nico- 10 to 90 ACN 15 min - 20 min Total.M |
| Injection Data File Directory | D:\Data\Thomas\Thomas 2020-04-03 10-56-08 |

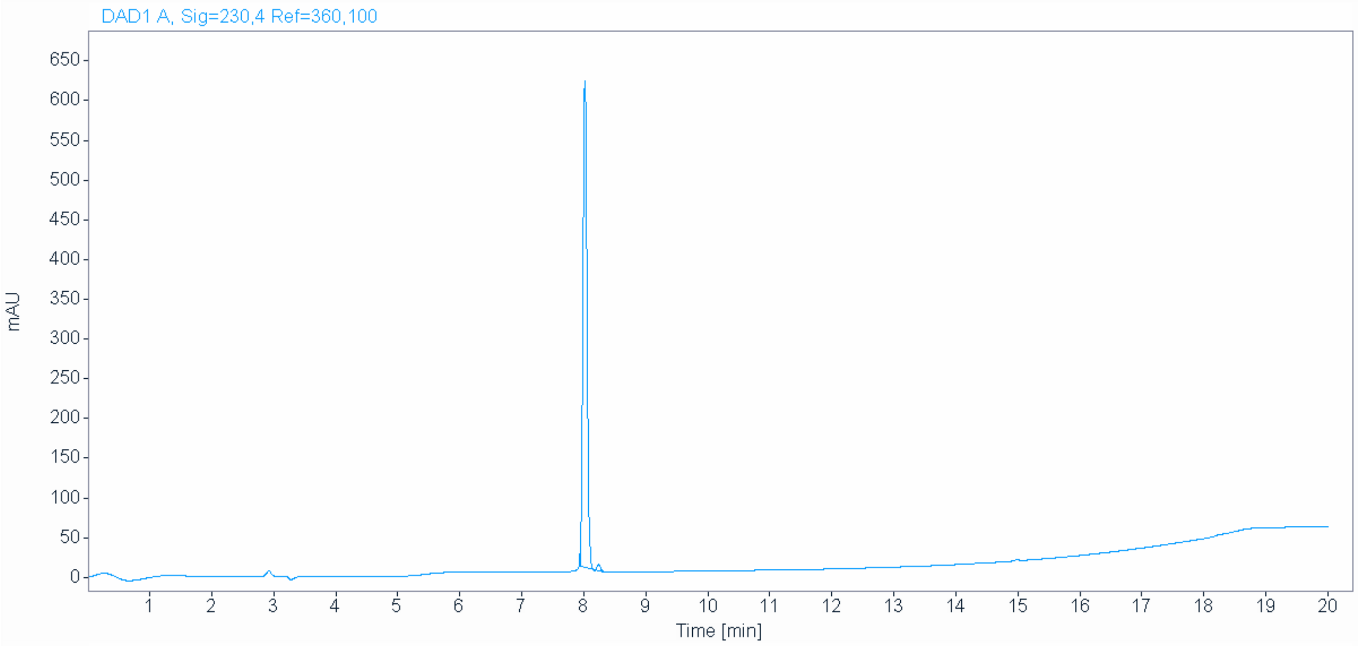


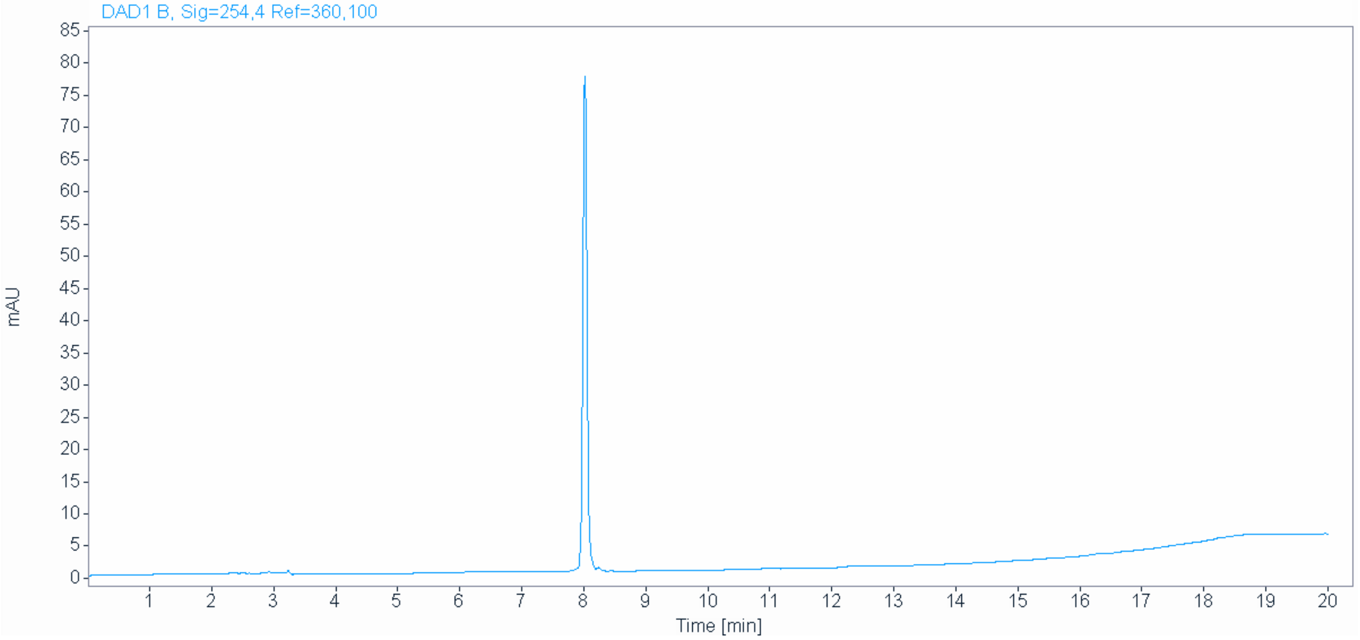

Agilent Technologies

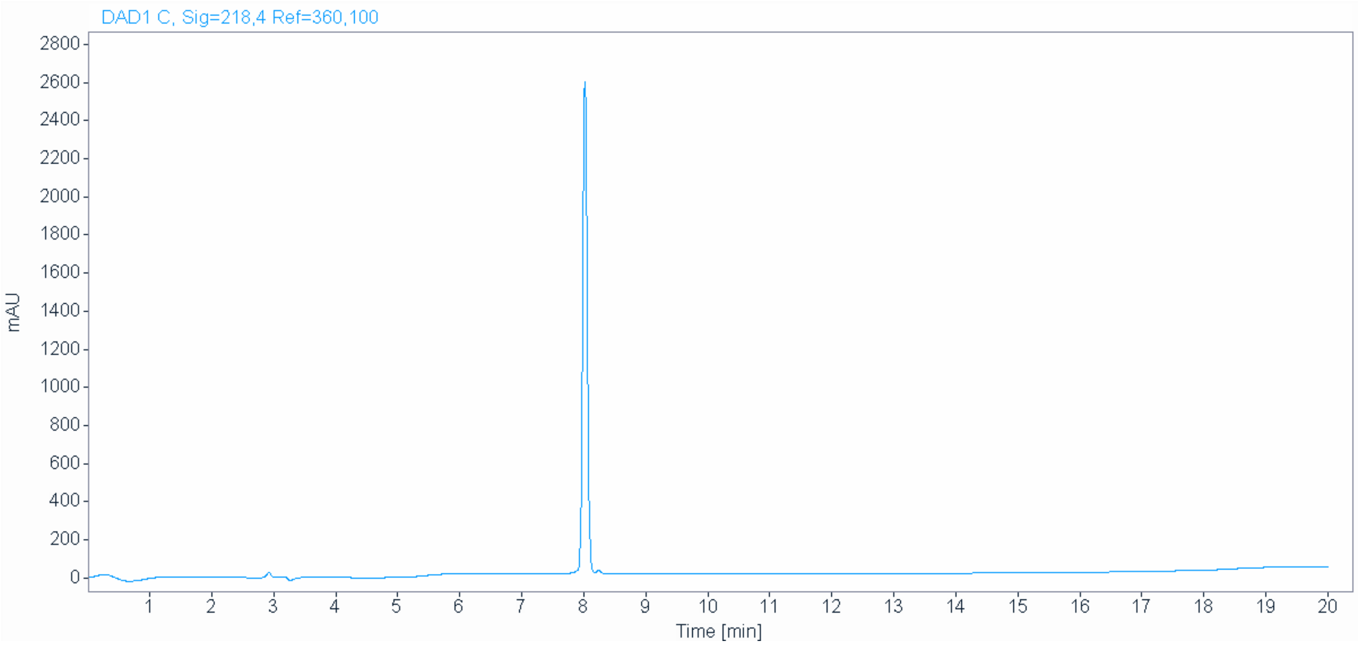


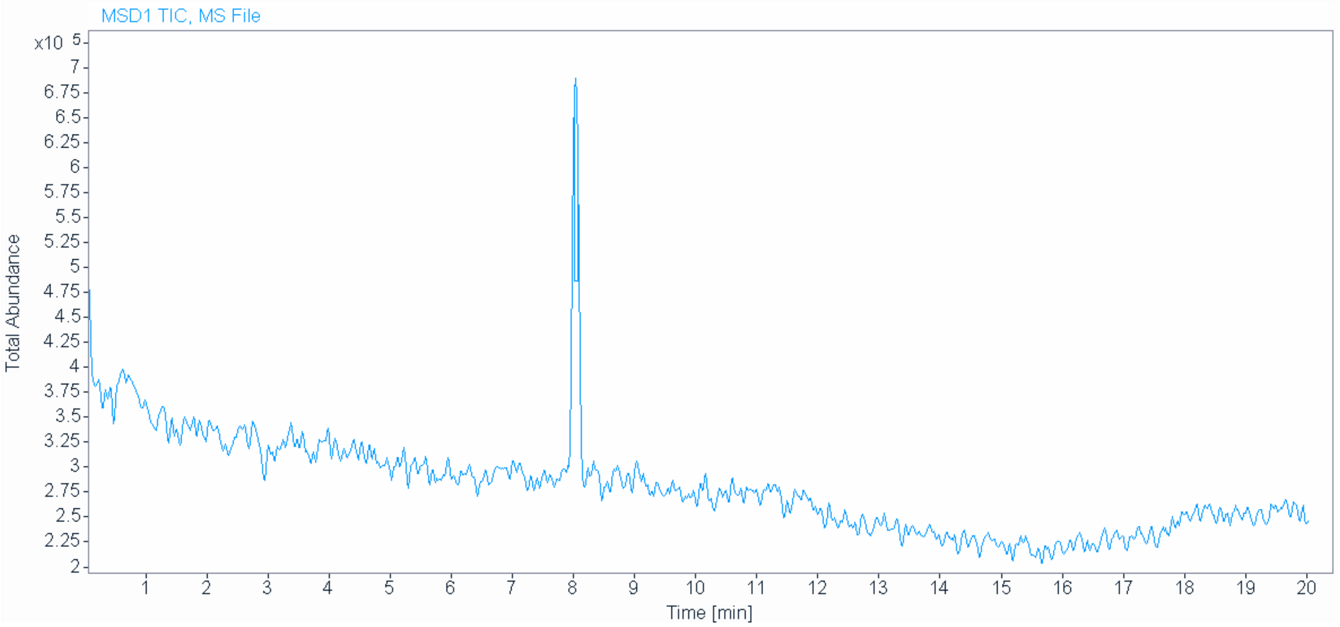

Agilent Technologies

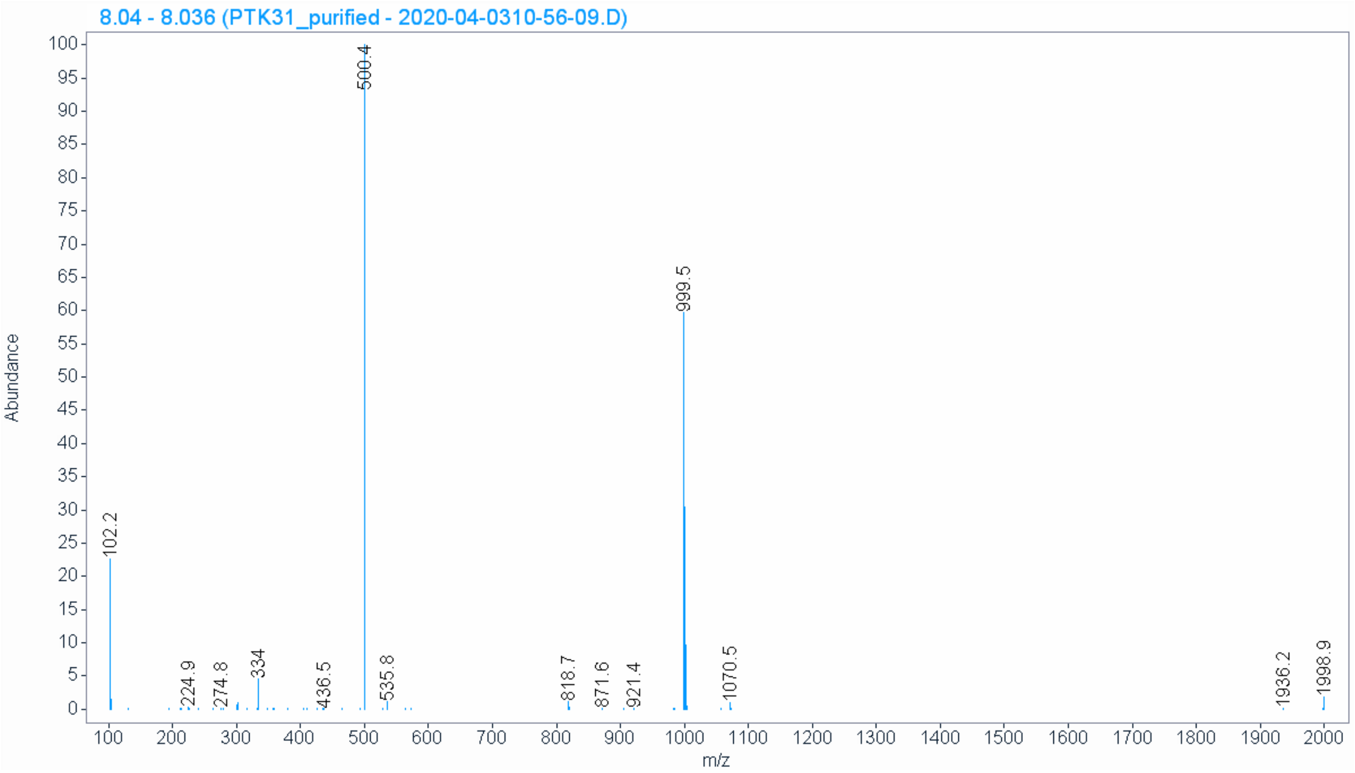


**Signal:** DAD1 A, Sig=230,4 Ref=360,100

| RT [min] | Type | Width [min] | Area | Height | Area% | Name |
|---|---|---|---|---|---|---|
| 8.017 | MF | 0.0769 | 2835.9534 | 614.8544 | 98.9040 | |
| 8.242 | FM | 0.0647 | 31.4252 | 8.1011 | 1.0960 | |
| | | Sum | 2867.3786 | | | |

**Signal:** MSD1 TIC, MS File

| RT [min] | Type | Width [min] | Area | Height | Area% | Name |
|---|---|---|---|---|---|---|
| 8.040 | MM | 0.0524 | 645935.8125 | 205411.0469 | 100.0000 | |
| | | Sum | 645935.8125 | | | |

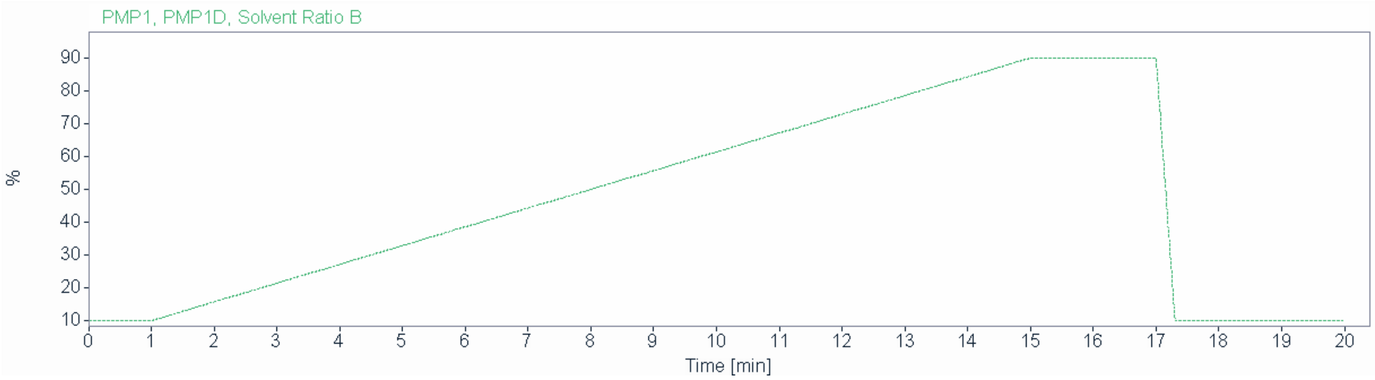
PMP1, PMP1D, Solvent Ratio B
%
90
80
70
60
50
40
30
20
10
0 1 2 3 4 5 6 7 8 9 10 11 12 13 14 15 16 17 18 19 20
Time [min]

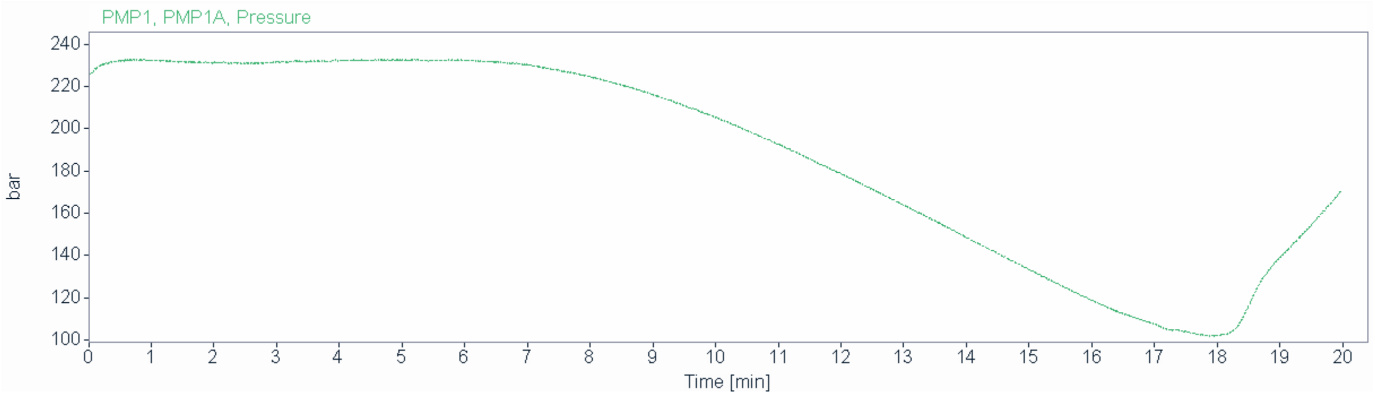
PMP1, PMP1A, Pressure
bar
240
220
200
180
160
140
120
100
0 1 2 3 4 5 6 7 8 9 10 11 12 13 14 15 16 17 18 19 20
Time [min]
