# Supplementary material for: Dissecting the sequence determinants for dephosphorylation by the catalytic subunits of phosphatases PP1 and PP2A
Source: Nat Commun. 2020 Jul 17;11:3583. doi: 10.1038/s41467-020-17334-x (PMC7367873; doi:10.1038/s41467-020-17334-x)
Supplement: Supplementary file 17 — Source Data [file 41467_2020_17334_MOESM17_ESM.zip › SourceData/PeptideSynthesis/PLDMS_verification/AAAApTPFGAK_report.pdf]

Single Injection Report

Agilent Technologies

Sample Name PTK 29 purification v 33

Injection Acquired Date 3/10/2020 1:59:02 PM

Sample Description

Injection Acq Method Name Nico- 10 to 90 ACN 15 min - 20 min Total.M

Injection Data File Directory D:\Data\Thomas\Thomas 2020-03-10 13-57-14

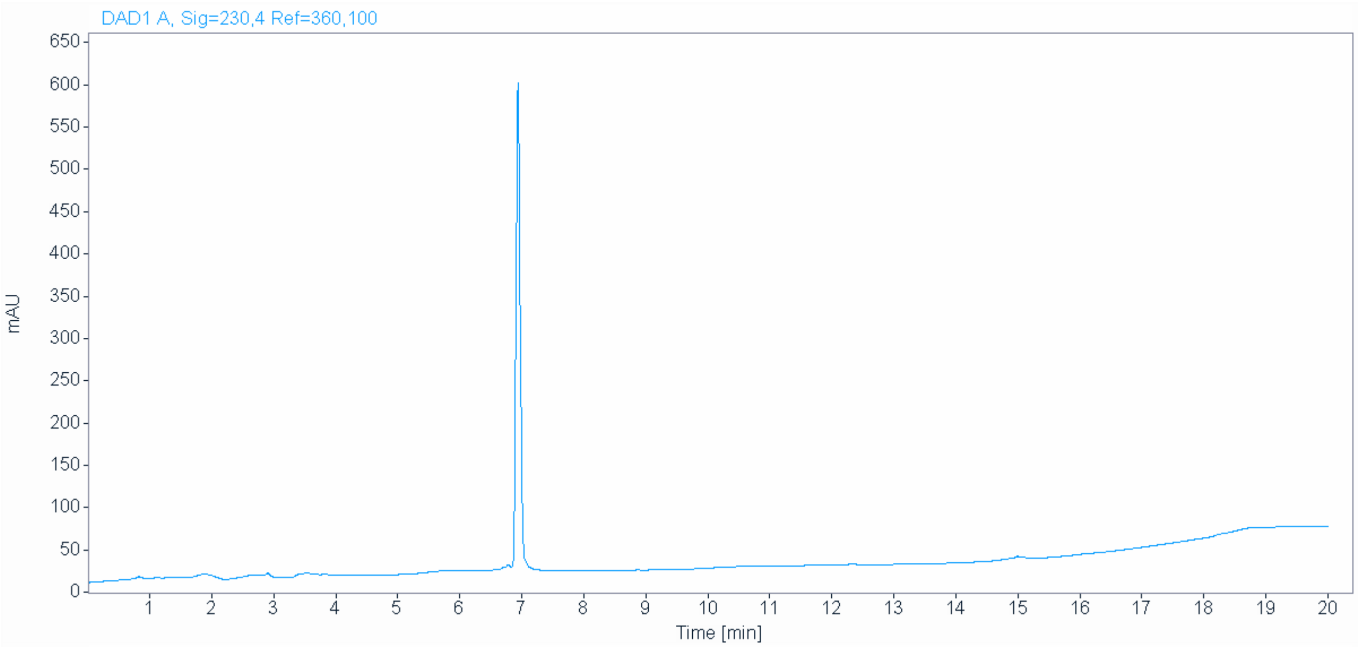

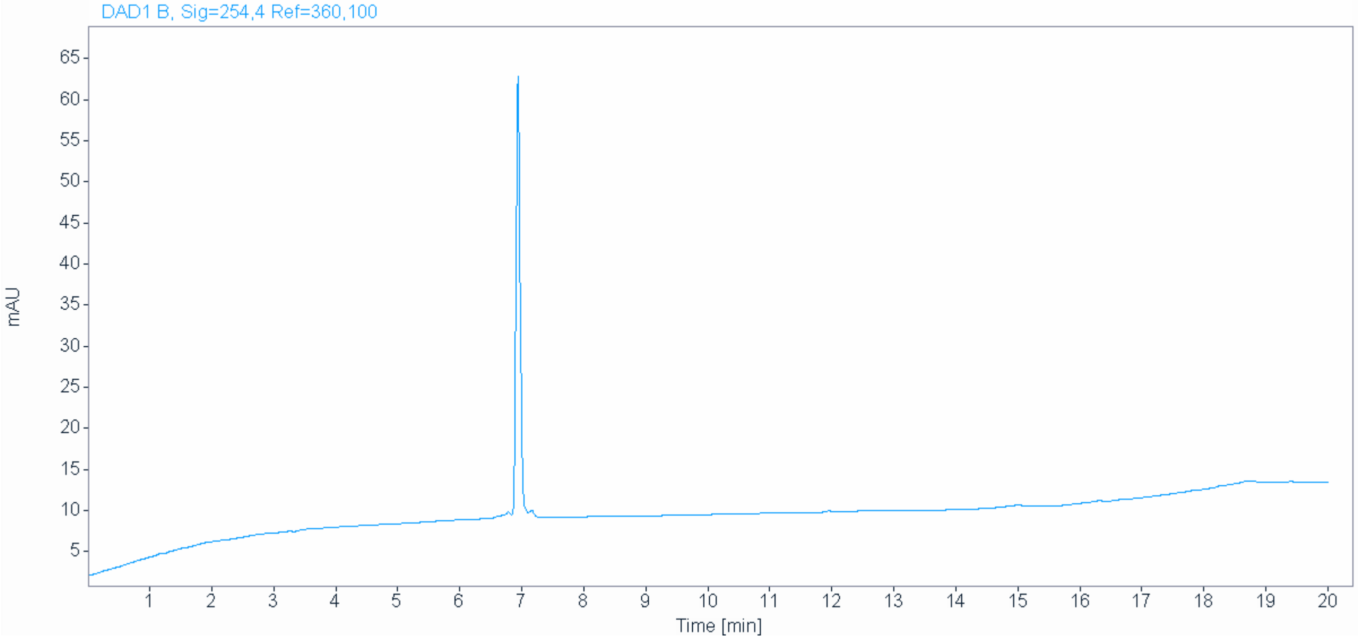

Agilent Technologies

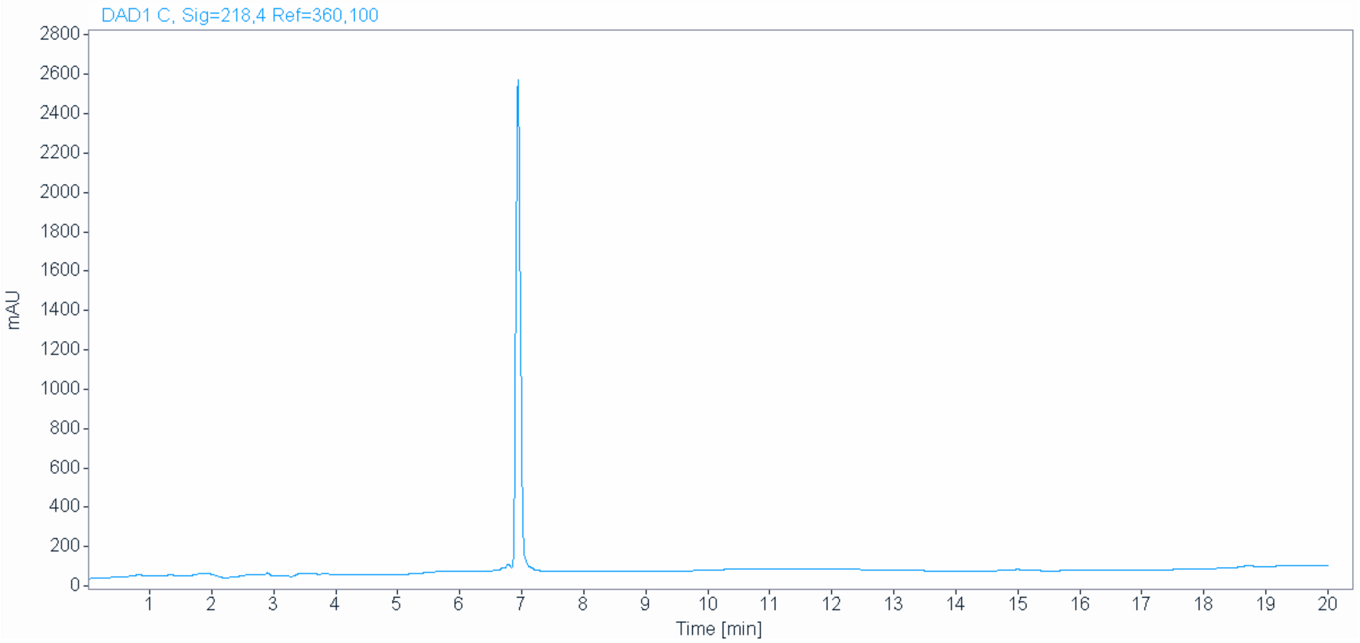

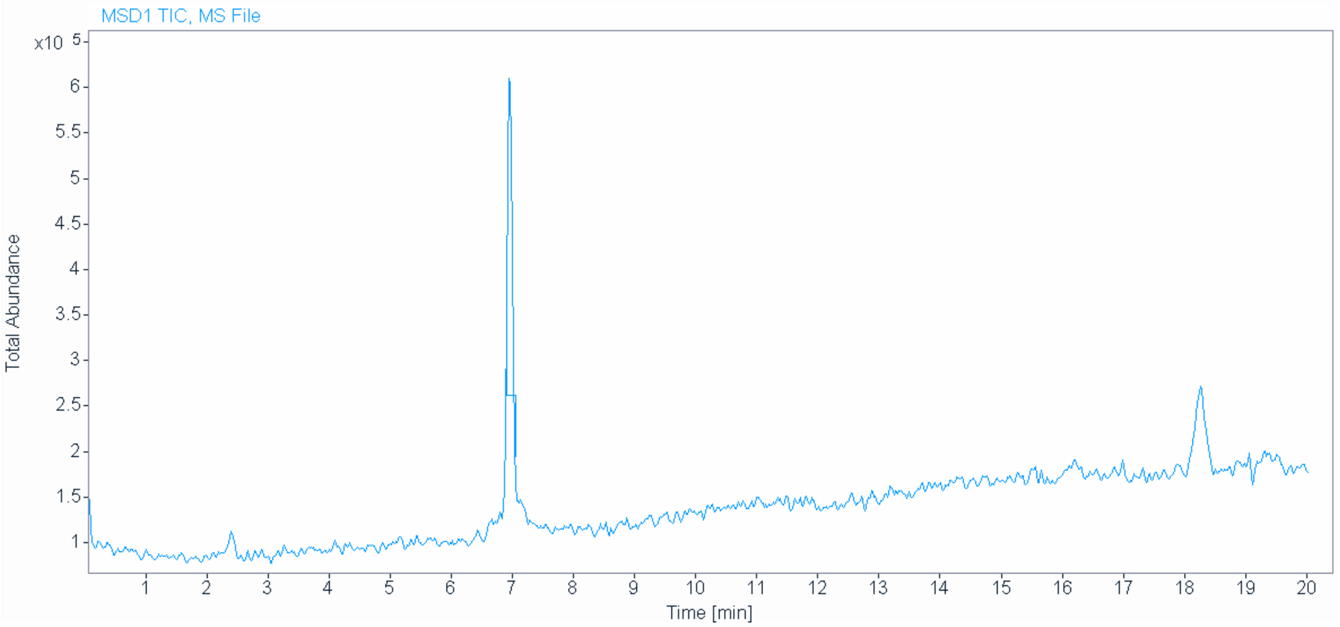

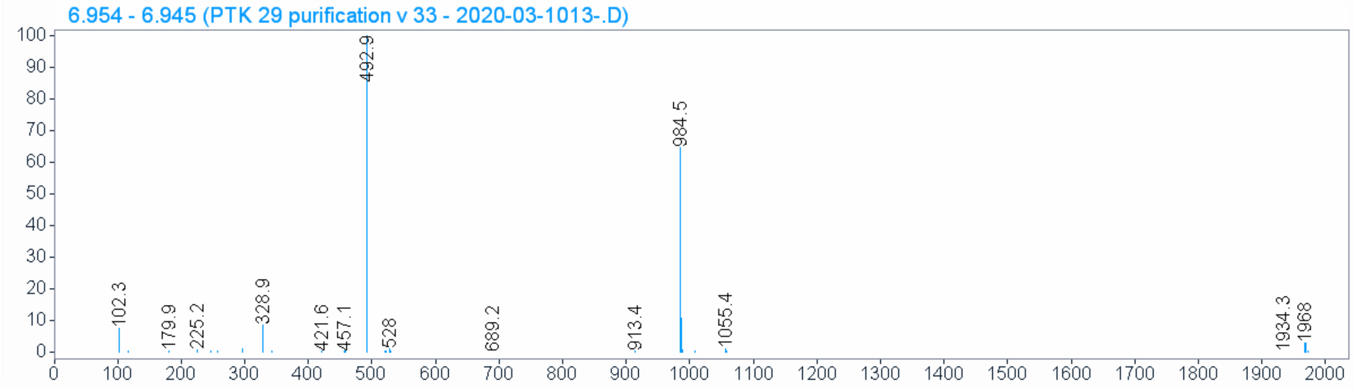
6.954 - 6.945 (PTK 29 purification v 33 - 2020-03-1013-.D)
100
90
80
70
60
50
40
30
20
10
0
102.3
179.9
225.2
328.9
421.6
457.1
492.9
528
689.2
913.4
984.5
1055.4
1934.3
1968
0 100 200 300 400 500 600 700 800 900 1000 1100 1200 1300 1400 1500 1600 1700 1800 1900 2000

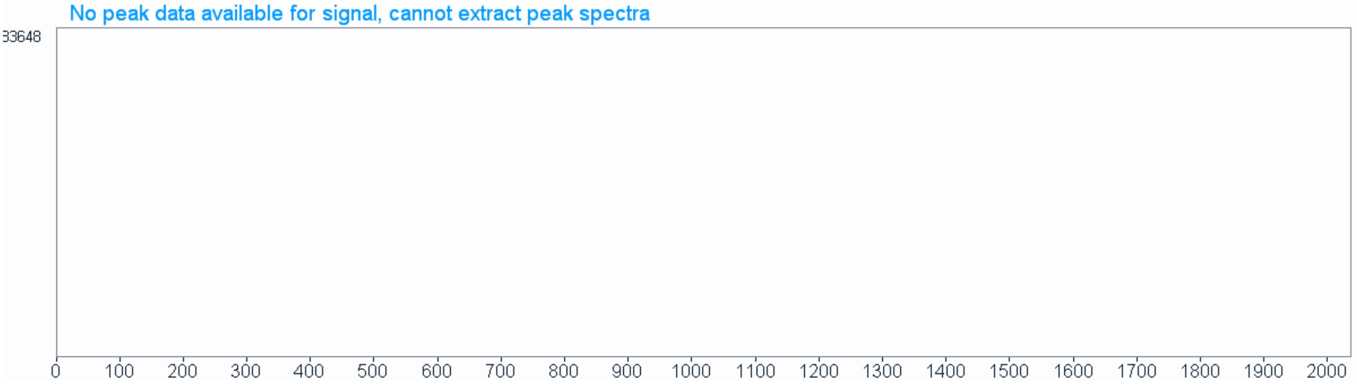
No peak data available for signal, cannot extract peak spectra
33648
0 100 200 300 400 500 600 700 800 900 1000 1100 1200 1300 1400 1500 1600 1700 1800 1900 2000

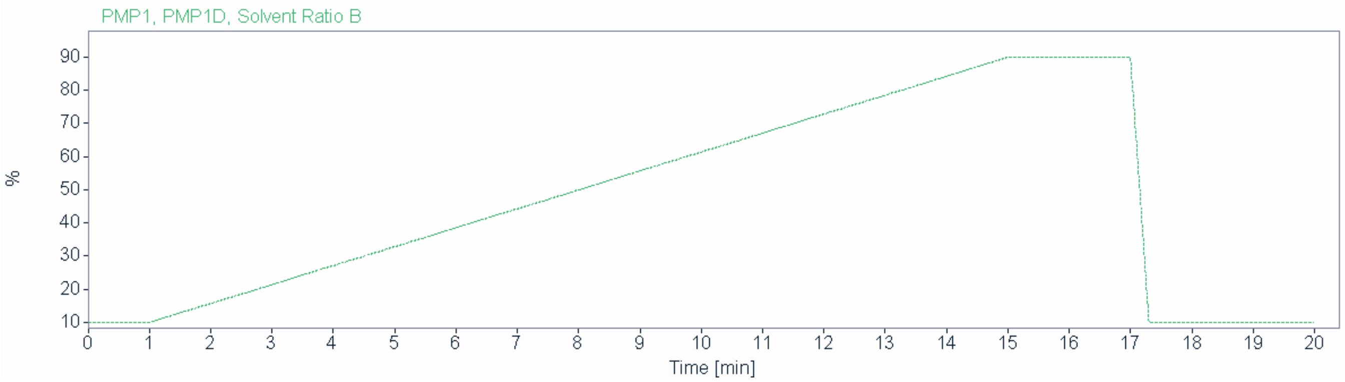
PMP1, PMP1D, Solvent Ratio B
%
90
80
70
60
50
40
30
20
10
0 1 2 3 4 5 6 7 8 9 10 11 12 13 14 15 16 17 18 19 20
Time [min]

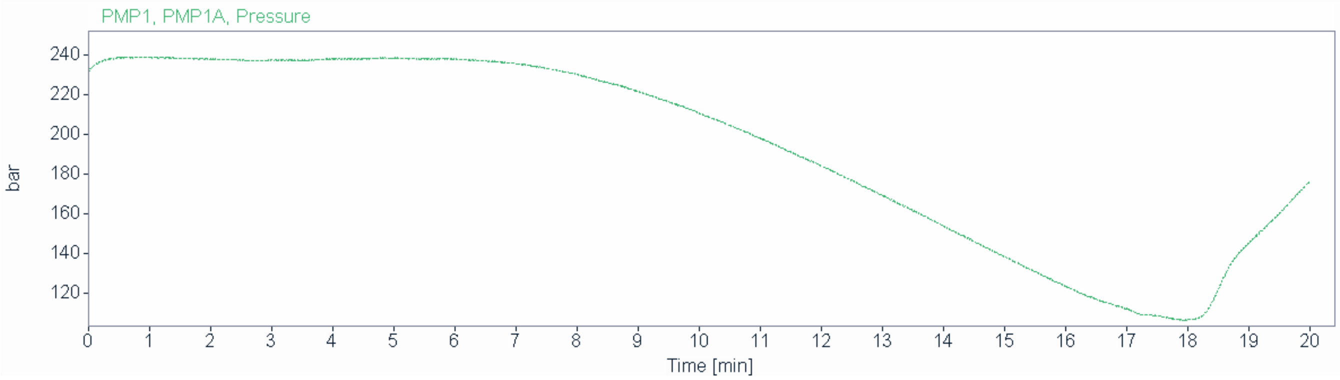
PMP1, PMP1A, Pressure
bar
240
220
200
180
160
140
120
0 1 2 3 4 5 6 7 8 9 10 11 12 13 14 15 16 17 18 19 20
Time [min]
