# Supplementary material for: Dissecting the sequence determinants for dephosphorylation by the catalytic subunits of phosphatases PP1 and PP2A
Source: Nat Commun. 2020 Jul 17;11:3583. doi: 10.1038/s41467-020-17334-x (PMC7367873; doi:10.1038/s41467-020-17334-x)
Supplement: Supplementary file 17 — Source Data [file 41467_2020_17334_MOESM17_ESM.zip › SourceData/PeptideSynthesis/PLDMS_verification/FEAEpTAAAAK_report.pdf]

Single Injection Report

Agilent Technologies

Sample Name	JC-131-5-1+2
Injection Acquired Date	9/20/2018 5:31:01 PM
Sample Description	
Injection Acq Method Name	JC 10 to 90 ACN over 15 min.M
Injection Data File Directory	D:\Data\Old Data\Jeremy\Jeremy Template 2018-09-20 17-29-42

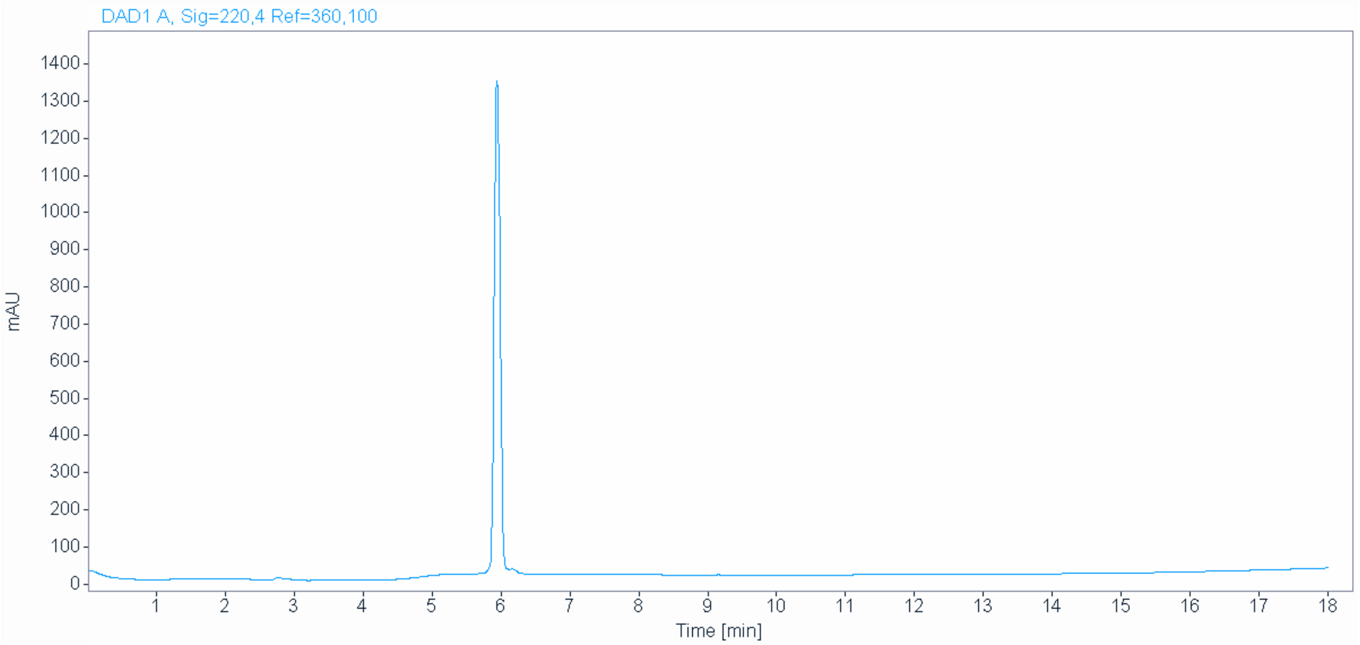

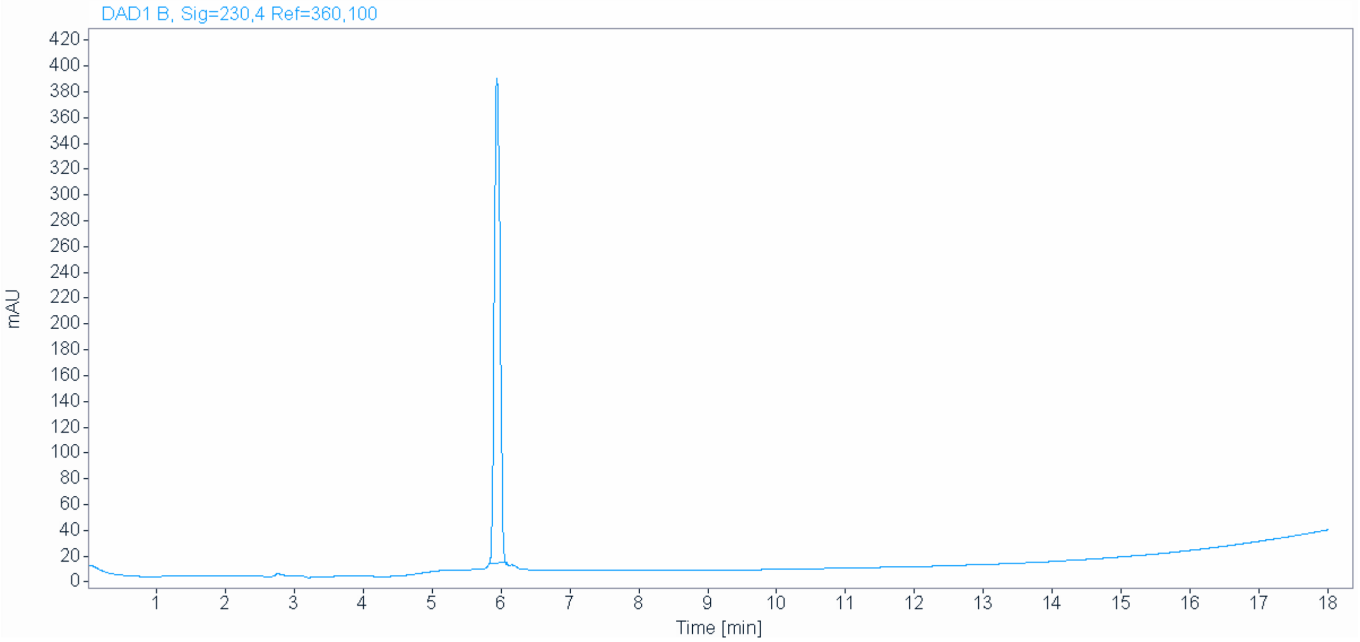

Agilent Technologies

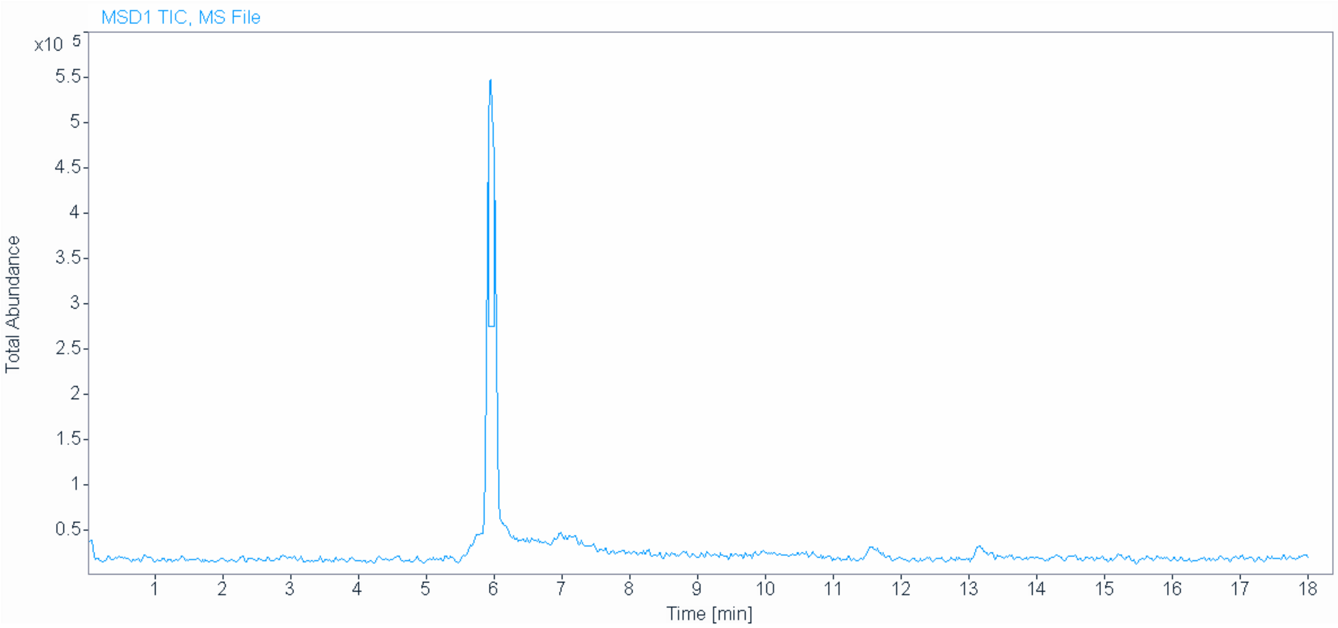

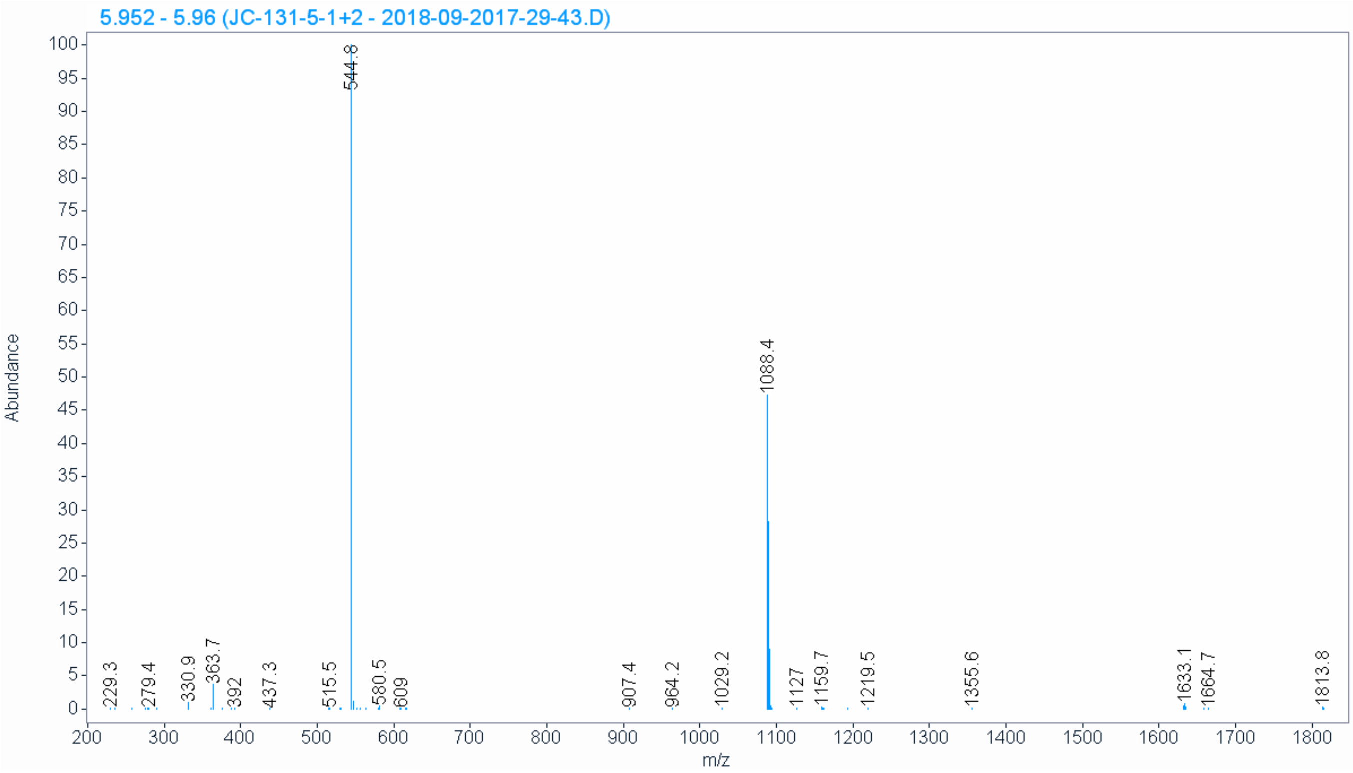

Signal: DAD1 B, Sig=230,4 Ref=360,100

RT [min]	Type	Width [min]	Area	Height	Area%	Name
5.937	MM	0.0973	2203.9214	377.3203	100.0000	
		Sum	2203.9214			

Signal: MSD1 TIC, MS File

RT [min]	Type	Width [min]	Area	Height	Area%	Name
5.952	MM	0.0612	1016328.750<br>0	276920.5313	100.0000	
		Sum	1016328.750			

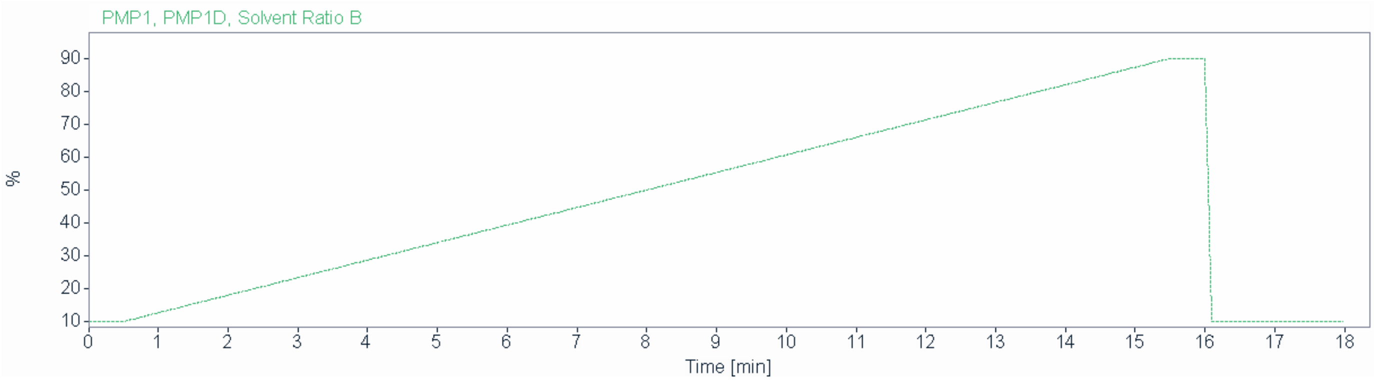

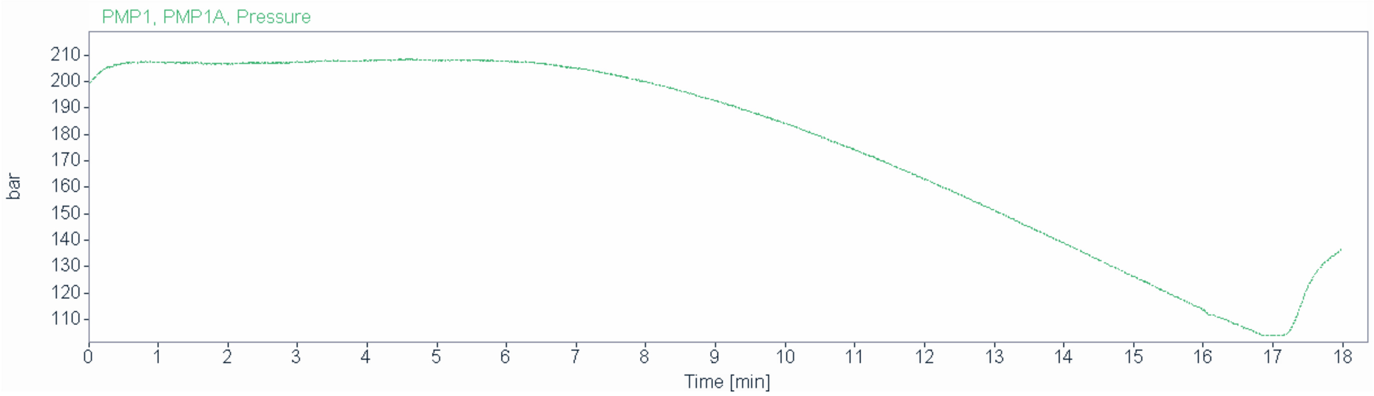

Agilent Technologies
