# Supplementary material for: Dissecting the sequence determinants for dephosphorylation by the catalytic subunits of phosphatases PP1 and PP2A
Source: Nat Commun. 2020 Jul 17;11:3583. doi: 10.1038/s41467-020-17334-x (PMC7367873; doi:10.1038/s41467-020-17334-x)

Single Injection Report

Agilent Technologies

Sample Name JC-131-4-1+2

Injection Acquired Date 9/20/2018 4:17:43 PM

Sample Description

Injection Acq Method Name JC 10 to 90 ACN over 15 min.M

Injection Data File Directory D:\Data\Old Data\Jeremy\Jeremy Template 2018-09-20 16-16-26

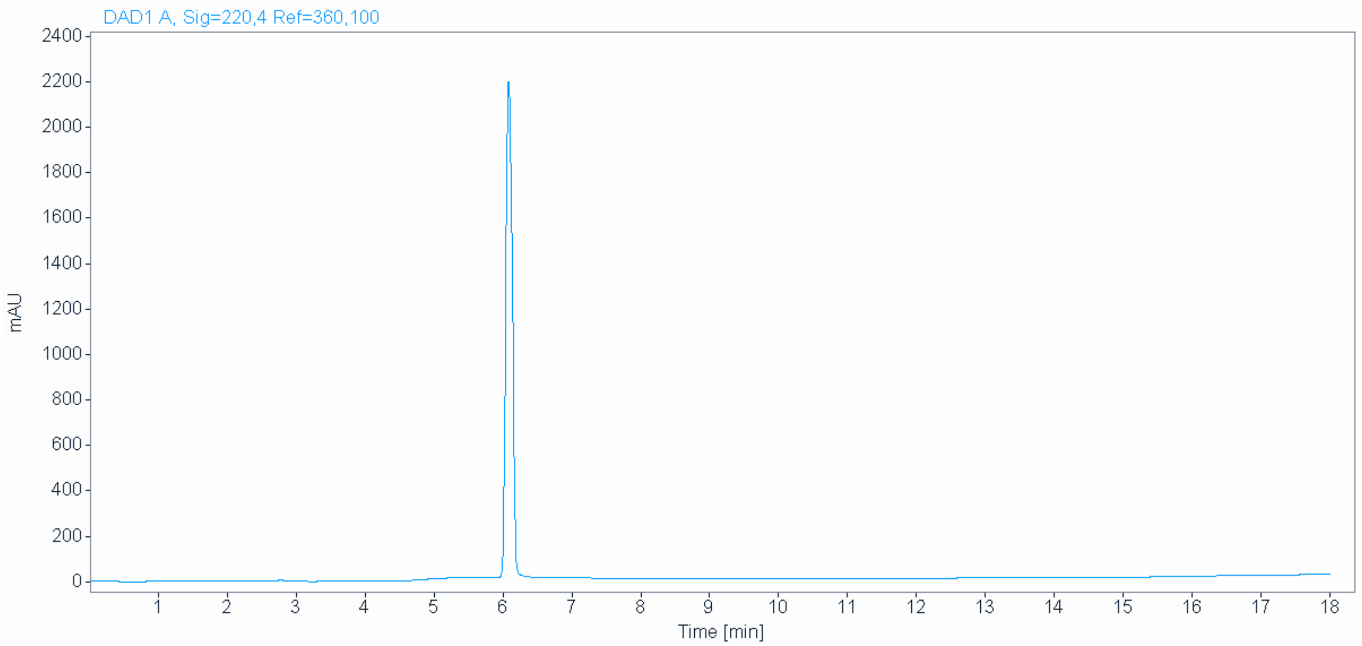

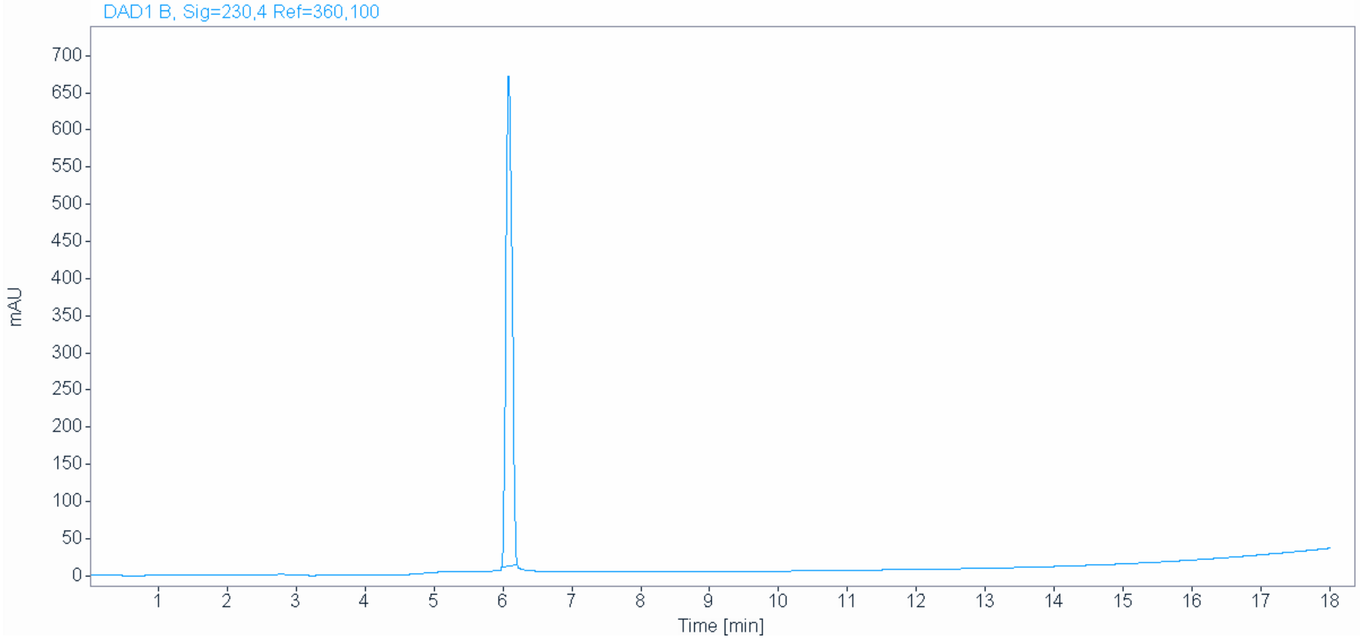

Single Injection Report

Agilent Technologies

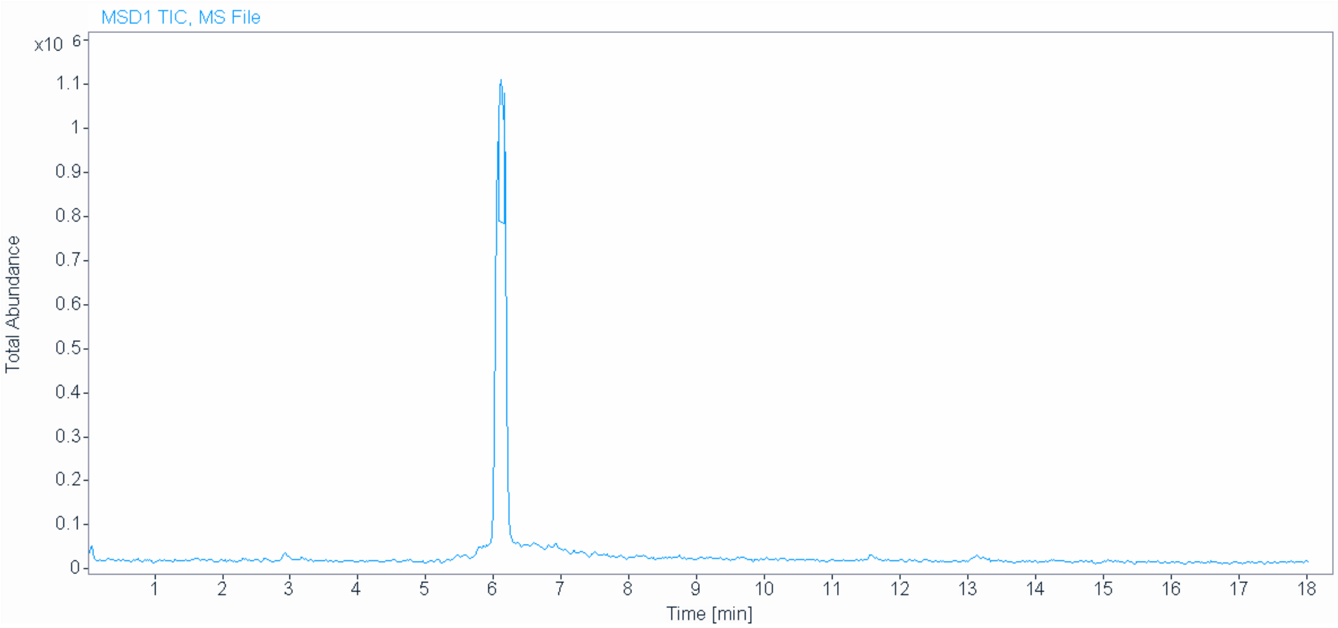

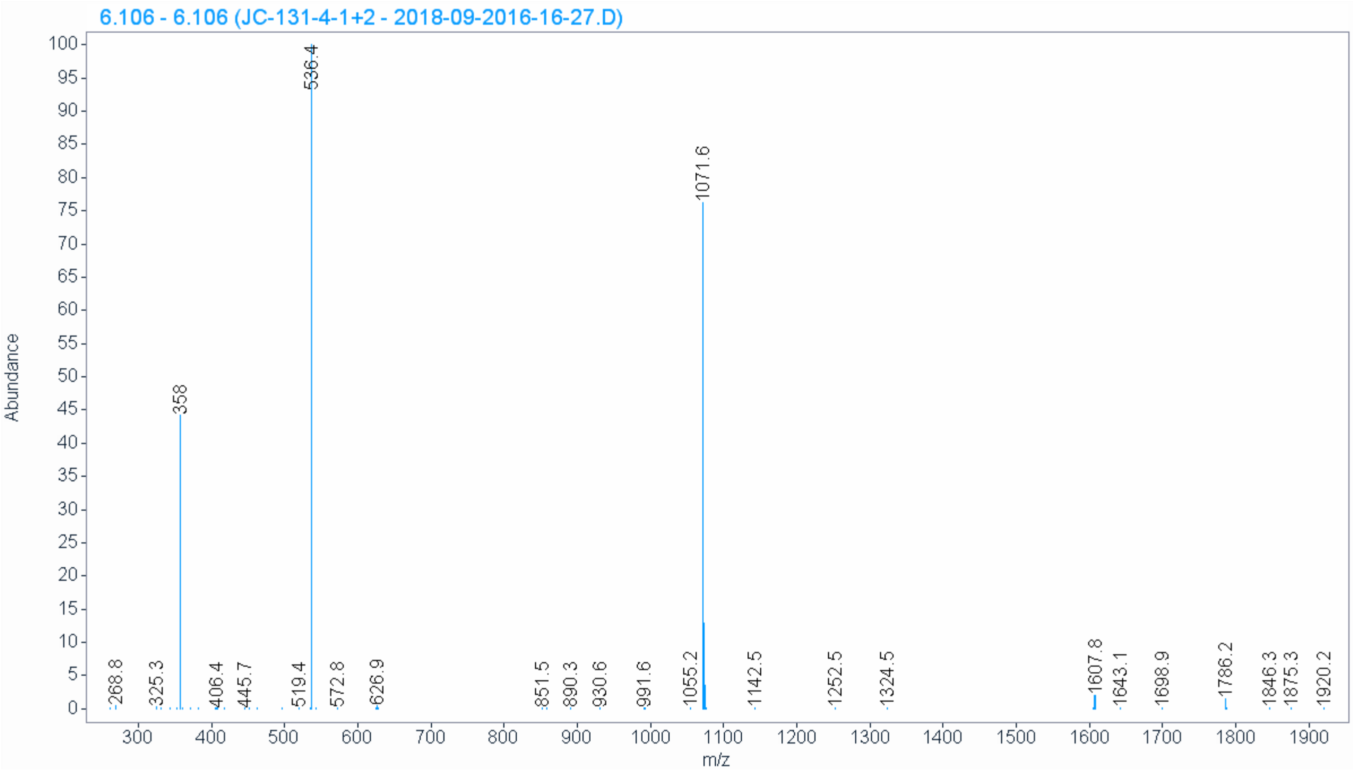

Agilent Technologies

Signal: DAD1 B, Sig=230,4 Ref=360,100

RT [min]	Type	Width [min]	Area	Height	Area%	Name
6.082	MM	0.1017	4040.5481	662.1705	100.0000	
		Sum	4040.5481			

Signal: MSD1 TIC, MS File

RT [min]	Type	Width [min]	Area	Height	Area%	Name
6.106	MM	0.0742	1442399.000 0	323995.1563	100.0000	
		Sum	1442399.000			

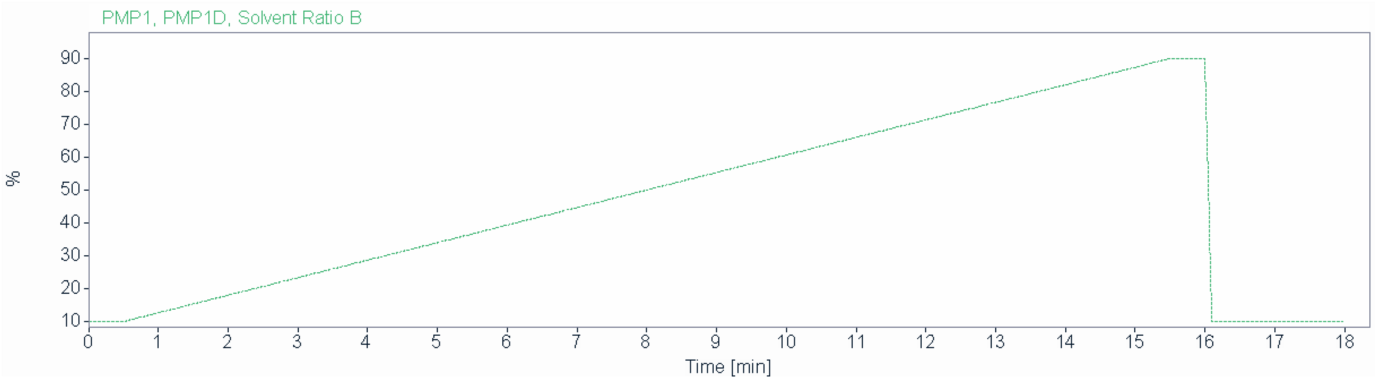

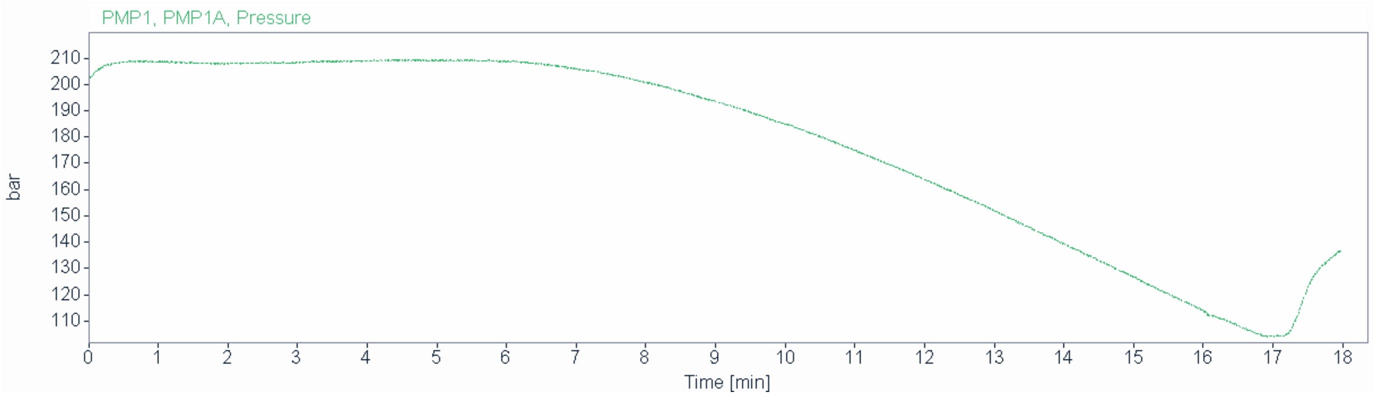

Supplement: Supplementary file 17 — Source Data [file 41467_2020_17334_MOESM17_ESM.zip › SourceData/PeptideSynthesis/PLDMS_verification/FLAKpTAAAAK_report.pdf]
