# Supplementary material for: Dissecting the sequence determinants for dephosphorylation by the catalytic subunits of phosphatases PP1 and PP2A
Source: Nat Commun. 2020 Jul 17;11:3583. doi: 10.1038/s41467-020-17334-x (PMC7367873; doi:10.1038/s41467-020-17334-x)

Single Injection Report

Agilent Technologies

Sample Name	JC-131-2-1+2
Injection Acquired Date	9/18/2018 4:49:36 PM
Sample Description	
Injection Acq Method Name	JC 5 to 20 ACN over 15 min.M
Injection Data File Directory	D:\Data\Old Data\Jeremy\Jeremy Template 2018-09-18 16-28-53

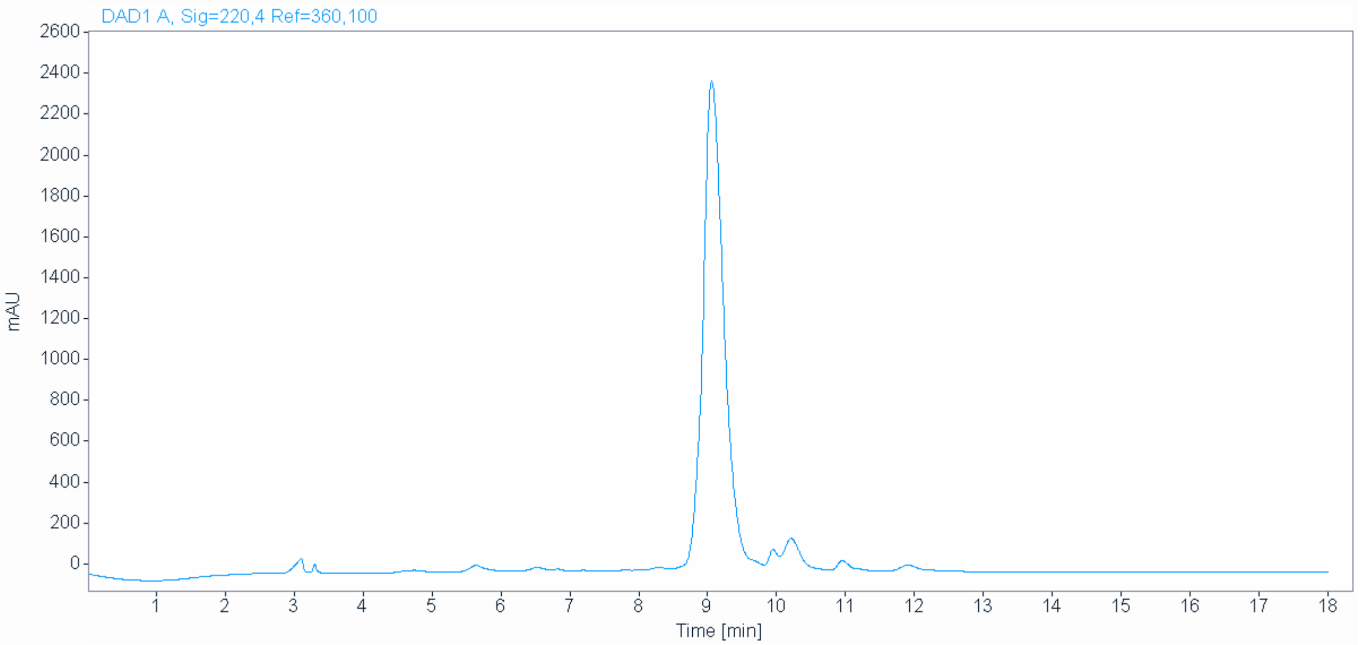

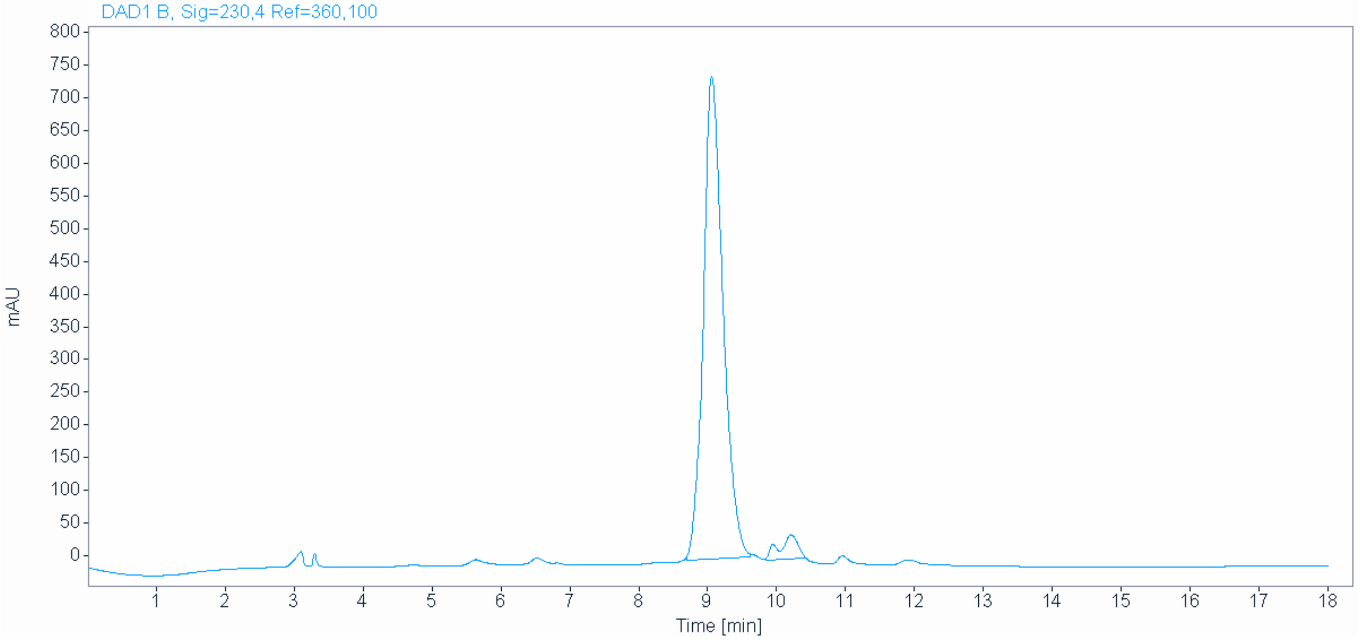

Single Injection Report

Agilent Technologies

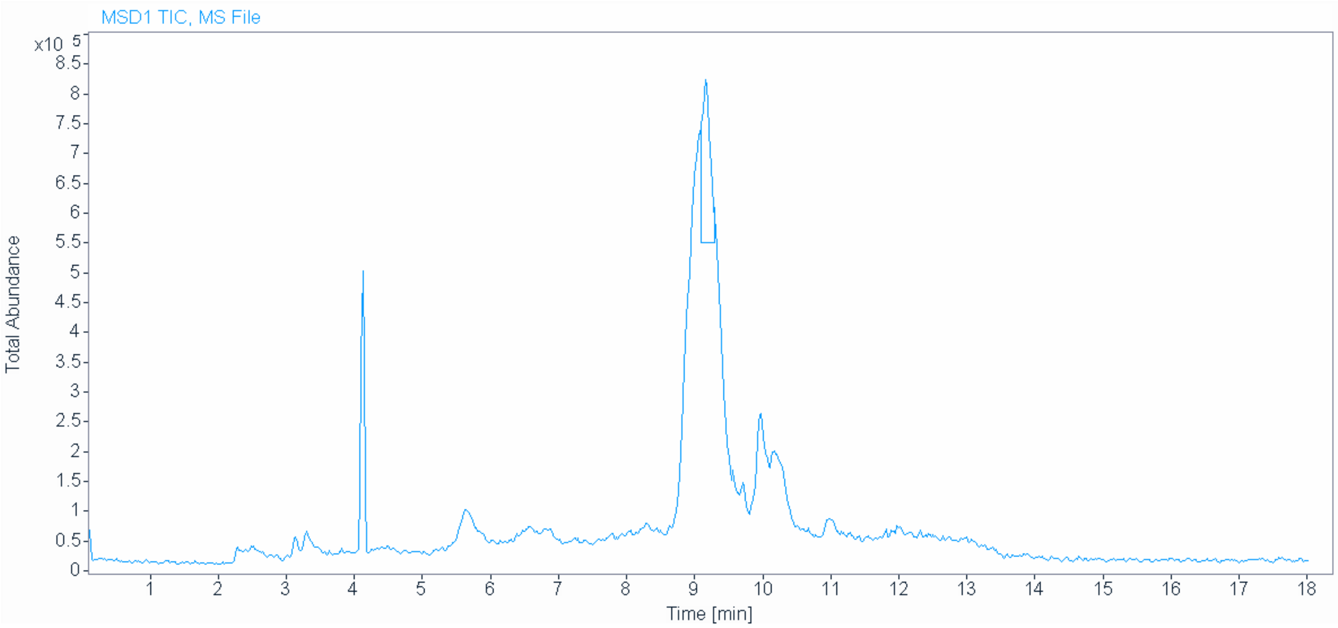

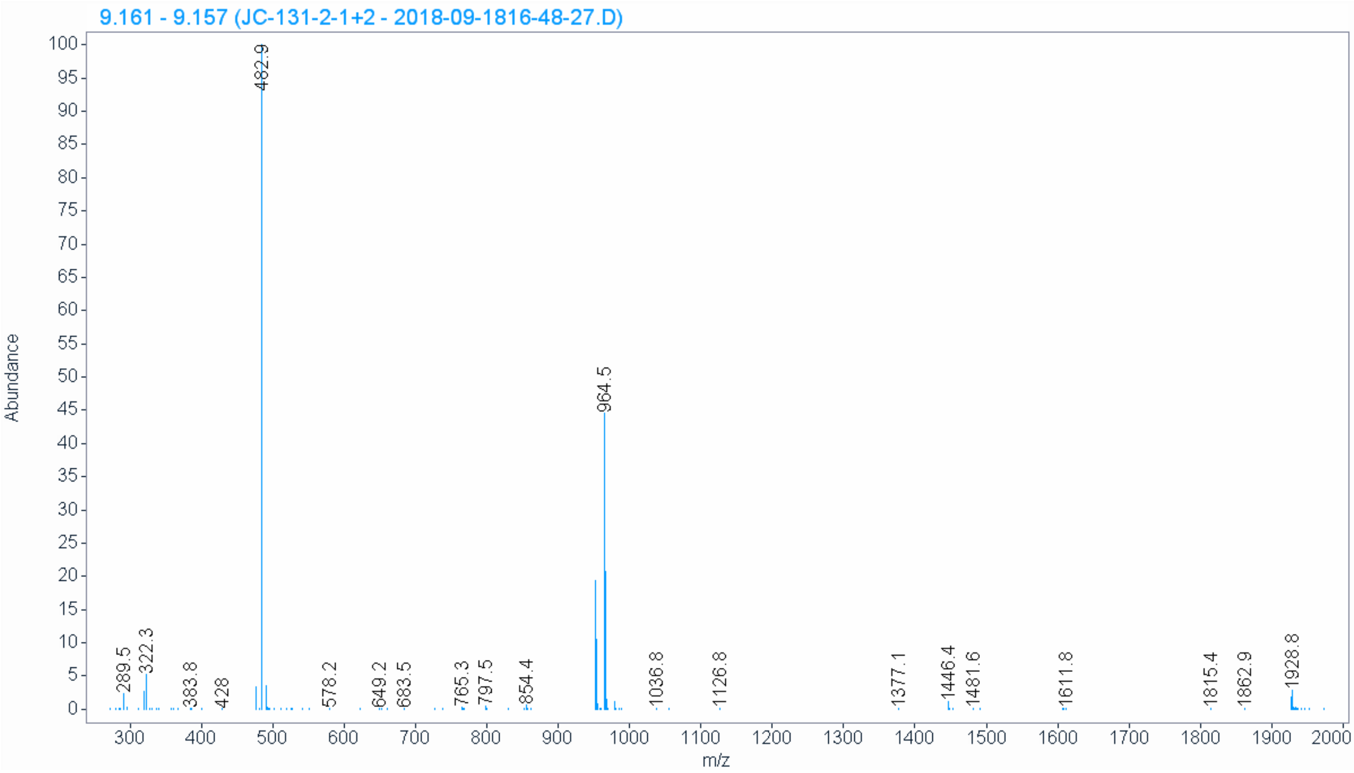

Single Injection Report

Agilent Technologies

Signal: DAD1 B, Sig=230,4 Ref=360,100

RT [min]	Type	Width [min]	Area	Height	Area%	Name
9.057	MM	0.3306	14620.6279	737.1013	95.5215	
10.211	MM	0.3088	685.4905	36.9917	4.4785	
		Sum	15306.1185			

Signal: MSD1 TIC, MS File

RT [min]	Type	Width [min]	Area	Height	Area%	Name
9.161	MM	0.1284	2115051.7500	274481.2188	100.0000	
		Sum	2115051.750			

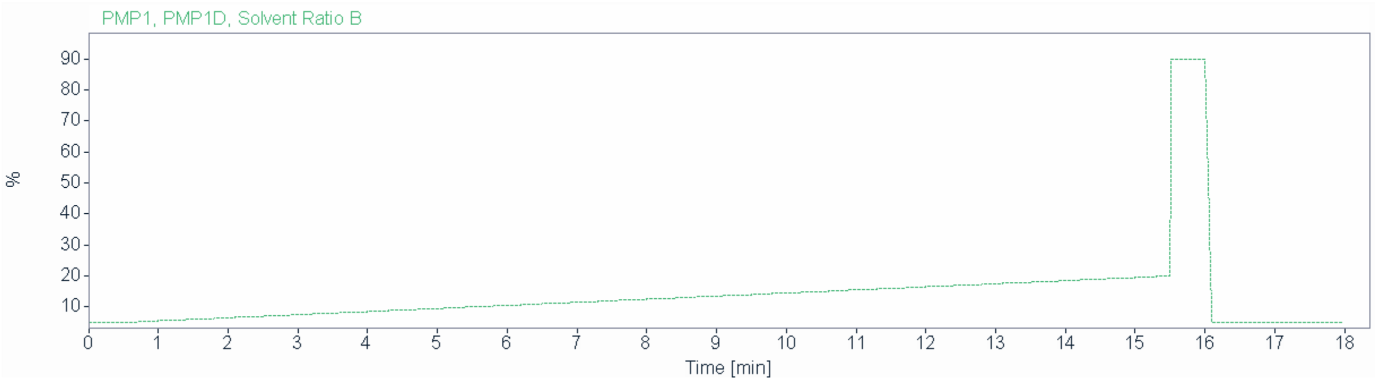

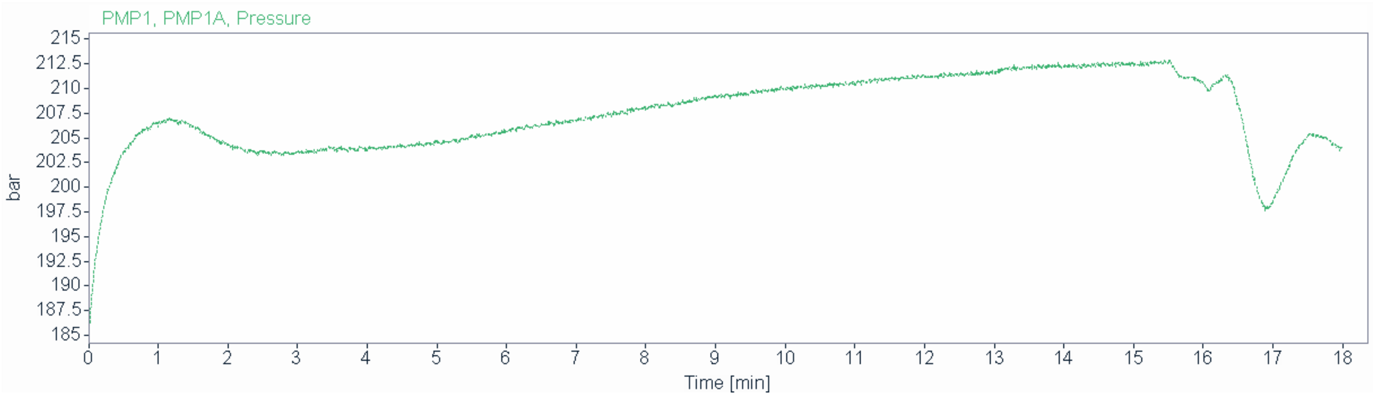

Supplement: Supplementary file 17 — Source Data [file 41467_2020_17334_MOESM17_ESM.zip › SourceData/PeptideSynthesis/PLDMS_verification/TPAPpSAAAAK_report.pdf]
