# Supplementary material for: Dissecting the sequence determinants for dephosphorylation by the catalytic subunits of phosphatases PP1 and PP2A
Source: Nat Commun. 2020 Jul 17;11:3583. doi: 10.1038/s41467-020-17334-x (PMC7367873; doi:10.1038/s41467-020-17334-x)

Single Injection Report

Agilent Technologies

Sample Name	JC-131-1-1+2
Injection Acquired Date	9/18/2018 4:30:17 PM
Sample Description	
Injection Acq Method Name	JC 5 to 20 ACN over 15 min.M
Injection Data File Directory	D:\Data\Old Data\Jeremy\Jeremy Template 2018-09-18 16-28-53

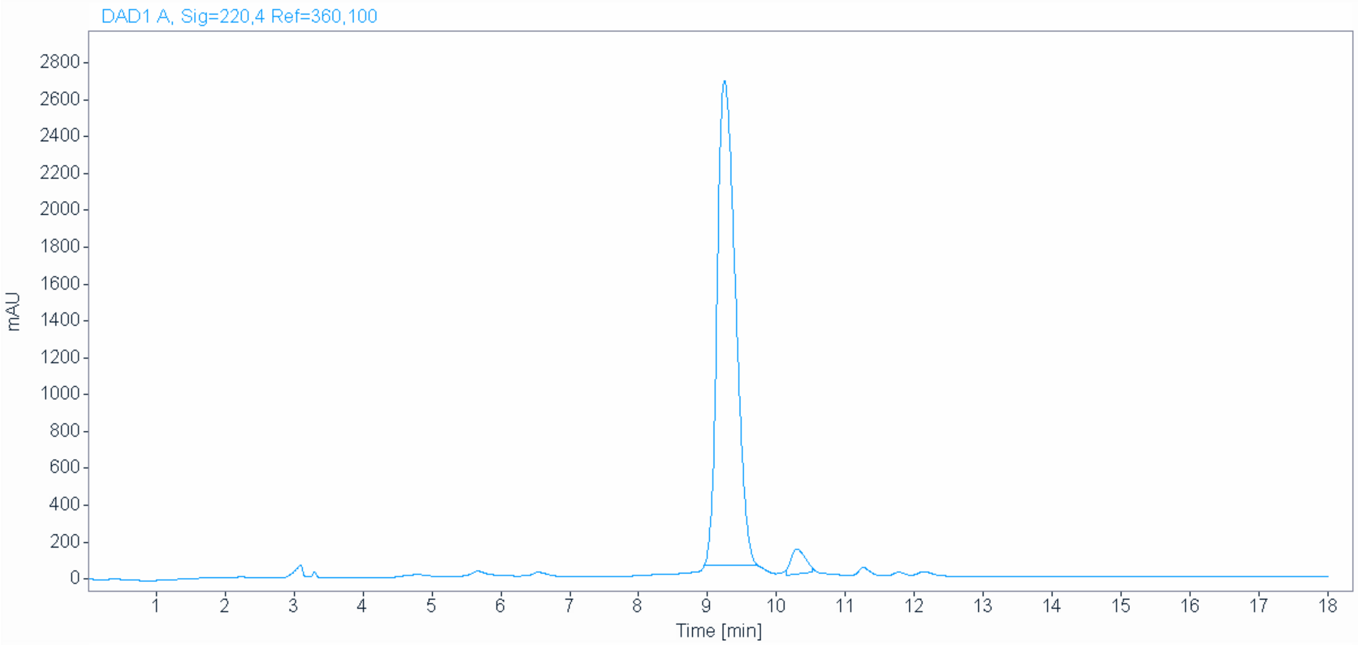

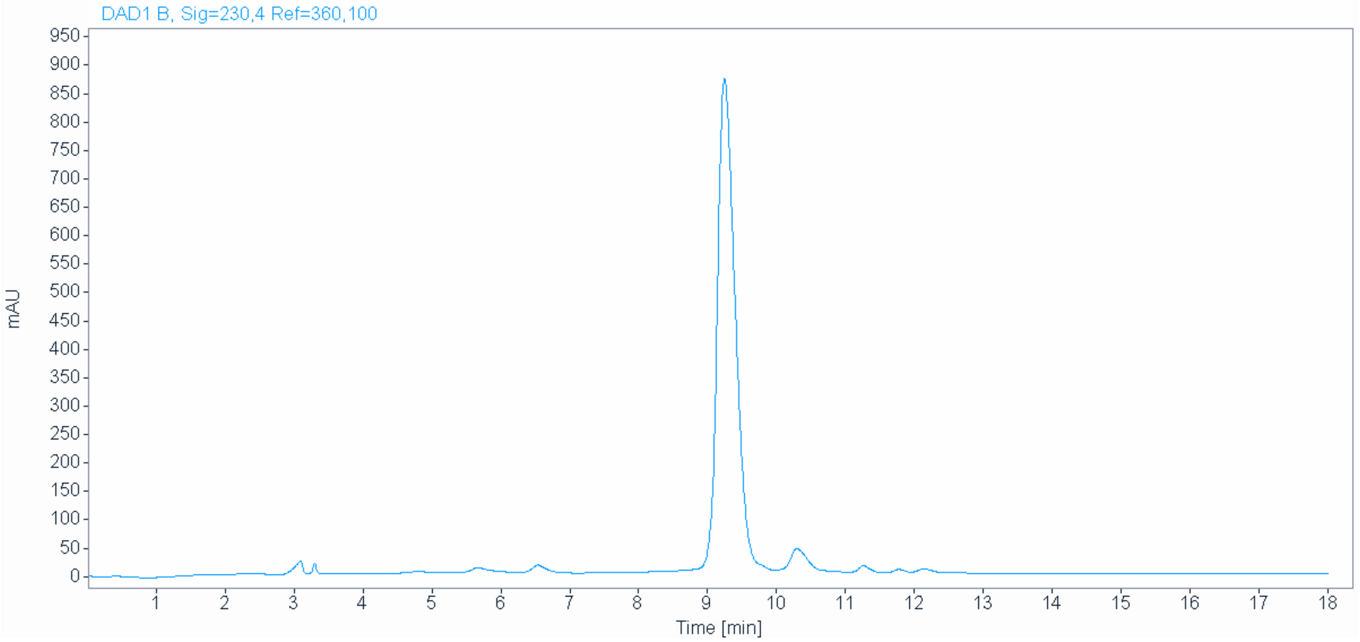

Agilent Technologies

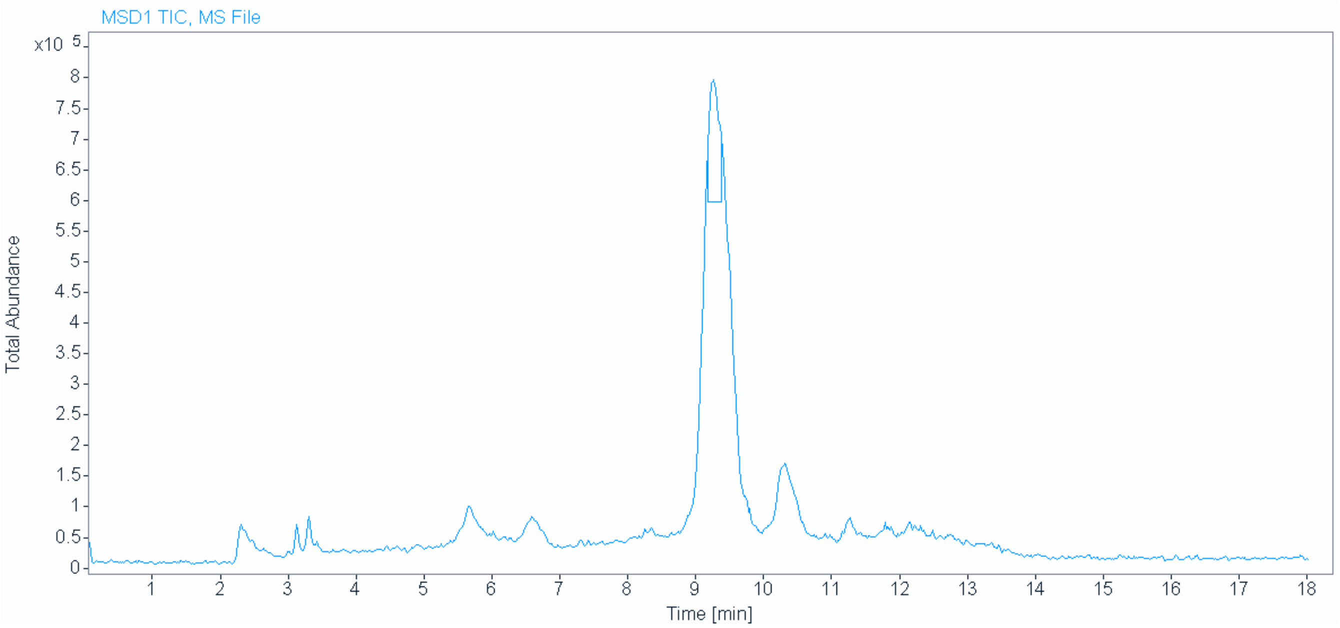

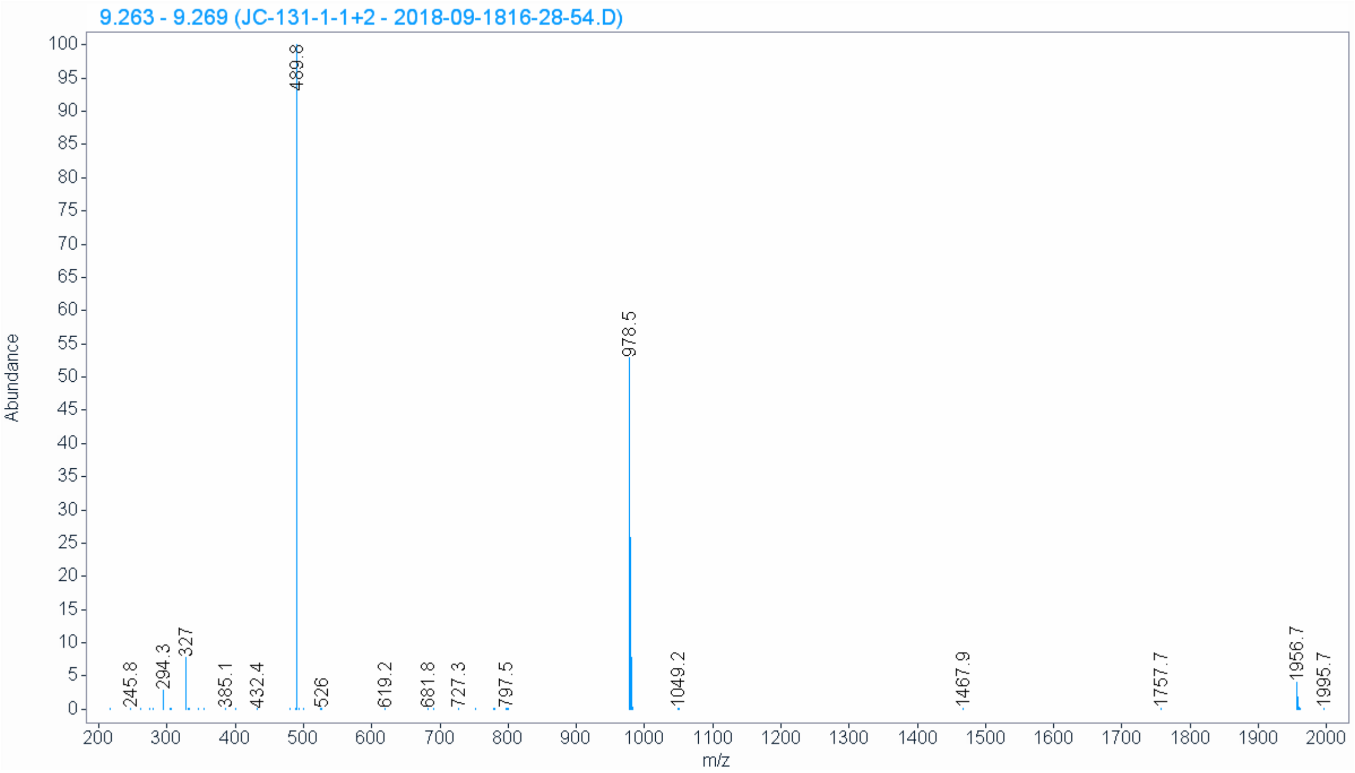

Signal: DAD1 A, Sig=220,4 Ref=360,100

RT [min]	Type	Width [min]	Area	Height	Area%	Name
9.244	MM	0.3065	48352.5586	2629.6399	96.1060	
10.298	MM	0.2417	1959.1115	135.0712	3.8940	
		Sum	50311.6700			

Signal: MSD1 TIC, MS File

RT [min]	Type	Width [min]	Area	Height	Area%	Name
9.263	MM	0.1581	1898291.625 0	200067.5000	100.0000	
		Sum	1898291.625			

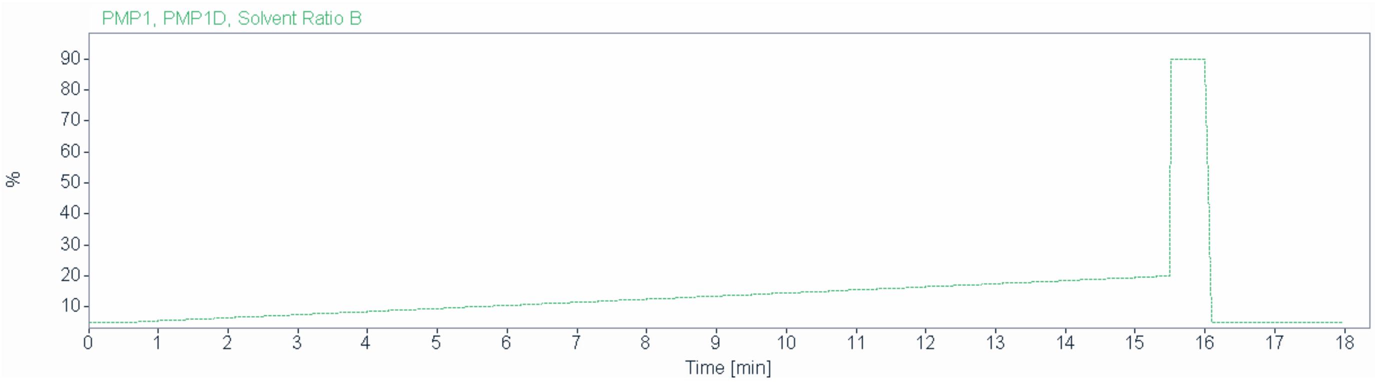

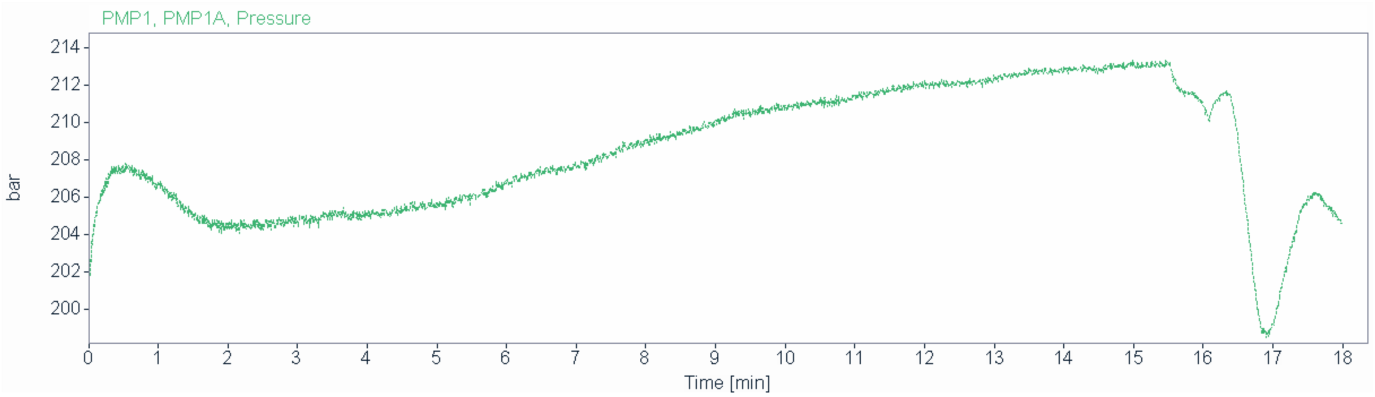

Supplement: Supplementary file 17 — Source Data [file 41467_2020_17334_MOESM17_ESM.zip › SourceData/PeptideSynthesis/PLDMS_verification/TPAPpTAAAAK_report.pdf]
